# Metabolites of lactic acid bacteria present in fermented foods are highly potent agonists of human hydroxycarboxylic acid receptor 3

**DOI:** 10.1371/journal.pgen.1008145

**Published:** 2019-05-23

**Authors:** Anna Peters, Petra Krumbholz, Elisabeth Jäger, Anna Heintz-Buschart, Mehmet Volkan Çakir, Sven Rothemund, Alexander Gaudl, Uta Ceglarek, Torsten Schöneberg, Claudia Stäubert

**Affiliations:** 1 Rudolf Schönheimer Institute of Biochemistry, Faculty of Medicine, Leipzig University, Leipzig, Germany; 2 Department of Internal Medicine, Division of Rheumatology, Leipzig University, Leipzig, Germany; 3 German Centre for Integrative Biodiversity Research (iDiv) Halle-Jena-Leipzig, Leipzig, Germany; 4 Helmholtz-Centre for Environmental Research GmbH - UFZ, Department of Soil Ecology, Halle (Saale), Germany; 5 Core Unit Peptide-Technologies, Leipzig University, Leipzig, Germany; 6 Institute for Laboratory Medicine, Clinical Chemistry and Molecular Diagnostics, University Hospital Leipzig, Leipzig, Germany; University of California Berkeley, UNITED STATES

## Abstract

The interplay of microbiota and the human host is physiologically crucial in health and diseases. The beneficial effects of lactic acid bacteria (LAB), permanently colonizing the human intestine or transiently obtained from food, have been extensively reported. However, the molecular understanding of how LAB modulate human physiology is still limited. G protein-coupled receptors for hydroxycarboxylic acids (*HCAR*) are regulators of immune functions and energy homeostasis under changing metabolic and dietary conditions. Most mammals have two *HCAR* (HCA_1_, HCA_2_) but humans and other hominids contain a third member (HCA_3_) in their genomes. A plausible hypothesis why HCA_3_ function was advantageous in hominid evolution was lacking. Here, we used a combination of evolutionary, analytical and functional methods to unravel the role of HCA_3_ *in vitro* and *in vivo*. The functional studies included different pharmacological assays, analyses of human monocytes and pharmacokinetic measurements in human. We report the discovery of the interaction of D-phenyllactic acid (D-PLA) and the human host through highly potent activation of HCA_3_. D-PLA is an anti-bacterial metabolite found in high concentrations in LAB-fermented food such as Sauerkraut. We demonstrate that D-PLA from such alimentary sources is well absorbed from the human gut leading to high plasma and urine levels and triggers pertussis toxin-sensitive migration of primary human monocytes in an HCA_3_-dependent manner. We provide evolutionary, analytical and functional evidence supporting the hypothesis that HCA_3_ was consolidated in hominids as a new signaling system for LAB-derived metabolites.

## Author summary

Although it has been known for 15 years that HCA_3_ is present in humans and other hominids but absent in all other mammals, no study so far aimed to understand why HCA_3_ was functionally preserved during evolution. Here, we take advantage of evolutionary analyses which we combine with functional assays of hominid HCA_3_ orthologs. In search for a reasonable scenario explaining the accumulated amino acid changes in HCA_3_ of hominids we discovered D-phenyllactic acid (D-PLA), a metabolite produced by lactic acid bacteria (LAB), as the so far most potent agonist specifically activating HCA_3_. Further, oral ingestion of Sauerkraut, known to contain high levels of D-PLA, caused subsequent plasma concentrations sufficient to activate HCA_3_. Our data interpreted in an evolutionary context suggests that the availability of a new food repertoire under changed ecological conditions triggered the fixation of HCA_3_ which took over new functions in hominids.

These findings are particularly important because they unveiled HCA_3_, which is not only expressed in various immune cells but also adipocytes, lung and skin, as a player that transfers signals of LAB-derived metabolites into a physiological response in humans. This opens up new directions towards the understanding of the versatile beneficial effects of LAB and their metabolites for humans.

## Introduction

The interplay of microbiota and the human host is physiologically crucial in health and diseases. Lactic acid bacteria (LAB) are microorganisms present in many foods and the intestine of most mammals. There are extensive reports about the beneficial role of LAB on the immune system [1]. Short-chain fatty acids (SCFAs) and lactate are known metabolites of LAB that have been shown to play an important role in the maintenance of the gut barrier function [2]. SCFAs can induce effects in the host through activation of specific G protein-coupled receptors (GPCRs) expressed in intestinal epithelial cells and immune cells that are located in the intestinal mucosa [3].

The present study focuses on GPCRs belonging to the family of hydroxycarboxylic acid receptors (*HCAR*) which are regulators of immune functions and energy homeostasis under changing metabolic and dietary conditions. At least two *HCAR* subtypes are present in mammalian genomes.

HCA_1_ (formerly GPR81) is activated by lactate and HCA_2_ (formerly GPR109a, HM74A, PUMA-G) is activated by the ketone body D-3-hydroxybutyrate (D-3HB) but also by the SCFA butyrate. Both receptors mediate anti-lipolytic effects in adipocytes through G_i_-protein coupling [4]. Further, HCA_2_ is known to be expressed in enterocytes, colonocytes and several types of immune cells including neutrophils and macrophages mediating anti-inflammatory effects [3].

**Fig 1:**
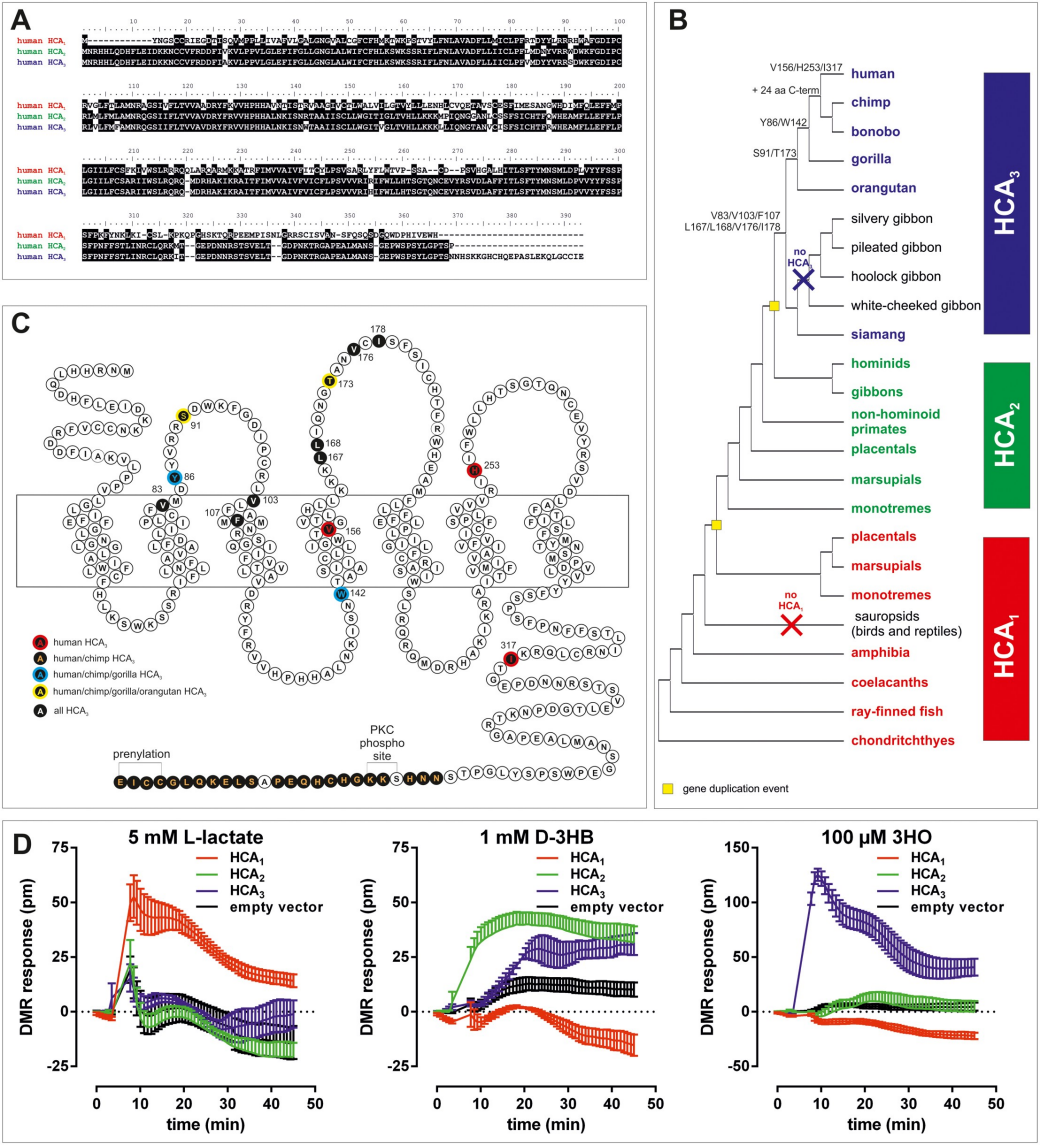
Phylogeny and evolutionary history of *HCAR* and agonist specificity of human HCA_1_, HCA_2_ and HCA_3_. (A) Human HCA_1_ vs HCA_2_ and human HCA_2_ vs HCA_3_ show 50% and 95% amino acid identity, respectively. (B) Schematic *HCAR* phylogeny in vertebrates indicating the appearance of HCA_3_-specific amino acid positions and structural features (detailed tree in S1 Fig). (C) Snake-plot of human HCA_3_ with positions highlighted that are characteristic for HCA_3_ in comparison to HCA_2_ orthologs (detailed alignment in S3 Fig). (D) CHO-K1 cells were transiently transfected with human HCA_1_, HCA_2_, HCA_3_, and empty vector (control) and seeded in fibronectin-coated Epic plates. DMR responses were recorded upon stimulation with 5 mM lactate, 1 mM D-3-hydroxybutyrate (D-3HB) and 100 μM of 3 hydroxyoctanoate (3HO). Shown is the agonist-induced shift in pm as mean ± SEM of four independent experiments carried out in triplicates.

A third *HCAR* subtype, HCA_3_ (formerly GPR109b, HM74), was recently identified in the human genome but is absent in the mouse genome [5]. The amino acid sequence of the human HCA_3_ differs from HCA_2_ in 16 positions and an extended C terminus (Fig 1A). These differences are sufficient to change agonist specificity of HCA_3_ towards being activated by the fatty acid β-oxidation intermediate 3-hydroxyoctanoate (3HO), but not by D-3HB, likely also mediating anti-lipolytic effects under fasting conditions [6]. Further, aromatic D-amino acids were found to activate HCA_3_ and elicit chemotactic responses in human neutrophils [7]. However, a plausible hypothesis why HCA_3_ function is of advantage in humans is currently missing.

Here, we reconstructed the evolutionary history of *HCAR*s and experimentally show that HCA_3_ is functionally present in humans and all other great apes. After gene duplication and distinct structural changes HCA_3_ gained the ability to recognize 3HO as an agonist but lost D-3HB specificity. Moreover, we identified D-phenyllactic acid (D-PLA), a metabolite produced by LAB as the so far most potent naturally occurring agonist acting at HCA_3_. High levels of D-PLA can be found in LAB-fermented products, such as Sauerkraut (S1 Table). LAB fermentation is an ancient process that happened even before humans took advantage of it. It is known that global transitions affected the last common ancestor of early hominoids causing changes in its diet, rendering ingested fruits and leaves more likely to be fermented before ingestion [8]. We provide functional and phylogenetic evidence supporting the hypothesis that increased intake of LAB-fermented food likely posed a positive selective pressure maintaining HCA_3_ function in hominids. We further hypothesize that HCA_3_ presence was advantageous in the interplay between ingested and gastrointestinal microbiome and the hominid host by taking over functions in the immune system.

## Results

### HCA_3_ arose from a duplication of HCA_2_ before the split of great apes and gibbons

Feedback regulation of the energy metabolism is vital for organisms exposed to variable dietary supply. Receptors for intermediates of the energy metabolism, such as hydroxycarboxylic acids, already appeared in early vertebrate evolution [9]. Mining of public sequence databases revealed the presence of at least one HCA_1_ ortholog in the genome of cartilaginous, lobe- and ray-finned fishes, amphibians, and mammals but not in any sauropsidian species (birds, reptiles) (Fig 1B, S1 Fig, S2 Table). HCA_2_ is present in all mammals and arose from an HCA_1_ gene duplication in early mammalian evolution (Fig 1B, S2 Fig, S2 Table).

We found that HCA_3_, the evolutionarily youngest *HCAR*, is present in the genomes of all great apes and siamang, but absent in all other gibbon genomes investigated so far [10]. Because automated genome assembly can cause problems in assigning highly homologous sequences, we manually reanalyzed the genomic sequence traces for the presence of HCA_2_ and HCA_3_ and verified the findings by amplifying, cloning and sequencing *HCAR* from great apes, siamang, and white-cheeked gibbon. Our analysis showed that HCA_3_ arose from a duplication of HCA_2_ before the split of great apes and gibbons (Fig 1B) but underwent pseudogenization in most gibbon species.

Subtypes resulting from gene duplication can have several fates, which include but are not limited to pseudogenization or gain of new function. In the latter case one copy may accumulate mutations and acquire unique functionality without risking the fitness of the organism, which is ensured by the remaining homolog. To test whether the persistence of HCA_3_ in great apes and some gibbons caused changes in evolutionary constraints of *HCAR* subtypes we performed Phylogenetic Analysis by Maximum Likelihood (PAML) [11]. Ape HCA_1_ evolved with an evolutionary rate (ω = 0.189) significantly (p = 0.0258) different compared to all other mammalian HCA_1_ (ω_0_ = 0.105) whereas no significant difference in evolutionary constraint of ape HCA_2_ compared to other mammalian HCA_2_ orthologs was detected (S3 Table). We further tested whether HCA_3_ evolved with a different evolutionary rate than HCA_1_ and HCA_2_ in species carrying all three *HCAR* subtypes. We found that HCA_3_ evolved with a significantly (p = 0.0064) higher evolutionary rate (ω = 0.257) than HCA_1_ and HCA_2_ (ω = 0.103), but significantly different from 1 (S3 Table).

Our data indicates that HCA_3_ is not drifting into neutrality (pseudogenization) in great apes but rather gained new functionality, a hypothesis we further addressed using functional analyses.

### Functional analyses of hominoid *HCAR* reveal evolutionary conservation of known endogenous agonists

To study whether structural differences contribute to the distinct functional properties of the three *HCAR* we heterologously expressed the human HCA_1_, HCA_2_ and HCA_3_ in CHO-K1 cells and performed functional assays using the dynamic mass redistribution technology (DMR; Corning Epic System). Compared to classical second messenger assays, DMR assays provide the advantage that cellular responses are recorded time-resolved and independently of the activated signaling cascades. Receptor activation is monitored kinetically, thus revealing potential differences in activation kinetics mediated by different agonists.

As reported, lactate only activates HCA_1_ [12, 13], D-3HB activates HCA_2_ and with a delayed signal onset also HCA_3_ [14] and 3HO activates HCA_3_ but not HCA_2_ (Fig 1D) [6]. All *HCAR* are G_i_ protein-coupled receptors, i.e. activation of the receptor by an agonist leads to inhibition of adenylyl cyclases, thus resulting in a decrease in intracellular cAMP levels. Concentration-response curves of different agonists can be determined using cAMP inhibition assays. This extends the functional characterization since it reveals information about constitutive *HCAR* activity (basal cAMP) and allows the quantification of the agonistic activity at the respective *HCAR* by determination of EC_50_ and E_max_ values. EC_50_ values reflect the potency of an agonist, i.e. the concentration of an agonist required to produce 50% of its maximal effect; the lower the EC_50_, the higher the potency of an agonist. E_max_ (efficacy) is the maximum effect induced by the agonist, i.e. when E_max_ is reached increasing the agonist concentration will not produce a greater magnitude of the effect.

**Fig 2:**
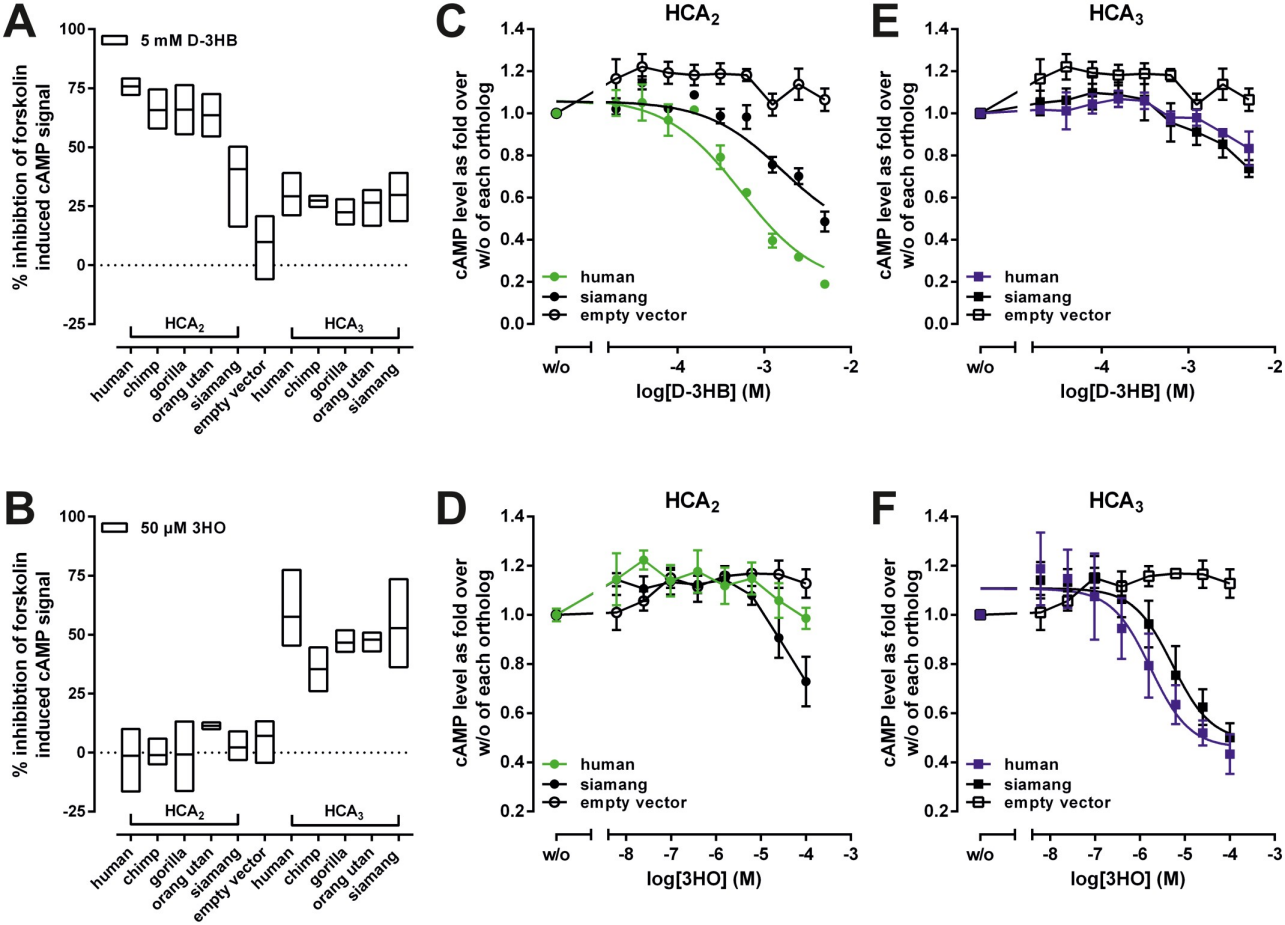
Functional characterization of ape HCA_2_ and HCA_3_ orthologs. CHO-K1 cells were transiently transfected with receptor constructs and agonist-induced inhibition of forskolin-induced cAMP accumulation was determined. HCA_2_ and HCA_3_ orthologs were stimulated with (A) 5 mM D-3-hydroxybutyrate (D-3HB) and (B) 50 μM 3-hydroxyoctanoate (3HO). (A, B) Data is shown as percent inhibition of the forskolin-induced cAMP signal (min to max bars with line at mean). Concentration-response curves upon stimulation of human and siamang HCA_2_ (C, D) and HCA_3_ (E, F) with D-3HB (C, E), and 3HO (D, F) are shown as fold over forskolin-stimulated cAMP level in the absence of agonist of each ortholog. Human HCA_2_ is specificall activated by D-3HB (C), but not the HCA_3_ agonist 3HO (D). In contrast, siamang HCA_2_ is activated by D-3HB with lower potency when compared to the human ortholog (C), but also by 3HO to some extent (D). Data is given as mean ± SEM of at least three independent experiments each carried out in triplicates.

We performed cAMP inhibition assays on all hominoid *HCAR*s as well as mouse HCA_1_ and HCA_2_ and analyzed them with the already established endogenous agonists lactate, D-3HB and 3HO (Table 1, Fig 2). Considering the observed differences in expression levels (Table 2), we detected no significant differences in E_max_ and EC_50_ values upon stimulation with the respective endogenous agonists when comparing within HCA_1_, HCA_2_, and HCA_3_ orthologs (Table 1). However, the fact that D-3HB stimulates HCA_3_ orthologs (Figs 1D, 2A and 2C) to some extent supports its evolutionary origination from HCA_2_. In contrast, 3HO had no activity on HCA_2_ orthologs, indicating a gain of functionality in great ape HCA_3_ after gene duplication (Figs 1D, 2D and 2E).

**Table 1:** Functional characterization of HCA_1_, HCA_2_, and HCA_3_ orthologs. CHO-K1 cells were transfected with receptor constructs and cAMP accumulation was determined. The basal cAMP level of mock-transfected CHO-K1 stimulated with 2 μM forskolin (control) was 41.1 ± 1.9 nmol/well in DMEM and 13.8 ± 1.3 nmol/well in HBSS and set 100%. Analyses of HCA_1_ were carried out in DMEM. HCA_2_ and HCA_3_ were assayed in HBSS. E_max_ values are shown as % of mock-transfected CHO-K1 stimulated with 2 μM forskolin in absence of agonist. E_max_ and EC_50_ values were determined from concentration-response curves using GraphPad Prism. A general explanation of EC_50_ and E_max_ values is provided in the results section *Functional hominoid HCAR analyses with known endogenous agonists*. Data is given as mean ± SEM (basal and E_max_) and geometric mean with 95% confidence interval (EC_50_) of at least three independent experiments each performed in triplicates.

	HCA_1_			HCA_2_			HCA_3_		
	Basal cAMP (% of mock-transfected)	L-lactate		Basal cAMP (% of mock-transfected)	D-3HB		Basal cAMP (% of mock-transfected)	3HO	
		E_max_ (% of mock-transfected w/o agonist)	EC_50_ (mM) (95% CI)		E_max_ (% of mock-transfected w/o agonist)	EC_50_ (mM) (95% CI)		E_max_ (% of mock-transfected w/o agonist)	EC_50_ (μM) (95% CI)
human	66.2 ± 5.2	31.1 ± 2.8	2.4 (1.1–5.1)	260.5 ± 11.8	48.5 ± 6.9	0.53 (0.38–0.74)	56.3 ±1.5	22.2 ± 2.6	1.6 (0.91–2.95)
chimpanzee	73.8 ± 4.5	40.1 ± 2.0	4.1 (3.7–4.6)	239.9 ± 13.9	61.3 ± 3.7	0.77 (0.55–1.1)	51.5 ± 1.6	28.2 ± 1.0	3.7 (2.2–6.1)
gorilla	87.1 ± 5.6	36.7 ± 5.7	2.3 (1.6–3.3)	304.3 ± 18.5	69.5 ± 2.6	0.51 (0.37–0.70)	53.4 ± 1.8	19.7 ± 1.0	2.4 (2.0–3.0)
orangutan	61.2 ± 1.8	35.6 ± 3.7	4.6 (3.7–5.8)	200.7 ± 3.9	65.7 ± 8.8	0.68 (0.60–0.78)	58.0 ± 1.6	31.2 ± 3.6	7.4 (6.9–7.9)
siamang	78.2 ± 1.0	32.1 ± 2.3	5.1 (4.9–5.3)	138.1 ± 6.0	57.3 ± 4.9	1.02 (0.74–1.41)	55.2 ± 1.6	24.2 ± 1.5	2.4 (1.2–4.6)
white-cheeked gibbon	101.9 ± 4.7	28.2 ± 2.6	2.5 (2.0–3.1)	200.1 ± 14.3	50.0 ± 7.8	0.45 (0.31–0.65)	-	-	-
mouse	97.5 ± 0.6	37.1 ± 3.9	2.9 (2.2–3.8)	206.7 ± 4.5	57.3 ± 9.2	1.03 (0.69–1.54)	-	-	-

**Table 2:** Total and cell surface expression levels of HCA_1_, HCA_2_ and HCA_3_ orthologs as determined by ELISA. Cell surface expression levels of mammalian *HCAR* orthologs were measured by a cell surface ELISA. Specific optical density (OD) readings are given as percentage of the respective HA-tagged human *HCAR*. The non-specific OD value (empty vector) was 0.014 ± 0.002 (set 0%). The OD value of the HA-tagged human HCA_1_ (0.091 ± 0.012), of the HA-tagged human HCA_2_ (0.755 ± 0.007) and of the HA-tagged human HCA_3_ (0.424 ± 0.038), each of which was set 100% to compare expression between orthologs. Total expression levels of mammalian *HCAR* orthologs were measured by a sandwich ELISA. Specific optical density (OD) readings are given as a percentage of HA-tagged respective human *HCAR*. The non-specific OD value (empty vector) was 0.011 ± 0.001 (set 0%). The OD value of the HA-tagged human HCA_1_ (0.057 ± 0.012), of the HA-tagged human HCA_2_ (0.591 ± 0.114) and of the HA-tagged human HCA_3_ (0.092 ± 0.006), each of which was set 100%. Data is given as mean ± SEM of three independent experiments carried out in triplicates.

	HCA_1_		HCA_2_		HCA_3_	
	cell surface expression (% of human HCA_1_)	total expression (% of human HCA_1_)	cell surface expression (% of human HCA_2_)	total expression (% of human HCA_2_)	cell surface expression (% of human HCA_3_)	total expression (% of human HCA_3_)
human	100	100	100	100	100	100
chimp	69 ± 3	87 ± 9	83 ± 4	57 ± 4	68 ± 5	68 ± 9
gorilla	59 ± 4	76 ± 3	81 ± 2	56 ± 3	138 ± 11	138 ± 12
orang utan	98 ± 10	41 ± 10	60 ± 9	22 ± 4	141 ± 15	126 ± 6
siamang	110 ± 3	91 ± 9	26 ± 2	20 ± 3	95 ± 6	106 ± 15
white-cheeked gibbon	225 ± 34	113 ± 11	60 ± 6	29 ± 2	-	-
mouse	185 ± 19	139 ± 21	48 ± 1	19 ± 1	-	-

D-3HB activates siamang HCA_2_ with about 3-5-fold lower potency when compared to the human ortholog (Fig 2B) and siamang HCA_2_ is activated by 3HO at high concentrations (Fig 2E). These findings and the fact that HCA_3_ is only present in siamang but not in any other gibbon species initiated further PAML analyses.

### Evolutionary analyses suggest a loss of constraint on siamang HCA_2_ and HCA_3_

We found that HCA_2_ and HCA_3_ of siamang evolve with an ω that is not significantly different from 1 (S3 Table). This indicates a loss of constraint on siamang HCA_2_ and HCA_3_. Further, when compared within all other available gibbon HCA_1_ and HCA_2_ orthologs, we found that siamang HCA_1_ evolves under purifying selection, but indeed siamang HCA_2_ exhibits an ω (ω = 0.643), not significantly different from 1. Thus, we found that, in contrast to all other *HCAR* orthologs, the agonist profiles of siamang HCA_2_ and HCA_3_ are less distinguishable and therefore less conserved.

In sum, our combined evolutionary and functional analyses support a loss of evolutionary constraint on the siamang HCA_2_ and HCA_3_ orthologs (Fig 2, S3 Table).

### Aromatic D-amino acids activate human HCA_3_ with highest potency

Sequence alignment of HCA_3_ orthologs revealed that from all amino acid positions which differ from HCA_2_ seven amino acid positions are conserved in all HCA_3_ orthologs (Fig 1B, 1C and S3 Fig). Further, we found that Ser^91^ and Thr^173^ (referring to positions in human HCA_3_ NP_006009.2) are great ape-specific HCA_3_ positions, while Tyr^86^ and Trp^142^ are only conserved in human, chimpanzee, bonobo, and gorilla HCA_3_ (Fig 1B, 1C and S3 Fig).

Since 3HO activated all hominoid HCA_3_ orthologs with comparable potency, we next analyzed whether potencies of the HCA_3_ agonists D-phenylalanine (D-Phe) and D-tryptophan (D-Trp) [7], vary between orthologs. We confirmed HCA_3_-specificity of both agonists (S4 Fig) and found that both, D-Phe and D-Trp, activate human HCA_3_ with highest potency when compared to all other ape orthologs (Table 3, S4 Fig). Although Irukayama-Tomobe et. al showed both aromatic D-amino acids to be HCA_3_-specific agonists, they did not identify relevant sources of D-Phe and D-Trp to put their findings in a physiological context [7]. By comprehensive research of literature, we here draw the link to fermented foods and beverages which are a likely source for D-Phe and D-Trp in concentrations sufficiently high to activate HCA_3_. Several classes of bacteria produce and secrete aromatic D-amino acids (e.g. *Acetobacter*, *Bifidobacterium*, *Brevibacterium*, *Lactobacillus*, *Micrococcus*, *Propionibacterium*, *Streptococcus*) [15–17] and for all of them residence in the human gastrointestinal tract has been demonstrated [18]. Moreover, D-amino acids were found to be present in body fluids and certain tissues in the μM range [19–22]. Connecting this previously established knowledge led us to further investigate whether other microbial metabolites activate HCA_3_.

**Table 3:** Functional characterization of HCA_3_ orthologs stimulated with aromatic D-amino acids and their metabolites. CHO-K1 cells were transfected with receptor constructs and cAMP accumulation was determined. The basal cAMP level of mock-transfected CHO-K1 stimulated with 2 μM forskolin (control) in HBSS was 13.8 ± 1.3 nmol/well and set 100%. E_max_ values are referred to % of mock-transfected CHO-K1 stimulated with 2 μM forskolin in absence of agonist. E_max_ and EC_50_ values were determined from concentration-response curves of agonists using GraphPad Prism. A general explanation of EC_50_ and E_max_ values is provided in the results section Functional hominoid *HCAR* analyses with known endogenous agonists. Data is given as mean ± SEM (E_max_) and geometric mean with 95% confidence interval (EC_50_) of at least three independent experiments each performed in triplicates.

	E_max_ (% of mock-transfected w/o agonist)	EC_50_ (μM) (95% CI)	E_max_ (% of mock-transfected w/o agonist)	EC_50_ (μM) (95% CI)	E_max_ (% of mock-transfected w/o agonist)	EC_50_ (μM) (95% CI)
	**D-Phe**		**D-Trp**		**3HDec**	
human HCA_3_	21.0 ± 2.3	36.1 (17.7–73.5)	14.7 ± 1.0	20.5 (11.7–36.2)	18.7 ± 2.3	31.9 (22.3–45.8)
chimpanzee HCA_3_	23.4 ± 2.4	37.4 (23.2–60.2)	22.2 ± 2.2	62.1 (47.6–81.0)	25.7 ± 1.2	141(97.5–205)
gorilla HCA_3_	17.9 ± 2.3	45.7 (27.1–77.1)	17.8 ±1.5	24.4 (23.2–25.8)	25.8 ± 2.7	37.3 (23.0–60.3)
orangutan HCA_3_	24.6 ± 2.4	129 (103–161)	24.7 ± 1.5	50.7 (30.8–83.4)	28.4 ± 3.1	62.7 (39.8–98.7)
siamang HCA_3_	22.9 ± 5.2	202 (101–406)	24.3 ± 1.2	56.7 (38.8–82.5)	35.6 ± 1.0	102 (59.9–175)
	**D-PLA**		**L-PLA**		**ILA**	
human HCA_3_	20.5 ± 2.8	0.15 (0.05–0.42)	33.4 ± 2.9	5.2 (3.0–9.1)	32.9 ± 2.9	0.18 (0.08–0.39)
chimpanzee HCA_3_	23.7 ± 1.8	0.13 (0.08–0. 22)	25.4 ± 1.1	4.0 (2.4–6.5)	32.6 ± 4.9	0.23 (0.16–0.33)
gorilla HCA_3_	19.9 ± 1.4	0.085 (0.032–0.21)	36.5 ±3.0	6.2 (4.3–8.9)	36.3 ±1.7	0.073 (0.053–0.10)
orangutan HCA_3_	29.3 ± 3.3	1.2 (0.60–2.2)	39.3 ± 3.2	6.5 (3.1–13.7)	42.0± 1.0	0.068 (0.030–0.15)
siamang HCA_3_	20.4 ± 1.7	0.28 (0.14–0.56)	36.3 ± 3.5	17.2 (8.9–33.6)	33.6 ± 1.2	0.17 (0.11–0.28)

### Lactic acid bacteria derived metabolites are highly potent agonists at HCA_3_

LAB are known to produce metabolites structurally related to 3HO and D-Phe, such as 3-hydroxydecanoate (3HDec) and D-PLA, respectively [23, 24]. PLA has been detected in μM concentrations in fermented food such as Sauerkraut for which remarkably stable microbial associations over time and region have been shown (Literature summarized in S1 Table) [25]. Moreover, LAB represent 0.01–1.8% of the total bacterial community found in the human intestine where some of them are colonizers and others are passengers [26]. Additional support for the link between LAB and D-PLA is provided by analyses of patients with short bowel syndrome (SBS) with their microbiota known to be imbalanced to *Lactobacillus* (*L*. *mucosae*, *L*. *acidophilus*, *L*. *fermentum*) [27, 28] as the major resident bacteria (increased from ≤ 1% up to 60% of the fecal flora). These patients exhibit highly increased urinary levels of D-PLA [29–33].

**Fig 3:**
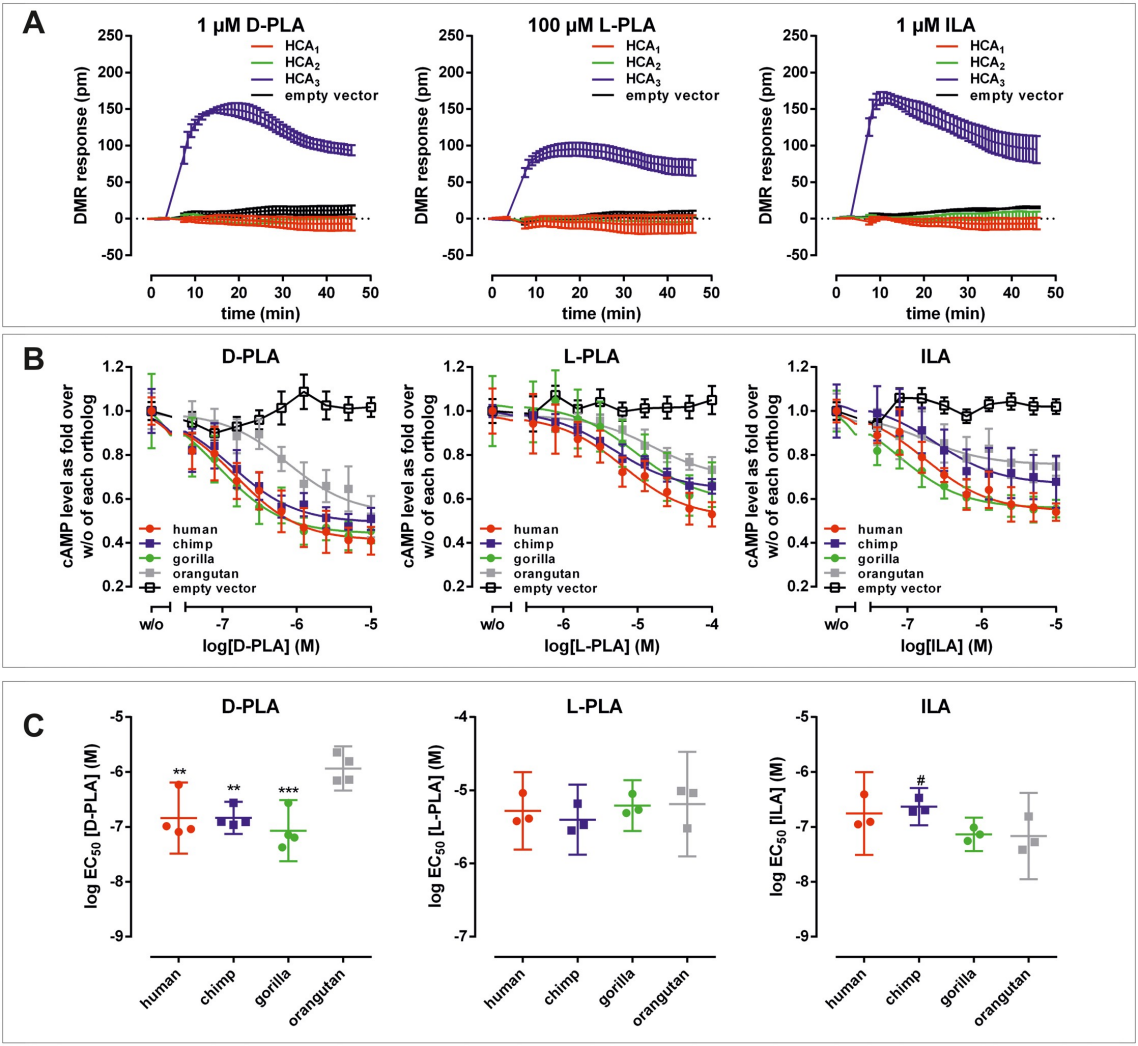
D-Phenyllactic acid is a potent agonist acting at HCA_3_. (A) CHO-K1 cells were transiently transfected with human HCA_1_, HCA_2_, HCA_3_, and empty vector (control), seeded in fibronectin-coated Epic plates and DMR responses were recorded upon stimulation with 1 μM D-phenyllactic acid (D-PLA), 100 μM L-phenyllactic acid (L-PLA) and 1 μM indole lactic acid (ILA) to reveal potential differences in receptor activation kinetics. Shown is the agonist-induced shift in pm of three independent experiments carried out in triplicates. (B) The agonist-induced inhibition of forskolin-induced cAMP accumulation was determined in CHO-K1 cells transiently transfected with HCA_3_ orthologs and empty vector. Concentration-response curves upon stimulation with D-PLA, L-PLA and ILA are depicted as fold over over forskolin-stimulated cAMP level in the absence of agonist of each ortholog. Data is given as mean ± SEM of at least three independent experiments each carried out in triplicates. A general explanation of EC_50_ values is provided in the results section Functional hominoid *HCAR* analyses with known endogenous agonists. (C) log EC_50_ values (corresponding to EC_50_ values shown in Table 3) are plotted as mean ± 95% confidence interval. Statistical analyses were performed using an ordinary One-Way ANOVA (Dunnett’s multiple comparisons test) testing against orang utan HCA_3_. ^#^ ≤ 0.1, * P ≤ 0.05; ** P ≤ 0.01; *** P ≤ 0.001.

To our knowledge, the present study is the first in which D-PLA, L-phenyllactic acid (L-PLA) and indole 3-lactic acid (ILA), a metabolite derived from Trp metabolism, were functionally tested for their agonistic activity at the human HCA_3_. Using the DMR technology we found that all three metabolites activate the human HCA_3_, but not HCA_1_ and HCA_2_ (Fig 3A). D-PLA and ILA (each 1 μM) induced a response comparable to that of 100 μM 3HO, the known endogenous agonist indicating an about 100-fold higher potency of the LAB-derived compounds (Figs 1D and 3A).

Next, we functionally analyzed all HCA_3_ orthologs with the newly identified agonists using cAMP inhibition assays to determine their potencies (Table 3). Unfortunately, the individual potencies of D- and L-ILA could not be determined since only the DL isomeric mixture was commercially available. L-PLA activates HCA_3_ orthologs in the μM range. However, D-PLA activates HCA_3_ with an EC_50_ value of 150 nM, thus being an about 35-fold more potent agonist than the L-enantiomer and about 10-fold and 240-fold more potent than the known agonists 3HO and D-Phe, respectively (Tables 1 and 3).

Further, both D-PLA and D-Phe exhibit higher potencies at the HCA_3_ of human, chimpanzee and gorilla when compared to the orangutan ortholog (Fig 3B, 3C and S4 Fig). In contrast, the more hydrophobic agonists 3HDec, D-Trp and ILA are less potent at the chimpanzee HCA_3_ ortholog when compared to the other great ape HCA_3_ orthologs (Fig 3B, 3C and S4 Fig). The Trp metabolite ILA acted at human HCA_3_ with a potency comparable to that of D-PLA. Thus, D-PLA is the most potent agonist at human HCA_3_ for which sufficient presence in LAB-fermented food has been previously described (S1 Table).

### D-lactate dehydrogenase is abundant in the human gut microbiome

Accumulating evidence suggests that only a small number of LAB species are true inhabitants of the human intestinal tract. The majority of LAB is derived from fermented food, the oral cavity or more proximal parts of the gastrointestinal tract, like the esophagus [34]. However, we asked whether the gene for D-lactate dehydrogenase (EC 1.1.1.28), the enzyme that has been shown to produce D-PLA [35–37], is present in the human microbiome.

We found that genes encoding D-lactate dehydrogenase are very common among bacteria and are also found in some of the very common and abundant gut bacteria (S5 Fig, S4 Table). However, the question remains, if these D-lactate dehydrogenases are capable of converting the aromatic amino acids to the respective lactic acid in the human intestine. Even if this ability is restricted to LAB possessing this gene, they are still detectable to some degree in the human intestinal microbiome [38].

### Oral uptake of pure or Sauerkraut-derived D-PLA results in increased plasma and urine levels

**Fig 4:**
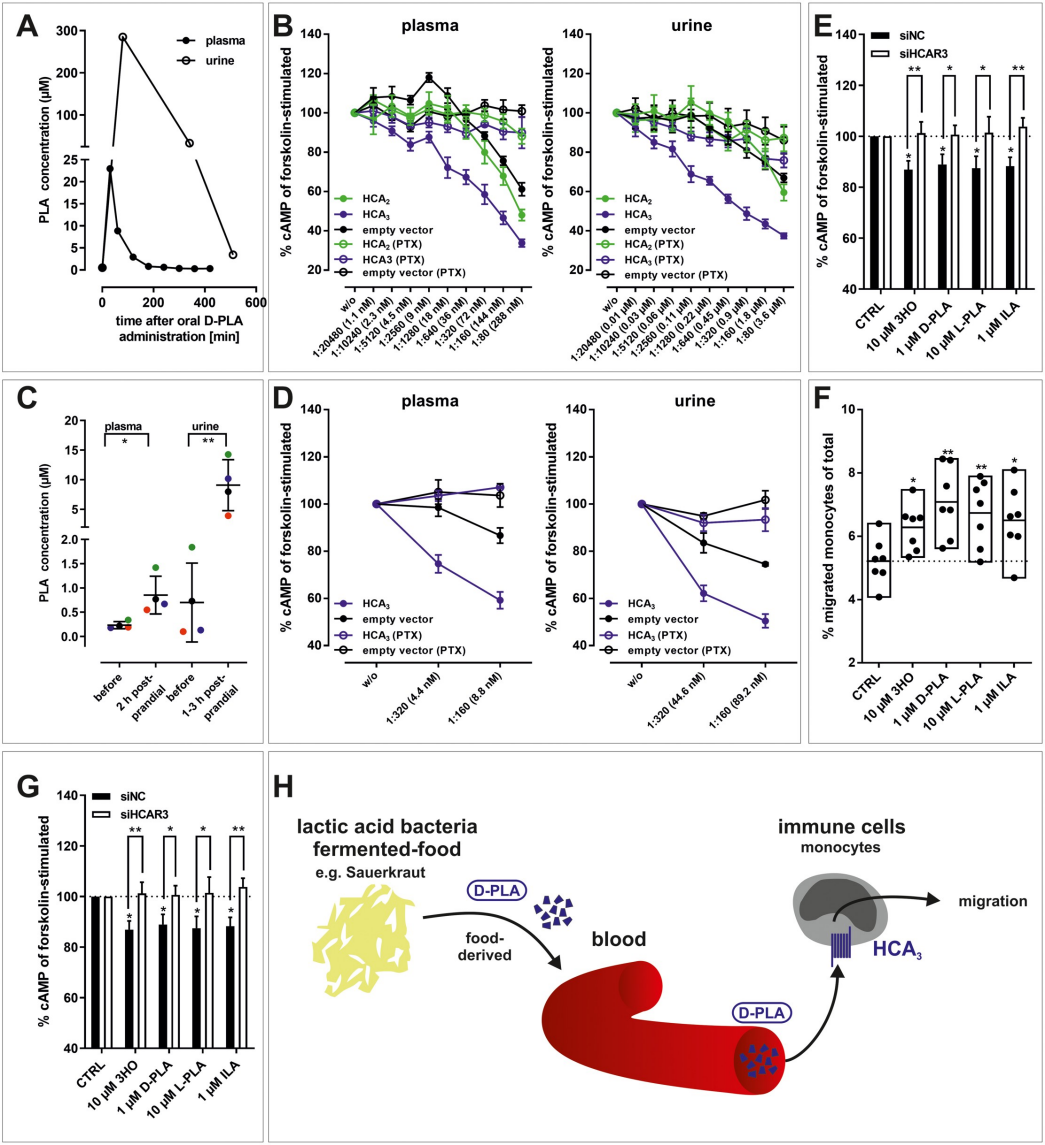
D-PLA is absorbed from the human gut, reaches μM plasma concentrations and activates HCA_3_ in human monocytes. (A) Upon oral ingestion of 100 mg D-PLA (one individual) plasma and urine PLA levels were measured using LC-MS. Details about experimental setup are stated in *Materials and Methods*, section *Determination of PLA in human plasma and urine*. (B) PLA containing plasma (23 μM, corresponding to the 30 min time point from (A)) and urine (285 μM, corresponding to the 80 min time point from (A)) were stepwise 1:2-diluted and tested in cAMP inhibition assays. (C) Upon oral uptake of 5–6 g Sauerkraut per kg body weight (n = 4 individuals, each individual labeled with a different color), plasma and urine PLA levels were measured using LC-MS. Details about experimental setup are stated in *Materials and Methods*, section *Determination of PLA in human plasma and urine*. (D) PLA containing plasma (1.4 μM, 2 h postprandial) and urine (14.3 μM, 3 h postprandial) were stepwise 1:2-diluted and tested in cAMP inhibition assays. (B, D) Data is shown as fold over unstimulated cAMP level, given as mean ± SEM (n = 3) and is summarized in S5 Table. (E) 3HO, D-PLA, L-PLA, and ILA induced a pertussis-toxin (PTX) sensitive reduction in cAMP levels and (F) migratory responses in human monocytes. (E) Data is given as percent of cAMP level in monocytes stimulated with 10 μM forskolin without agonist. The mean ± SEM (n = 3 different donors) is shown. (F) Data is shown as percent migrated monocytes of total monocytes (min to max bars with line at mean, n = 7 different donors). (G) siRNA-mediated knock-down of HCA_3_ in human monocytes diminished the agonist-induced reduction of cAMP levels still present in negative control siRNA (siNC) transfected monocytes. Data is shown as mean ± SEM (n = 7 different donors). (H) D-PLA is absorbed from ingested LAB-fermented food (Sauerkraut) and induces HCA_3_-dependent migration in human monocytes. This opens up new perspectives to study the role of HCA_3_ activation by LAB-derived metabolites in both immune function and energy homeostasis. (E, G) Paired two-tailed t-tests were performed to analyze the effect of PTX and siRNA transfection. (E, F, G) Statistical analyses were performed using an ordinary One-Way ANOVA (Dunnett’s multiple comparisons test) testing against control (CTRL, vehicle EtOH). ^#^ ≤ 0.1, * P ≤ 0.05; ** P ≤ 0.01; *** P ≤ 0.001.

To further substantiate D-PLA resorption from gut and its urinary elimination, a pharmacokinetic study was performed determining PLA levels using Liquid Chromatography Mass Spectrometry (LC-MS) in human plasma and urine samples. As shown in Fig 4A, PLA plasma levels are increased already 30 minutes after oral application of 100 mg D-PLA and subsequently decline rapidly due to renal excretion as determined in concomitantly collected urine samples. PLA plasma levels reached concentrations (>20 μM) capable to maximally activate the human HCA_3_ (Fig 3B). To verify this, we tested whether PLA-containing plasma and urine samples elicit an HCA_3_-specific, pertussis toxin (PTX) sensitive decline in intracellular cAMP levels of transiently transfected CHO-K1 cells (Fig 4B, S5 Table). Thus, plasma and urine samples were diluted and assayed by addition to transfected CHO-K1 cells. Indeed, we found that PLA containing urine and to a lesser extend plasma activated specifically and concentration-dependent HCA_3_ transfected cells (Fig 4B, S5 Table).

Interestingly, we also found basal plasma levels of PLA of about 0.4 μM before oral D-PLA administration (Fig 4A, S6 Fig). Our established LC-MS measurement method does not discriminate between L-PLA and D-PLA. Blood levels of L-PLA in the μM range are only found in patients with phenylketonuria [39]. L-PLA is 35-fold less potent than D-PLA at HCA_3_ (Table 3) and plasma samples from basal conditions (0.4 μM) were sufficient to inhibit forskolin-induced cAMP formation via HCA_3_ (S5 Table), potentially due to presence of D-PLA derived from alimentary microbiota sources.

Sauerkraut is known to contain high levels of LAB as well as D-PLA. A recent study showed that Sauerkraut exhibits remarkably stable microbial associations over time and regions [40]. Therefore, we asked whether ingestion of Sauerkraut (5–6 g per kg body weight) can cause an increase in plasma and urinary D-PLA levels. Using LC-MS we found that levels of PLA in plasma (before = 0.2 μM; after = 0.9 μM) and urine (before = 0.7 μM; after = 9.1 μM) are increased 2 h postprandial in four individuals (Fig 4C, S6A Fig). Further, we found that plasma and urine containing increased concentrations of PLA after Sauerkraut ingestion induced an HCA_3_-specific, PTX-sensitive inhibition of intracellular cAMP levels (Fig 4D, S6B Fig, S5 Table). This clearly indicates that fermented food, such as Sauerkraut, can be a source of D-PLA leading to functionally relevant concentrations of this bacterial metabolite in humans.

### mRNA expression of human HCA_3_ is highest in immune cells

Until recently, mRNA expression datasets based on microarrays constituted the main source for expression data. However, due to their high nucleotide sequence similarity (97%) these datasets did not enable the distinction between human HCA_2_ and HCA_3_. High quality, paired-end, RNA-Sequencing datasets are in principle suitable to distinguish between those highly similar sequences. Indeed, public resources provide quantitative expression data (FPKM: fragments per kilobase million, TPM: transcripts per kilobase million) for both HCA_2_ and HCA_3_. Quantification of transcripts in RNA-Sequencing projects is based on counting small RNA fragments mapped to a gene (read lengths 70–100 bp). Because long nucleotide sequence parts of HCA_2_ and HCA_3_ transcripts are identical it was not clear whether the used bioinformatic tools are precise enough to properly distinguish and quantify human HCA_2_ and HCA_3_ transcripts. Therefore, we manually reanalyzed a publicly available dataset (Bioproject: PRJNA326727) (S6 Table, S7 Fig). We mapped all relevant reads to HCA_2_ or HCA_3_ and excluded those matching to both receptors. We found no significant differences between the provided FPKM values and our manually curated read counts (S7 Fig).

Mining of publicly available RNA-Sequencing data reanalyzed and provided as TPM values revealed highest expression of HCA_3_ in immune cells such as neutrophils and monocytes and a pattern distinct from that of HCA_2_ (S7 Fig, S7 Table). Expression of HCA_3_ mRNA, especially in adipose tissue, skin and lung, is considerably lower compared to HCA_2_ (S7 Fig). However, both receptors are up-regulated in ulcerative colitis and inflammatory bowel disease patients compared to controls (S6 Table, S7 Fig).

### LAB-derived metabolites induce a chemotactic response in human monocytes through activation of HCA_3_

As described above, HCA_3_ is expressed in a variety of human immune cells including macrophages, neutrophils, and monocytes (S7 Table). First, we used freshly isolated human peripheral blood mononuclear cells (PBMCs), which include lymphocytes (T cells, B cells, and NK cells), monocytes, and dendritic cells. We observed a significant reduction in cAMP levels for all HCA_3_ agonists except of 10 μM L-PLA in PBMCs (S6C Fig). However, PTX sensitivity of this reduction could only be observed for 3HO and D-PLA, but not the other HCA_3_ agonists (S6C Fig). We assume that this is likely due to inter-individual variation in cell counts of the different cell types in PBMCs. Thus, we isolated human monocytes and performed cAMP inhibition assays. We found that 10 μM 3HO as well as 1 μM D-PLA and ILA induced a decrease of cAMP in human monocytes that can be blocked by PTX (Fig 4E). Using a transwell migration assay we showed that D-PLA can trigger chemotactic responses with high potency in isolated monocytes (Fig 4F). Both, a PTX-sensitive reduction in cAMP levels and migratory responses were also observed for D-Phe and D-Trp with freshly isolated human monocytes, however, 1000-fold higher concentrations were required (S6D Fig).

Finally, we performed siRNA-mediated knock-down of HCA_3_ in freshly isolated human monocytes (S6E Fig) and determined cAMP levels upon stimulation with 3HO and D-PLA in comparison to scrambled negative control siRNA (siNC)-transfected cells (Fig 4G). In contrast to control (siNC)-transfected monocytes the reduction in intracellular cAMP levels was abolished in si*HCAR3*-transfected monocytes demonstrating HCA_3_ specificity of the D-PLA induced effect (Fig 4G).

## Discussion

A recent paleogenetic study provides a comprehensive summary of existing evidence for large-scale ecological transitions occurring during hominid evolution that caused changes of the habitat accompanied by changes in diet and the microbial environment [8]. The exposure to an altered microbial environment most likely posed a selective pressure and required host adaptation but may also have provided the advantage to access new ecological niches.

In the present study, we provide evidence for such a unique example, where genetic events potentially improved the availability of a new food repertoire under changed ecological conditions that possibly triggered the fixation of a duplicated gene with new functions in hominids.

Through analyses of the evolutionary history of the HCA receptor family, we now show that HCA_3_, being absent in non-hominoid primates and all other vertebrates, resulted from a gene duplication that occurred before the split of gibbons from great apes (Fig 1B). In apes, we found gradual fixation of amino acid positions in HCA_3_ (Fig 1C). We discovered new LAB-derived agonists acting at all ape HCA_3_ orthologs. Both, D-PLA and ILA, activate human HCA_3_ with an about 10-fold higher potency when compared to the endogenous agonist 3HO (Tables 1 and 3). Further, our functional analyses of great ape HCA_3_ orthologs revealed, that potencies of D-PLA are significantly higher in human, chimpanzee and gorilla compared to orangutan (Fig 4C). This led us to do an extensive literature search to identify potential global environmental changes that conceivably coincided with dietary changes and are archaeologically accepted to have occurred when the last common ancestor of human, chimpanzee and gorilla lived on earth. As mentioned at the beginning of the discussion, a recent study showed that about 10 million years, ago a mutation in the class IV alcohol dehydrogenase appeared in the common ancestor that humans share with gorillas and chimpanzees, which allowed an increased tolerance of alcohol [8]. In this study, a scenario was projected in which the large-scale ecological transitions caused the common ancestor to move out of the trees and lead a more terrestrial life [8]. Carrigan et al. conclude that this new life style rendered fruit picking directly from the trees to be less likely but increased the probability that food that had started to ferment was collected from the ground [8]. This is suggested to have posed the selective pressure that increased the tolerance to dietary alcohol before human-directed fermentation [8]. In this context, not only the increased activity of ethanol-metabolizing enzymes (e.g. ADH4) might have provided a selective advantage but also an immune system that can sense high levels of D-PLA ingested with lacto-fermented food (including plants, fish, meats, milk) [35]. Our functional data combined with the described archaeologically documented evolutionary context gives rise to the hypothesis that in the last common ancestor of human, chimpanzee and gorilla, increased ingestion of fermented food was likely associated with increased ingestion of LAB accompanied by increased levels of D-PLA. D-PLA is a metabolite, produced in high concentrations by LAB in the process of lactic fermentation and has a broad antimicrobial activity against bacteria and fungi [35].

We demonstrate, using LC-MS that D-PLA is quickly absorbed from the gastrointestinal tract upon ingestion of LAB-fermented Sauerkraut, can reach physiologically relevant plasma concentrations and is renally eliminated in humans (Fig 4A and 4C). These diet-induced PLA plasma concentrations are sufficient to activate HCA_3_ in human monocytes and potentially modulate human immune functions (Fig 4E–4G).

The beneficial effects of LAB-fermented food on human health are numerous, ranging from protection of the intestinal barrier, limitation of inflammatory cytokine production to improved fasting insulin levels and glucose turnover rates among many others [41]. HCA_3_ is expressed in many different immune cells but also adipose tissue (S7 Table). We hypothesize that the LAB-mediated, HCA_3_-dependent physiological impact likely extends to influences on the human host energy storage. This would also be plausible in an evolutionary context, since it is known that the nutritional and functional properties of food are enhanced by LAB-fermentation due to the transformation of substrates and the formation of bioactive or bioavailable end-products [25].

In summary, our work provides multifaceted evidence supporting the hypothesis that HCA_3_ evolved as a GPCR activated by LAB metabolites. Our data suggests that this might have improved tolerance to the increased ingestion of LAB in the last common ancestor of human, chimpanzee and gorilla. As LAB-derived D-PLA is specifically recognized by HCA_3_ expressed in monocytes (Fig 4H) and numerous studies describe increased hypo-responsiveness of the immune system through anti-inflammatory processes for D-PLA and LAB (S1 Table), we speculate that HCA_3_ plays a role in mediating at least some of those effects (Fig 4H). In the present study, we identified a new set of microbial-derived metabolites acting as classical signaling molecules in the human host through recognition by a specific receptor. The question remains how D-PLA affects monocyte functions. Future studies shall address whether HCA_3_ activation by D-PLA impacts phagocytic capacity of monocytes or influences differentiation of monocytes to macrophages. Does D-PLA, through activation of HCA_3_, prime monocytes to either increase of pro-inflammatory host response to concomitantly ingested pathogenic bacteria or reduction of pro-inflammatory response to LAB? At last, our study opens-up the interesting question of how D-PLA ingested with LAB-fermented food can influence energy storage in HCA_3_-expressing adipocytes.

## Materials and methods

### Ethics statement

The studies on humans and with human materials were conducted in accordance with the Declaration of Helsinki and with the recommendations of “Ethik-Kommission an der Medizinischen Fakultät der Universität Leipzig” with written informed consent from all blood donors. The protocol was approved by the aforementioned committee (313/14-ek).

### *HCAR* ortholog identification

#### Mining of NCBI trace archives

*HCAR* sequences of various mammalian species were obtained from extensive database mining followed by assembly, analysis and manual proof-reading. Trace identifier, NCBI accession or SRA accession numbers are listed in S2 Table. All newly obtained *HCAR* sequences have been deposited in the GenBank database: accession no. KU285431-KU285452, as listed in S2 Table.

#### Amplification, sequencing and cloning of HCAR orthologs from apes and mouse

To analyze the sequence of *HCAR* orthologs, genomic DNA samples were prepared from tissue of various species (sources are given in S8 Table). Tissue samples were digested in lysis buffer (50 mM Tris/HCl, pH 7.5, 100 mM EDTA, 100 mM NaCl, 1% SDS, 0.5 mg/ml proteinase K) and incubated at 55°C for 18 h. DNA was purified by phenol/chloroform extraction and ethanol precipitation. Degenerated primer pairs (S9 Table) were used to amplify *HCAR*-specific sequences. Primer pairs, positioned in the 5’- and 3’- UTR, were designed to simultaneously amplify HCA_2_ and HCA_3_. PCR reactions were performed with Taq and Pfu polymerase under variable annealing and elongation conditions. A standard PCR reaction (50 μl) contained genomic DNA (100 ng) with primers (1.5 μM each), ThermoPol reaction buffer (1x), dNTP (250 μM, each) as well as Taq and Pfu polymerase (1 U, NEB, Frankfurt am Main, Germany). The reactions were initiated with denaturation at 95 °C for 1 min, followed by 35 cycles of denaturation at 95 °C for 30 s, annealing at 55 °C for 30 s and elongation at 72 °C for 1 min. A final extension step was performed at 72 °C for 10 min. Specific PCR products were subcloned into the pCR2.1-TOPO vector (Invitrogen, Paisley, UK) and sent to Seqlab for sequencing. All newly obtained *HCAR* sequences have been deposited in the GenBank database (accession no. KU285431-KU285452, S2 Table). Protein sequence alignments are depicted in S3 Fig.

The full-length *HCAR* were inserted into the mammalian expression vector pcDps [42] and epitope-tagged with an N-terminal hemagglutinin (HA) epitope and a C-terminal FLAG-tag by a PCR-based overlapping fragment approach. Identity of all constructs and correctness of all PCR-derived sequences were confirmed by restriction analysis and sequencing.

### Sequence alignments and PAML analyses

HCA_1_, HCA_2_ and HCA_3_ of 54 selected vertebrate species were aligned to infer the phylogenetic model of *HCAR* evolution as shown in Fig 1 (S1 Fig). Mammalian HCA_1_ and HCA_2_ were aligned to infer a phylogenetic tree of 88 mammalian species. All nucleotide alignments were generated with the ClustalW algorithm (Bioedit Sequence Alignment Editor 7.0.9 [43]) followed by manual trimming where gaps were deleted. Phylogenetic evolutionary history was inferred by the Maximum Likelihood method based on the General Time Reversible model using MEGA6 [44]. The resulting tree (S2 Fig) with a 75% cut-off value was used for PAML analyses. Tests of selection (ω = d_N_/d_S_) were accomplished by maximum likelihood using a codon-based substitution model implemented in PAML version 4.2 [11]. Branch models [45] that allow ω to vary among branches in the phylogeny were applied to determine ω ratios along particular lineages. Likelihood ratio tests (LRT) were performed to test nested competing hypotheses (S3 Table).

### Cell culture and functional assays

CHO-K1 cells were grown in Dulbecco’s Modified Eagle Medium: Nutrient Mixture F-12 (DMEM/F12) supplemented with 10% fetal bovine serum (FBS), 100 U/ml penicillin and 100 μg/ml streptomycin. Cells were maintained at 37 °C in a humidified 5% CO_2_ incubator. For transient transfection Lipofectamine 2000 (Life Technologies, Darmstadt, Germany) was used. Cells were split into 25 cm^2^-cell culture flasks (0.9 x 10^6^ cells/flask) and transfected with a total amount of 3 μg of plasmid the following day.

#### ALPHAScreen cAMP assay

cAMP content of cell extracts was determined by a non-radioactive assay based on the ALPHAScreen technology according to the manufacturers’ protocol (Perkin Elmer LAS, Rodgau-Jügesheim, Germany) [46]. One day after transfection cells were split into 96-well plates (2 x 10^4^ cells/well). Stimulation with various agonist concentrations (all compounds from Sigma-Aldrich, Seelze, Germany) or diluted human urine and plasma samples was performed 48 h after transfection. Reactions were stopped by aspiration of media and cells were lysed in 20 μl of lysis buffer containing 1 mM 3-isobutyl-1-methylxanthine (IBMX). From each well 5 μl of lysate were transferred to a 384-well plate. Acceptor beads and donor beads were added according to the manufacturers’ protocol. Cyclic AMP accumulation data were analyzed using GraphPad Prism version 6.03 (GraphPad Software, San Diego California USA).

#### Dynamic mass redistribution assay

To measure label-free receptor activation, a dynamic mass redistribution (DMR) assay (Corning Epic Biosensor Measurements) with CHO-K1 cells transiently expressing *HCAR* orthologs was performed. One day after transfection, cells were detached using Versene solution and transferred into a fibronectin-coated 384-well Epic microplate (1.2 x 10^4^cells per well) and cultured for 24 h to reach confluent monolayers. After 2 h of equilibration in HBSS buffer with 20 mM HEPES (pH 7.4), incubation with various compound concentrations was performed and DMR was recorded for 60 min.

#### ELISA

Cell surface expression of N-terminal HA-tagged receptor constructs was determined using an indirect cellular ELISA and total receptor expression of full-length HA/FLAG double-tagged HCA constructs was assessed using a “sandwich-ELISA” both described in [46].

### Manual reanalysis and comparison to FPKM values of a publicly available RNA-Sequencing dataset

All 134 RNA-sequencing samples belonging to Bioproject: PRJNA326727, (GEO Accession: GSE83687) [47] were manually reanalyzed and compared to available FPKM values as stated in S6 Table and S7 Fig.

### Mining of human microbiome data for genes of enzymes with ability to produce D PLA

Two large collections of metagenomic data from the human gut microbiome were searched for D-lactate dehydrogenase (EC 1.1.1.28), the enzyme that catalyzes the conversion of D-lactate to pyruvate, but has also been shown for some LAB to convert phenylpyruvate to D-PLA. Metagenomic data from 1,885 human stool samples from the curated Metagenomic Dataset (https://github.com/waldronlab/ curatedMetagenomicData) [48] were searched for 1,443 EC 1.1.1.28-related UniRef100 entries. In addition, genes with the K03778 annotation were searched in the Integrated Gene Catalogue (IGC) [49] reference data set for the human gut microbiome, which comprises data from just over 1,000 individuals. Detailed information, a representative tree of the detected genes and a list of all entries are summarized in the Supplementary Information (S5 Fig, S4 Table).

### Peripheral blood mononuclear cells (PBMCs) and monocytes

#### Monocyte isolation

PBMCs were obtained by Ficoll-Paque (GE Healthcare) density gradient centrifugation. After repeated washing in PBS containing 0.3 mM EDTA, untouched monocytes were isolated by negative magnetic depletion using the human Monocyte Isolation Kit II (Miltenyi Biotec) according to the manufacturer’s protocol. The cell preparations were >95% monocytes as determined by morphology and flow cytometry.

#### Monocyte migration

For migration analysis, ThinCert cell culture inserts (Greiner) with 8 μM pores were placed in a 24-well cell culture plate. To each well of the cell culture plate 1,000 μl of HBSS buffer supplemented with 20 mM HEPES (pH 7.4) and 1% FBS with or without chemoattractant were added. Pelleted freshly isolated monocytes in PBS with 0.3 mM EDTA and 1% FBS were labeled for 30 min at 4°C with 2 μM calcein AM (Thermo Scientific). Subsequently monocytes were washed and resuspended in HBSS buffer with 20 mM HEPES (pH 7.4) and 1% FBS to a final concentration of 5 × 10^5^ monocytes per ml. 200 μl of the monocyte suspension were added to each insert or for control purposes directly to the bottom well and incubated at 37°C and 5% CO_2_. After 1 h the inserts were discarded, plates spun for 5 min at 700 rpm and imaged as well as analyzed using the Celigo S Imaging Cytometer (Nexcelom).

#### Monocyte siRNA transfection

Freshly isolated human monocytes were transfected with siRNA specifically targeting *HCAR3* (OriGene), as shown in Stäubert et al. (2015) [50], using Viromer Green (Lipocalyx) according to the manufacturer’s protocol. 10 μL of siRNA-Viromer mix were preplated into a 96-well plate before the addition of freshly isolated monocytes resuspended in RPMI supplemented with 10% FBS (1.5 x 10^5^ monocytes/well) which were then incubated 14 h at 37 °C in a humidified 5% CO_2_ incubator. cAMP assays were performed as described below. To monitor the transfection efficiency a red fluorescent siRNA (OriGene) and Hoechst 33342 (Sigma-Aldrich) were used. The viability of transfected monocytes was determined by staining with propidium iodide (PI) (Thermo Fisher Scientific) and Hoechst 33342. Transfection efficiency and viability were analyzed using the Celigo S Imaging Cytometer (Nexcelom). The transfection efficiency was found to be ≥ 80% with less than 50% of monocytes being PI-positive (S6 Fig).

#### Monocyte cAMP assay

Freshly isolated monocytes or PBMCs were resuspended in HBSS buffer with 20 mM HEPES (pH 7.4) and 1 mM IBMX, seeded in 96-well plates (1 x 10^5^ monocytes/well, 2 x 10^5^ PBMCs/well) with or without PTX (100 ng/ml) and incubated for 1 h at 37 °C. Stimulation with or without agonists was performed in presence of 10 μM forskolin for 1 h at 37 °C. Measurement of accumulated cAMP was performed as described above.

### Determination of PLA in human plasma and urine

100 mg of D-PLA were dissolved in 30 ml H_2_O and ingested by a healthy male subject (78 kg, 1.80 m). 2.5 ml of EDTA blood samples were taken right before and 30 min, 60 min, and then every hour up to 7 h after ingestion. In a subsequent, chronically separate experiment the same healthy male subject adapted to a lacto-vegetarian diet, including uptake of 500 g per day of fresh (non-pasteurized) Sauerkraut (Spreewaldhof, Germany) for three days. 2.5 mL of EDTA blood samples were taken before diet, 2 h postprandial over three days and 5 days after having stopped the above described diet. Further, urine samples were taken over the time course. Similarly, three healthy female subjects ingested 5–6 g of fresh (non-pasteurized) Sauerkraut per kg body weight for one day. 2.5 mL of EDTA blood samples and urine samples were taken before ingestion and 2 h postprandial. Fresh whole blood samples were centrifuged for 10 min at 4 °C and 800xg to isolate plasma, which was stored in 500 μl aliquots at -80 °C until further analyses. Urine was collected and the total volume was determined. 10 μl of human plasma and urine were treated with 90 μl precipitating agent (acetonitrile including the internal standard phenyl-d5-lactate at 10 ng/ml), thoroughly mixed and centrifuged for 5 min at 13,000xg. Supernatants were transferred to autosampler vials and 10 μl were injected into the Liquid Chromatography Mass Spectrometry (LC-MS) system. It consisted of a Prominence UFLC system from Shimadzu (Duisburg, Germany) and a QTRAP 6500 from SCIEX (Framingham, MA, USA). Chromatographic separation took place on a Luna HILIC column (3 μm, 50 x 2 mm) from Phenomenex (Darmstadt, Germany) via gradient elution at a flow rate of 0.4 ml/min. The mobile phase was 15 mmol/l ammonium acetate buffer (pH 6) in acetonitrile and water. Electrospray ionization was applied in negative mode. Mass transitions were m/z 165>103 and m/z 170>108 for phenyl-lactate and phenyl-d5-lactate, respectively. Quantitation was carried out using a semi-quantitative approach following Eq 1 with c and A as the concentration and the peak area of the analyte (An) and the internal standard (IS). The response factor (RF) was determined from signal responses in standard solutions and equaled 1.

$$c_{An}=\frac{c_{IS}*A_{An}}{A_{IS}}*RF$$ (1)

## Supporting information

S1 Fig Phylogenetic tree of HCA_1_, HCA_2_ and HCA_3_ for 54 selected species to infer the model shown in Fig 1B. (TIF)

S2 Fig Phylogenetic tree of 88 mammalian species used for HCA_1_ and HCA_2_ PAML analyses. (TIF)

S3 Fig Amino acid sequence alignment of all functionally tested HCA orthologs. (TIF)

S4 Fig Human HCA_3_ but not HCA_1_ or HCA_2_ shows a DMR response upon stimulation with D-Phe, D-Trp and 3HDec and comparison of potencies of different agonists acting at ape HCA_3_ orthologs. (TIF)

S5 Fig Phylogenetic tree of D-lactate dehydrogenase-related genes and metagenomic proportion of EC 1.1.1.38. (TIF)

S6 Fig Plasma and urine PLA levels, reduction in intracellular cAMP levels of freshly isolated human peripheral blood mononuclear cells (PBMCs) and monocytes upon stimulation with HCA_3_ agonists and siRNA transfection efficiency of human monocytes. (TIF)

S7 Fig Comparison of manually curated RNA-sequencing data (A) versus reported Fragments Per Kilobase Million (FPKM) values (B) for *HCAR2* and *HCAR3* and Transcripts Per Kilobase Million (TPM) values for tissues/organs/cell types as deposited in Expression Atlas (https://www.ebi.ac.uk/gxa/home). (TIF)

S1 Table D-PLA sources as described in literature. (PDF)

S2 Table NCBI database accession numbers and sequence description of one representative *HCAR1* ortholog of *Chondrichthyes*, *Actinopterygii*, *Coelacanths* and *Amphibia* and mammalian *HCAR* orthologs. (PDF)

S3 Table Maximum likelihood estimates of d_N_/d_S_ ratios (ω) for 88 mammalian HCA_1_ and HCA_2_ orthologs, for 5 ape HCA_1_, HCA_2_ and HCA_3_ orthologs and for 5 Hylobatidae HCA_1_ and HCA_2_ orthologs under different branch-specific models using PAML. (PDF)

S4 Table Bacterial species carrying genes for EC 1.1.1.28 that were detected in human gut microbiota of the curated Metagenomic Dataset. (PDF)

S5 Table Testing of PLA-containing human urine and plasma samples on transiently with human HCA_2_ or HCA_3_ or empty vector transfected CHO-K1 cells. (PDF)

S6 Table Comparison of manually curated RNA-Sequencing data versus reported FPKM for *HCAR2* and *HCAR3*. (PDF)

S7 Table TPM values as downloaded from https://www.ebi.ac.uk/gxa/home. (PDF)

S8 Table Sources of genomic DNA used for *HCAR* amplification. (PDF)

S9 Table Primers used for HCA_1_, HCA_2_ and HCA_3_ ortholog amplification, sequencing and introduction of epitope tags. (PDF)

S1 References All references cited in the supporting information are summarized in this file. (PDF)
