# Correction: Metabolites of lactic acid bacteria present in fermented foods are highly potent agonists of human hydroxycarboxylic acid receptor 3

**DOI:** 10.1371/journal.pgen.1008283

**Published:** 2019-07-19

**Authors:** Anna Peters, Petra Krumbholz, Elisabeth Jäger, Anna Heintz-Buschart, Mehmet Volkan Çakir, Sven Rothemund, Alexander Gaudl, Uta Ceglarek, Torsten Schöneberg, Claudia Stäubert

Fig 4E is identical to Fig 4G. The authors have provided a corrected Fig 4 here, which shows the data as described in the figure legend.

**Fig 4:**
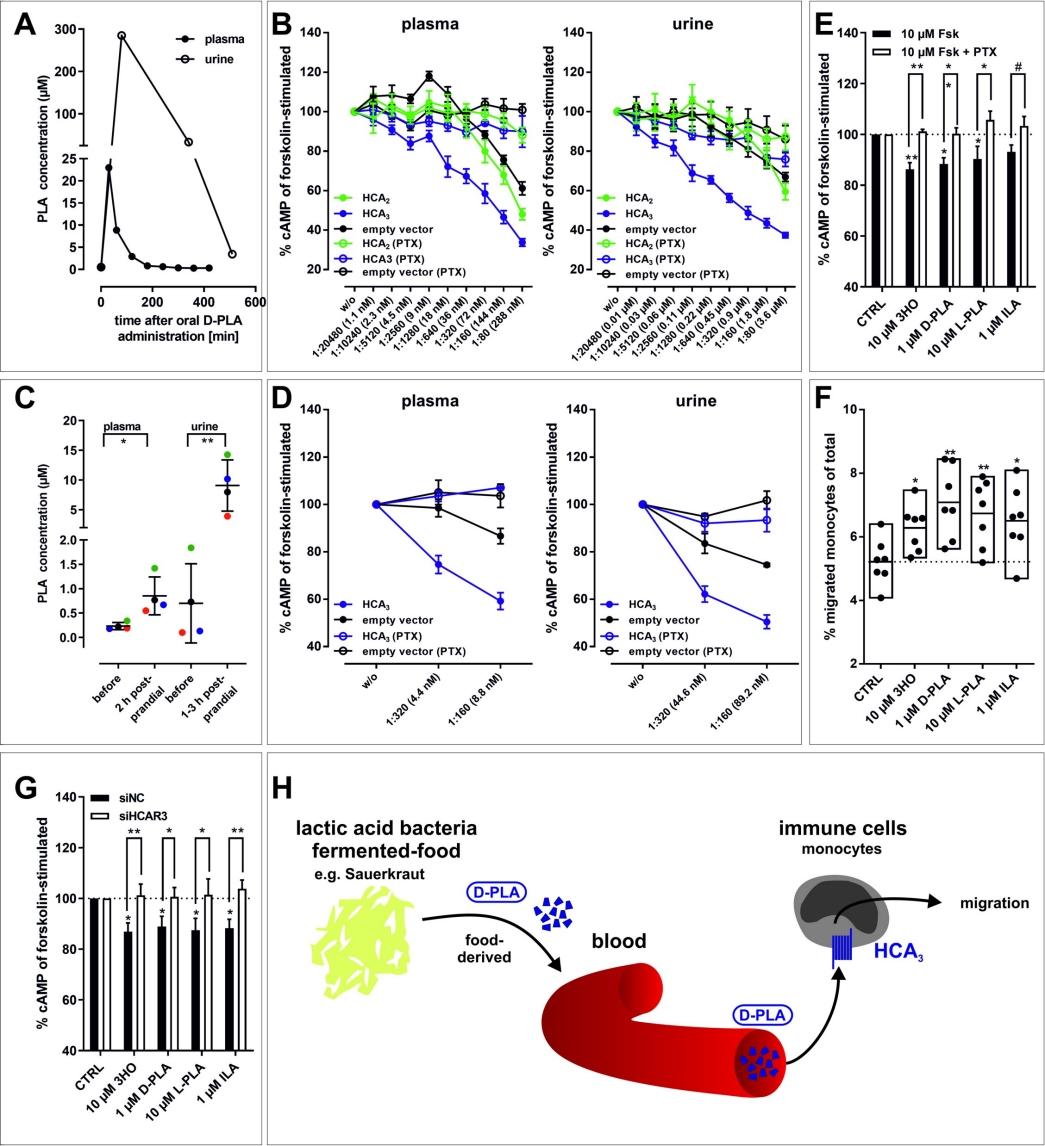
D-PLA is absorbed from the human gut, reaches μM plasma concentrations and activates HCA_3_ in human monocytes. (A) Upon oral ingestion of 100 mg D-PLA (one individual) plasma and urine PLA levels were measured using LC-MS. Details about experimental setup are stated in *Materials and Methods*, section *Determination of PLA in human plasma and urine*. (B) PLA containing plasma (23 μM, corresponding to the 30 min time point from (A)) and urine (285 μM, corresponding to the 80 min time point from (A)) were stepwise 1:2-diluted and tested in cAMP inhibition assays. (C) Upon oral uptake of 5–6 g Sauerkraut per kg body weight (n = 4 individuals, each individual labeled with a different color), plasma and urine PLA levels were measured using LC-MS. Details about experimental setup are stated in *Materials and Methods*, section *Determination of PLA in human plasma and urine*. (D) PLA containing plasma (1.4 μM, 2 h postprandial) and urine (14.3 μM, 3 h postprandial) were stepwise 1:2-diluted and tested in cAMP inhibition assays. (B, D) Data is shown as fold over unstimulated cAMP level, given as mean ± SEM (n = 3) and is summarized in S5 Table. (E) 3HO, D-PLA, L-PLA, and ILA induced a pertussis-toxin (PTX) sensitive reduction in cAMP levels and (F) migratory responses in human monocytes. (E) Data is given as percent of cAMP level in monocytes stimulated with 10 μM forskolin without agonist. The mean ± SEM (n = 3 different donors) is shown. (F) Data is shown as percent migrated monocytes of total monocytes (min to max bars with line at mean, n = 7 different donors). (G) siRNA-mediated knock-down of HCA_3_ in human monocytes diminished the agonist-induced reduction of cAMP levels still present in negative control siRNA (siNC) transfected monocytes. Data is shown as mean ± SEM (n = 7 different donors). (H) D-PLA is absorbed from ingested LAB-fermented food (Sauerkraut) and induces HCA_3_-dependent migration in human monocytes. This opens up new perspectives to study the role of HCA_3_ activation by LAB-derived metabolites in both immune function and energy homeostasis. (E, G) Paired two-tailed t-tests were performed to analyze the effect of PTX and siRNA transfection. (E, F, G) Statistical analyses were performed using an ordinary One-Way ANOVA (Dunnett’s multiple comparisons test) testing against control (CTRL, vehicle EtOH). ^#^ ≤ 0.1, * P ≤ 0.05; ** P ≤ 0.01; *** P ≤ 0.001.
