# Supplementary figures and images for: Metabolites of lactic acid bacteria present in fermented foods are highly potent agonists of human hydroxycarboxylic acid receptor 3
Source: PLoS Genet. 2019 May 23;15(5):e1008145. doi: 10.1371/journal.pgen.1008145 (PMC6532841; doi:10.1371/journal.pgen.1008145)

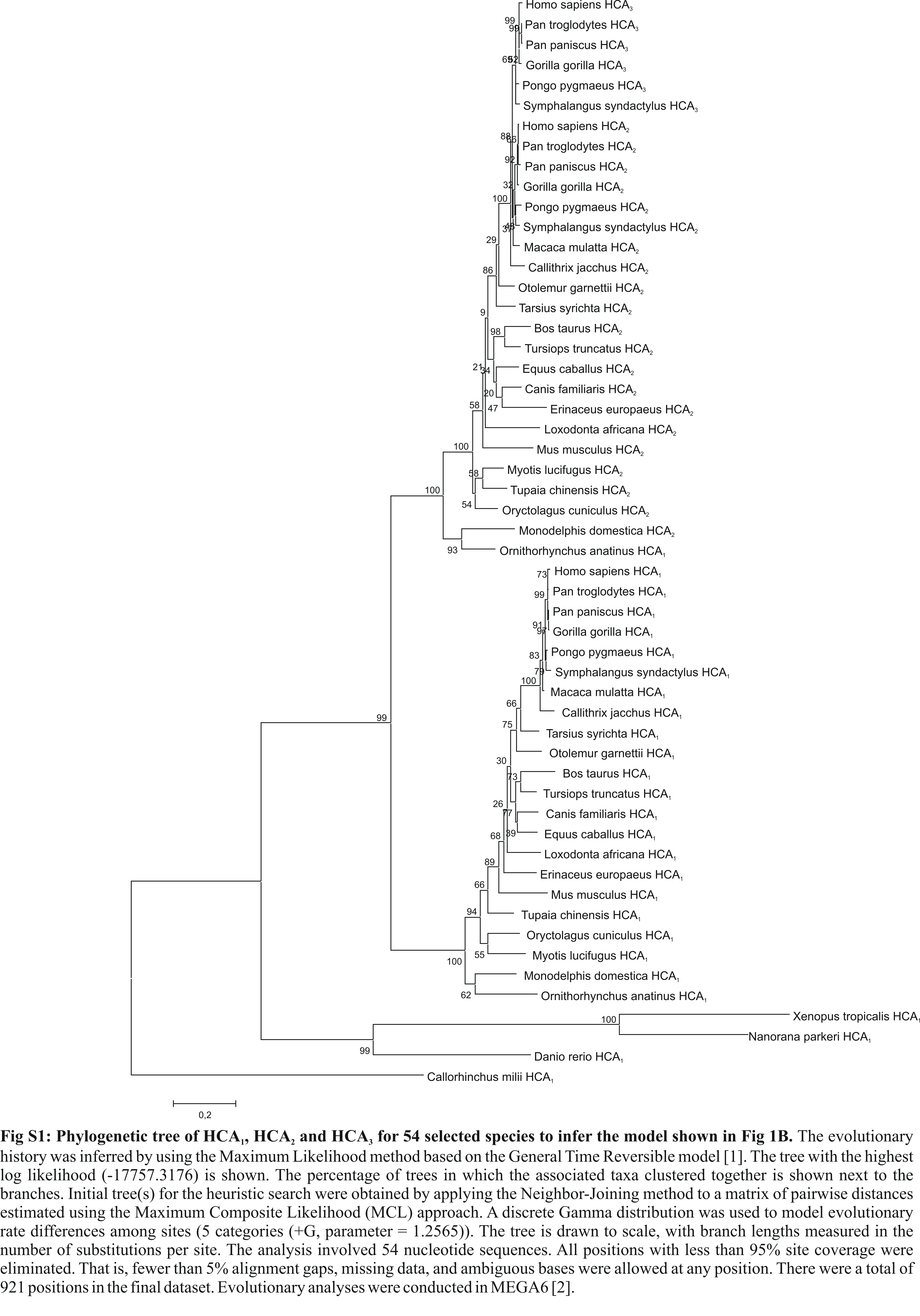

Supplement: S1 Fig — (TIF) [file pgen.1008145.s001.tif]

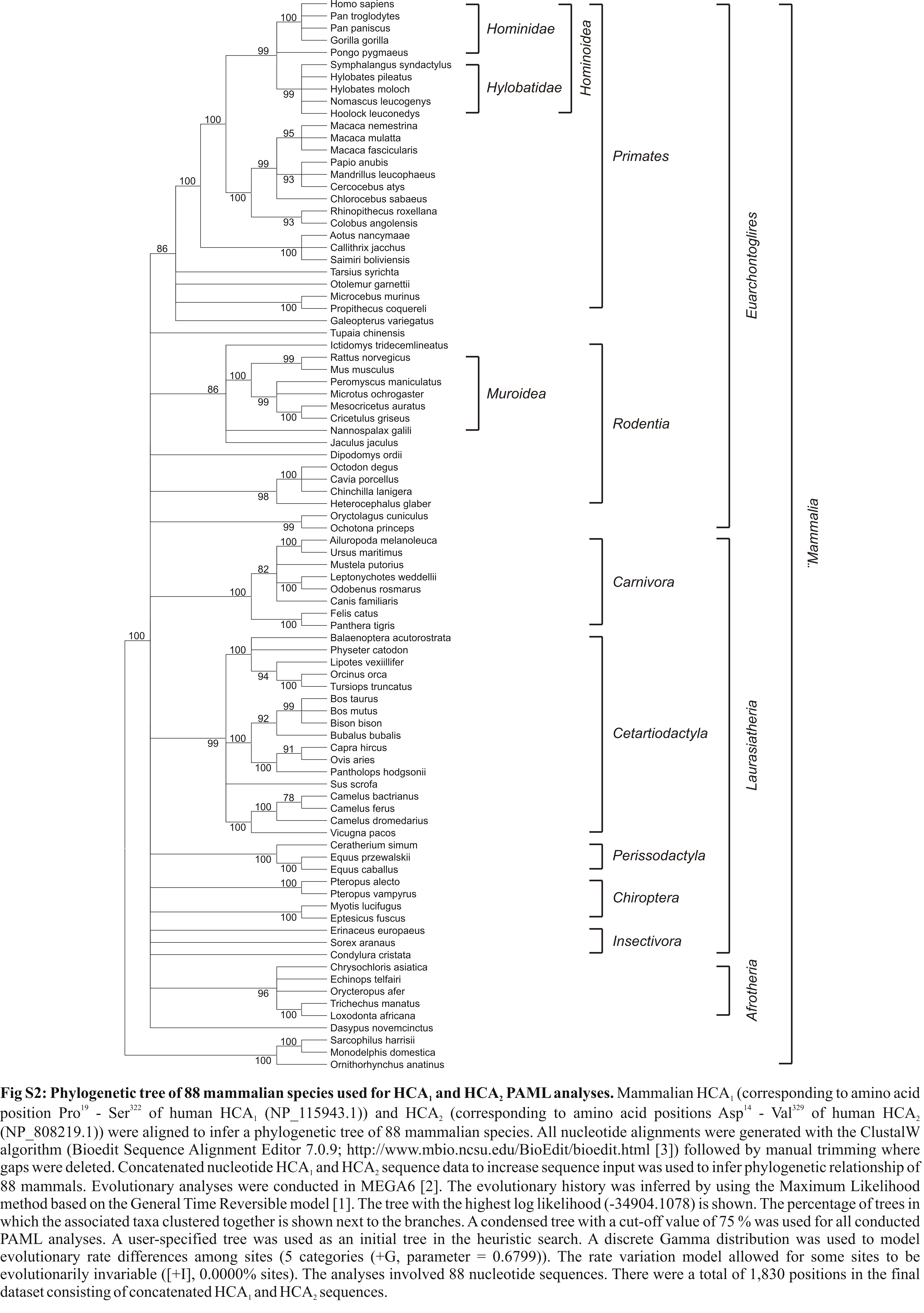

Supplement: S2 Fig — (TIF) [file pgen.1008145.s002.tif]

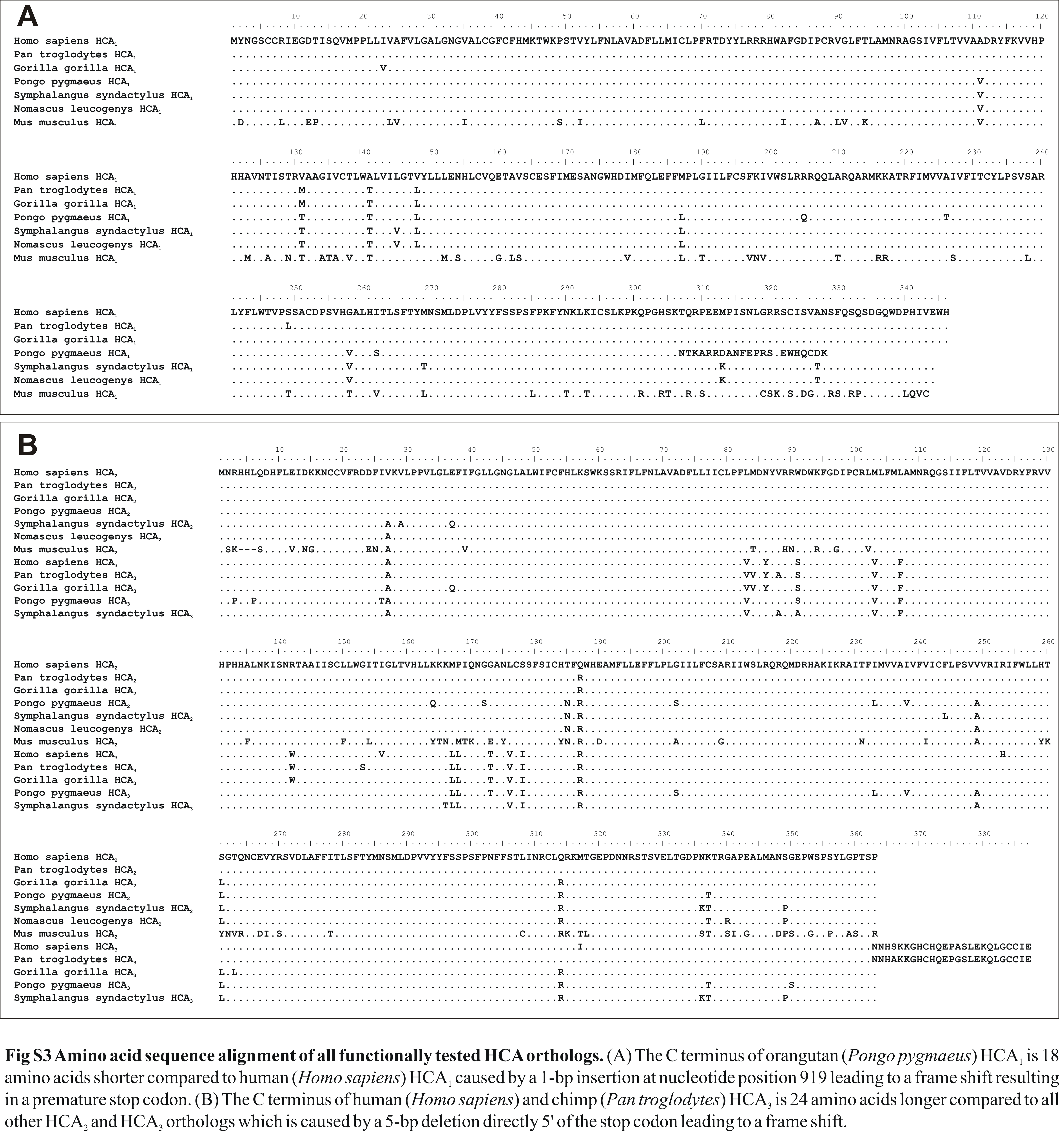

Supplement: S3 Fig — (TIF) [file pgen.1008145.s003.tif]

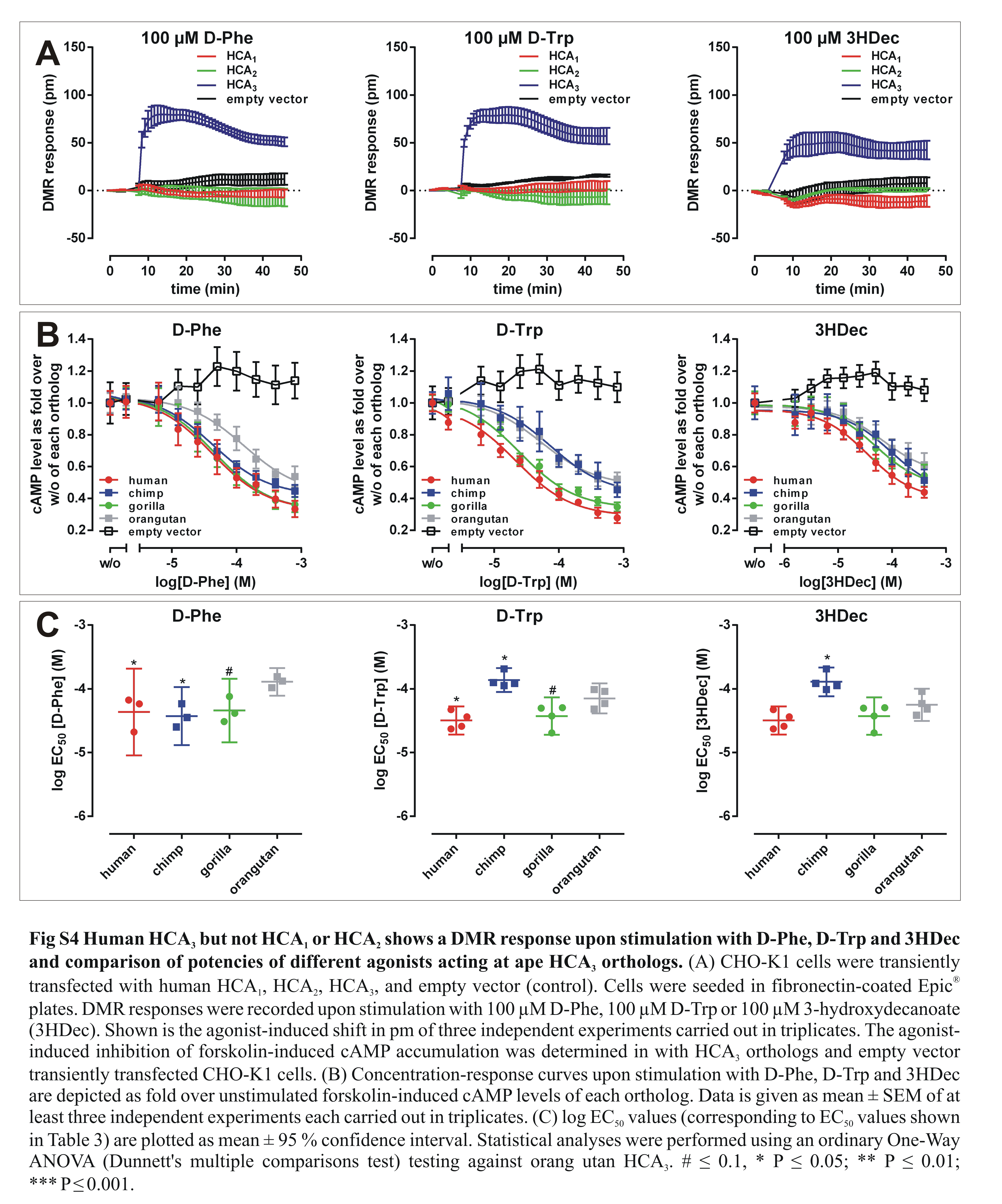

Supplement: S4 Fig — (TIF) [file pgen.1008145.s004.tif]

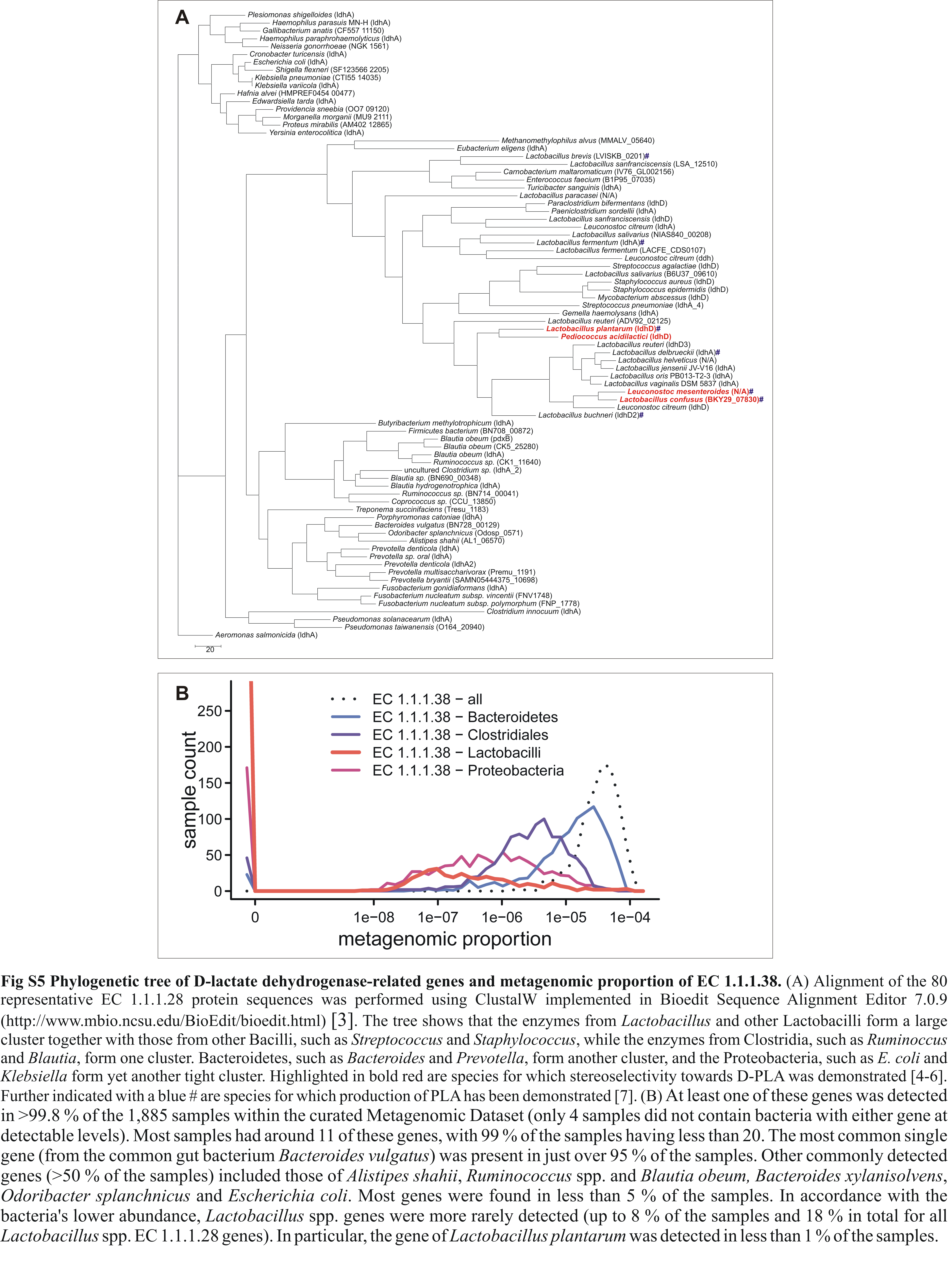

Supplement: S5 Fig — (TIF) [file pgen.1008145.s005.tif]

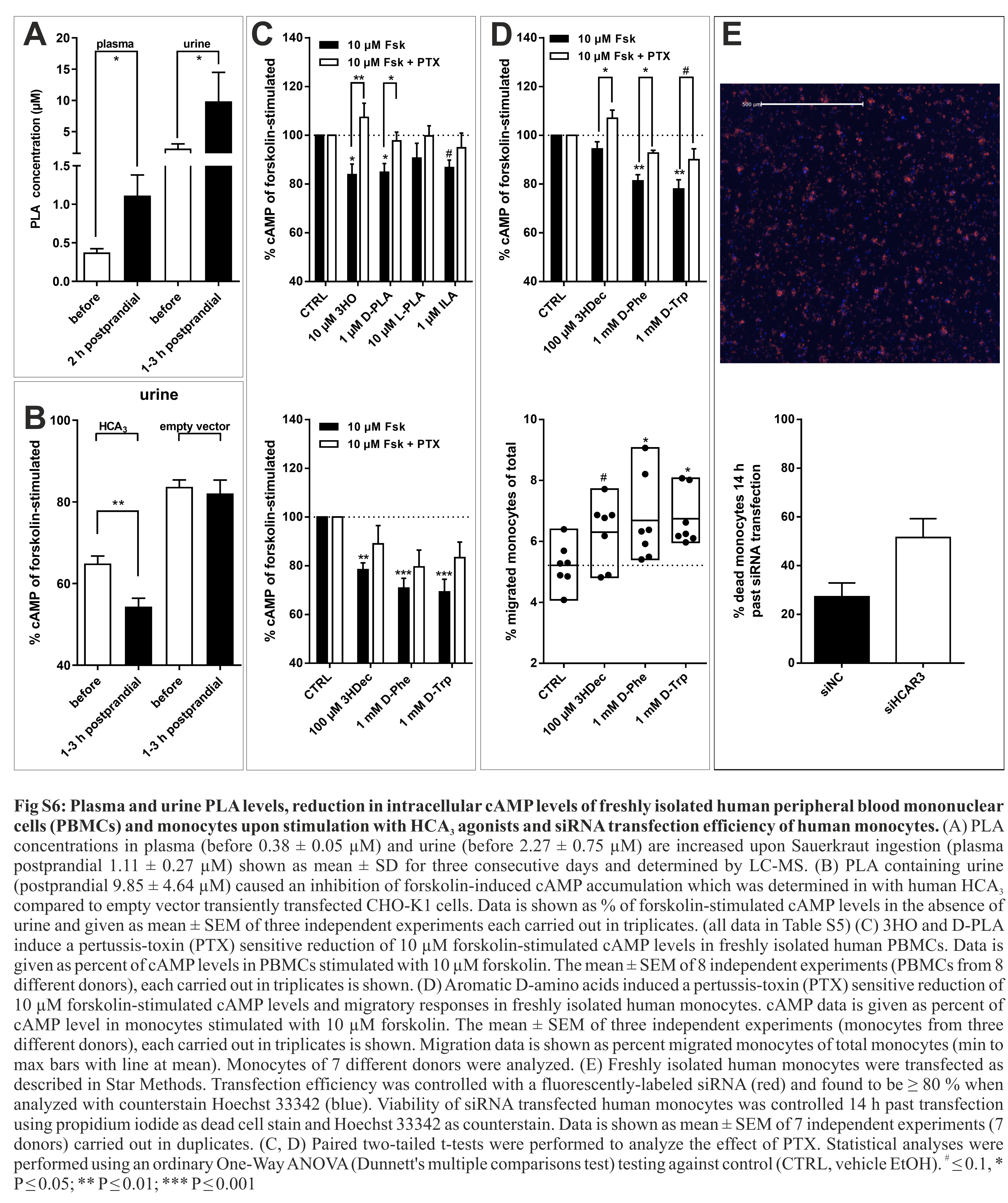

Supplement: S6 Fig — (TIF) [file pgen.1008145.s006.tif]

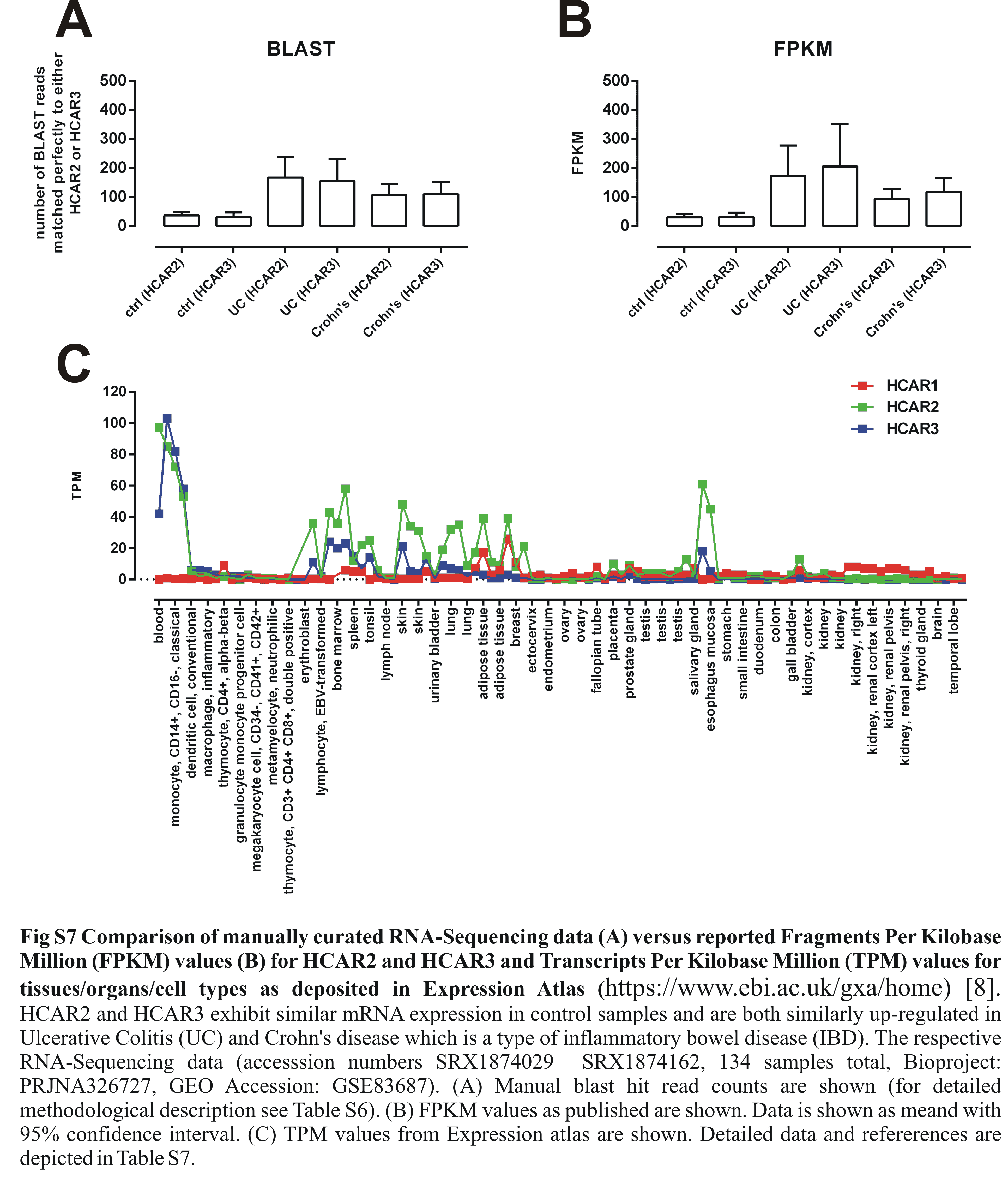

Supplement: S7 Fig — (TIF) [file pgen.1008145.s007.tif]
