# Supplementary material for: Metabolites of lactic acid bacteria present in fermented foods are highly potent agonists of human hydroxycarboxylic acid receptor 3
Source: PLoS Genet. 2019 May 23;15(5):e1008145. doi: 10.1371/journal.pgen.1008145 (PMC6532841; doi:10.1371/journal.pgen.1008145)
Supplement: S1 Table — (PDF) [file pgen.1008145.s008.pdf]

Supplementary Table S1

D-PLA sources as described in literature

Source/Origin	PLA-concentration	bacterial strain	physiological effects/associations	reference
Sourdough, human (ATCC4356), raw poultry meat	30 - 200 µM (ATCC4356)	*Lactobacillus acidophilus* [a]	Stimulation of immunity, facilitating anti-inflammatory effects and innate immunity-mediated inactivation of tumor cells [9]<br>shortened duration of diarrhea and potential therapy for acute rotaviral gastroenteritis [10]	[7, 11, 12]
Sourdough, fish products	160 - 370 µM	*Lactobacillus alimentarius*		[11]
Sauerkraut, Sourdough, Human (ATCC14869)	220 - 460 µM (ATCC14869)	*Lactobacillus brevis* (ATCC14869) [a]	Alleviation of abdominal pain in IBS, anti-inflammatory effects in periodontal disease [13-15]	[11, 16, 17]
		Lactobacillus confusus [a]		[7]
Grass		*Lactobacillus coryneformis* [a]		[7, 18]
Human	211 - 241 µM (35 - 40 µg/mL)	*Lactobacillus fermentum* [a]	Increased antioxidative activity, improved post-prandial lipid status, anti-atherogenic effects, protection against inflammation [19, 20]<br>part of dominant pool of the intestinal microbiota of a healthy human	[21, 22]
Raw Milk		*Lactobacillus paracasei subsp. paracasei* SM20 [a], *Lactobacillus. jensenii* SM11[a]	Increased IgA, anti-inflammatory IL-10 and pro-inflammatory IFNγ producing cells in small intestine	[23]

			Higher systemic levels of anti-inflammatory cytokines [10]	
Human (vagina)		*Lactobacillus pentosus* [a]		[24, 25]
Sauerkraut, Sourdough, plants, grass silage, orange juice	270 - 340 µM	*Lactobacillus plantarum* [a]	Induction of genes associated with anti-inflammatory activites and immune tolerance, positive regulators of proliferation and pathways modulating metabolic function [26]<br>Reduced body weight gain, fat accumulation, lowered plasma insulin, leptin, total-cholesterol and liver toxicity biomarkers [27]<br>influences intestinal and systemic immunity[28]<br>increased the relocation of occludin and ZO-1 into the tight junction area between duodenal epithelial cells. [29]	[11, 12, 16, 17, 24, 30-35]
Cereal environment, cheese, malted barley		*Lactobacillus reuteri*	Reduced mean duration of diarrhea[36]<br>Inhibition of TNF production by LPS activated human monocytes<br>Immunosuppressive vs. immunostimulatory action depends on specific strain [37]	[12]
Human	170 - 230 µM	*Lactobacillus rhamnosus*	Improved immune response to viruses, promoting cell survival in epithelial cells, anti-inflammatory responses and modulation of Th1/Th2 balance, positively effects integrity of barrier function [9]	[11]

Sourdough	220 - 350 µM	*Lactobacillus sanfranciscensis*	Reduction of pro-inflammatory cytokines, weight loss and gut permeability in a chronic colitis mouse model [38]	[11]
Sourdough, rice cakes	260 - 430 µM	*Leuconostoc citreum* [a]		[11, 12]
raw smoked sausage		*Leuconostoc lactis*		[7, 12]
Sauerkraut, Sourdough, Olive phylloplane, Fermenting olives	90 µM;<br>570 µM;<br>100 µM (D-PLA)	*Leuconostoc mesenteroides subsp. mesenteroides* (ITM12K; ITMY30; ATCC8293) [b]	Anti-inflammatory action, induction of pro-apoptotic and anti-proliferative effects in colon cancer cells, antioxidant properties, improves IBD in mouse model [39, 40]	[6, 11, 16, 17, 35, 41]
Cheese	90 µM	*Enterococcus faecium* ATCC882	Antioxidant properties, reduction of pro-inflammatory cytokines in cell culture models, reversion/reduction of stress and inflammatory responses and negative effects on epithelial integrity triggered by pathogens [42, 43]	[11]
Human	812 - 842 µM<br>(135 - 140 µg/mL)	*Eubacterium lentum*	part of dominant pool of the intestinal microbiota of a healthy human	[21]
Human	451 - 511 µM<br>(75 - 85 µg/mL)	*Bifidobacterium bifidum*	Reduction of TNF secretion by LPS stimulated human PBMCs mediated by exo-metabolites [44]<br>part of dominant pool of the intestinal microbiota of a healthy human	[21]
Human	1.2 µM<br>(0.2 µg/mL)	*Bacteroides thetaiotaomicron*		[21]
Human	10.2 µM<br>(1.7 µg/mL)	*Clostridium perfringens*		[21]

Human	5.4 µM (0.9 µg/mL)	*Clostridium sporogenes*		[21]
	1.5 µM (0.25 µg/mL)	*Enterococcus faecalis*	pathogenic facultativ aerobe	[21]
	9.6 µM (1.6 µg/mL)	*Escherichia coli*	pathogenic facultativ aerobe	[21]
	> 12 µM (> 2 µg/mL)	*Klebsiella pneumoniae*	pathogenic facultativ aerobe	[21]
	0.6 µM (0.1 µg/mL)	*Serratia marcescens*	pathogenic facultativ aerobe	[21]
	5.1 µM (0.85 µg/mL)	*Staphylococcus aureus*	pathogenic facultativ aerobe	[21]
	1.5 µM (0.25 µg/mL)	*Staphylococcus epidermidis*	pathogenic facultativ aerobe	[21]

[a] known to produce the D-enantiomer. [b] D-PLA (ATCC8293). Further, Di Cagno et al. provide a comprehensive overview of LAB species that were isolated from raw or spontaneously fermented vegetables and fruits, their functional activities and examples of emerging and traditional fermented vegetables and fruits, indicating the main LAB involved [45].
