# Supplementary material for: Metabolites of lactic acid bacteria present in fermented foods are highly potent agonists of human hydroxycarboxylic acid receptor 3
Source: PLoS Genet. 2019 May 23;15(5):e1008145. doi: 10.1371/journal.pgen.1008145 (PMC6532841; doi:10.1371/journal.pgen.1008145)
Supplement: S2 Table — (PDF) [file pgen.1008145.s009.pdf]

Supplementary Table S2

NCBI database accession numbers and sequence description of one representative HCA_1 ortholog of *Chondrichthyes*, *Actinopterygii*, *Coelacanths* and *Amphibia* and mammalian HCAR orthologs

Latin species name	***English species name***	***NCBI or ENSEMBL accession number / trace identifiers of NCBI trace archive***		
		HCA_1	HCA_2	HCA_3
Chondrichthyes				
Callorhinchus milii	elephant shark	XM_007902210.1		
Rhincodon typus	whale shark	XM_020512881.1		
Actinopterygii				
Astyanax mexicanus	Mexican tetra	XM_007250281.2		
Austrofundulus limnaeus		XM_014023514.1		
Boleophthalmus pectinirostris	great blue-spotted mudskipper	XM_020921815.1		
Clupea harengus	Atlantic herring	XM_012827120.1		
Cynoglossus semilaevis	tongue sole	XM_008325648.2		
Cyprinodon variegatus	sheepshead minnow	XM_015376811.1		
Cyprinus carpio	common carp	XM_019086953.1		
Danio rerio	zebrafish	NM_001163292.1		
Esox lucius	northern pike	XM_010877437.2		
Fundulus heteroclitus	mummichog	XM_012869599.2		
Gasterosteus aculeatus	three-spined stickleback	trace archives gi: 902823148, 869593848, 849397327, 867304063, 867604810, 867234216, 855926790, 866492951, 867476020, 867514201, 867306594, 868023256, 863913155, 866753566, 868977227, 855931071, 864224968, 867300398, 831830686, 866340179)		
Haplochromis burtoni	Burton's mouthbrooder	XM_005946323.2		
Ictalurus punctatus	channel catfish	XM_017477394.1		
Kryptolebias marmoratus	mangrove rivulus	XM_017440390.1		
Labrus bergylta	ballan wrasse	XM_020655416.1		

Larimichthys crocea	large yellow croaker	XM_019278680.1
Lates calcarifer	barramundi perch	XM_018696932.1
Lepisosteus oculatus	spotted gar	XM_015366037.1
Maylandia zebra	zebra mbuna	XM_004566929.3
Monopterus albus	swamp eel	XM_020588126.1
Neolamprologus brichardi	lyretail cichlid	XM_006804703.1
Nothobranchius furzeri	turquoise killifish	XM_015943374.1
Oncorhynchus kisutch	coho salmon	XM_020484975.1
Oncorhynchus mykiss	rainbow trout	XM_021622844.1
Oreochromis niloticus	Nile tilapia	XM_005459638.3
Oryzias latipes	Japanese medaka	XM_004074627.3
Poecilia formosa	Amazon molly	XM_007570857.2
Poecilia latipinna	sailfin molly	XM_015044560.1
Poecilia mexicana	shortfin molly	XM_014999463.1
Poecilia reticulata	guppy	XM_008423323.2
Pundamilia nyererei	Nyerere's Victoria Cichlid	XM_005720042.1
Pygocentrus nattereri	red-bellied piranha	XM_017681657.1
Salmo salar	Atlantic salmon	XM_014138487.1
Scleropages formosus	Asian bonytongue	XM_018746830.1
Sinocyclocheilus anshuiensis		XM_016443409.1
Sinocyclocheilus grahami	Golden-line barbel	XM_016237071.1
Sinocyclocheilus rhinocerous	horned Golden-line barbel	XM_016530661.1
Stegastes partitus	bicolor damselfish	XM_008305231.1
Takifugu rubripes	torafugu	XM_003965407.2
Tetraodon nigroviridis	spotted green pufferfish	trace archives gi: 1144280996, 1144209422, 1145624449, 1143498005, 1143979005, 97629832, 99571522, 1141784461, 1144389438, 99115439, 1145624448, 95652118, 1143979227, 99054120, 1143718859, 1143981004
Xiphophorus maculatus	southern platyfish	XM_005802492.1
Coelacanths		

Latimeria chalumnae	coelacanth	XM_005989018.1	
Amphibia			
Nanorana parkeri	Xizang plateau frog	XM_018556801.1	
Xenopus laevis	African clawed frog	XM_018226771.1	
Xenopus tropicalis	tropical clawed frog	XM_012968999.2	
Mammalia			
Ailuropoda melanoleuca	Giant panda	XM_011226294.1	XM_002913130.2
Aotus nancymaae	Ma's night monkey	XM_012447105.1	
Balaenoptera acutorostrata scammoni	Minke whale	XM_007189590.1	
Bison bison	American bison	XM_010841312.1	
Bos grunniens mutus	Yak	XM_005911827.1	
Bos taurus	Cattle	NM_001145234.1	
Bubalus bubalis	Water buffalo	XM_006047196.1	
Callithrix jacchus	Common marmoset	XM_002753127.2	
Camelus bactrianus	Bactrian camel	XM_010969698.1	
Camelus dromedarius	Arabian camel	XM_010995910.1	
Camelus ferus	Wild Bactrian camel	XM_006195843.1	
Canis lupus familiaris	Dog	NM_001145231.1	
Capra hircus	Goat	XM_005709563.1	
Cavia porcellus	Guinea pig	trace archives gi:<br>1582747945<br>1627289015<br>1645142123<br>1609973226<br>1654472717<br>1648840438<br>1608699595<br>1660358347<br>1656935446<br>1638015360<br>1612305650<br>808334221<br>810654609<br>763380574<br>763917813<br>1638586485	

Ceratotherium simum simum	Southern white rhinoceros	XM_004444041.1	XM_004444040.2	
Cercocebus atys	Sooty mangabey	XM_012071277.1	XM_012071275.1	
Chinchilla lanigera	Long-tailed chinchilla	XM_005403075.1	XM_005415327.1	
Chlorocebus sabaeus	Green monkey	XM_008005062.1	XM_008005060.1	
Chrysochloris asiatica	Cape golden mole	XM_006865484.1	XM_006878296.1	
Colobus angolensis palliatus	Angolan colobus	XM_011948481.1	XM_011948480.1	
Condylura cristata	Star-nosed mole	XM_004695933.2	XM_004690624.1	
Cricetulus griseus	Chinese hamster	XM_007652908.1	XM_003512415.1	
Dasypus novemcinctus	Nine-banded armadillo	XM_004484978.2	XM_004484979.2	
Dipodomys ordii	Ord's kangaroo rat	XM_013023063.1	XM_013023107.1	
Echinops telfairii	Lesser hedgehog tenrec	XM_004709724.1	XM_004709722.1<br>XM_004709723.1	
Eptesicus fuscus	Big brown bat	XM_008142901.1	XM_008142902.1	
Equus caballus	Horse	XM_005614958.1	XM_005614972.2	
Equus przewalskii	Przewalski's horse	XM_008518469.1	XM_008518468.1	
Erinaceus europaeus	European hedgehog	XM_007539798.1	trace archives gi:<br>909569621<br>916868228<br>9197477<br>956896226<br>921926170<br>931620789<br>937804449<br>955617607<br>956096237<br>921892597<br>937763996<br>955550463<br>919608302<br>914608357<br>929672032	
Felis catus	Cat	XM_006938419.2	NM_001309044.1	
Galeopterus variegatus	Sunda flying lemur	XM_008577706.1	XM_008577707.1	
Gorilla gorilla	Western gorilla	KU285431	KU285439	KU285447
Heterocephalus glaber	Naked mole rat	XM_004844096.1	XM_004843902.1	

Homo sapiens	Human	KU285432	KU285440	KU285448
Hoolock leuconedys	eastern hoolock gibbon	SRX590196, SRX590198	SRX590196, SRX590198	
Hylobates moloch	silvery gibbon	SRX590190	SRX590190	
Hylobates pileatus	pileated gibbon	SRX590199	SRX590199	
Jaculus jaculus	Lesser Egyptian jerboa	XM_004673237.2	XM_004673240.2	
Leptonychotes weddellii	Weddell seal	XM_006752446.1	XM_006752452.1	
Lipotes vexillifer	Yangtze River dolphin	XM_007472205.1	XM_007472206.1	
Loxodonta africana	African bush elephant	XM_010599841.1	XM_003420333.1	
Macaca fascicularis	Crab-eating macaque	XM_005572512.1	XM_005596020.1	
Macaca mulatta	Rhesus macaque	NM_001145254.1	XR_012318.2	
Macaca nemestrina	Pig-tailed macaque	XM_011762566.1	XM_011762579.1	
Mandrillus leucophaeus	Drill	XM_011964508.1	XM_011964507.1	
Mesocricetus auratus	Golden hamster	KP639582.1	KP639583.1	
Microcebus murinus	Gray mouse lemur	XM_012782236.1	XM_012782142.1	
Microtus ochrogaster	Prairie vole	XM_005344400.1	XM_005344399.1	
Monodelphis domestica	Gray short-tailed opossum	XM_001374185.2	XM_001374136.2	
Mus musculus	House mouse	KU285433	KU285441	
Mustela putorius furo	Ferret	XM_004828342.2	XM_004828343.2	
Myotis lucifugus	Little brown bat	XM_006103820.1	XM_006109863.2	
Nannospalax galili	Upper Galilee mountains blind mole rat	XM_008839408.1	XM_008839315.1	
Nomascus leucogenys	White-cheeked gibbon	KU285434 SRX590181, SRX590192	KU285442 SRX590181, SRX590192	
Ochotona princeps	American pika	XM_004595402.1	XM_004595401.1	
Octodon degus	Degu	XM_012515727.1	XM_004649001.2	
Odobenus rosmarus	Pacific walrus	XM_004395897.2	XM_004395896.2	
Orcinus orca	killer whale	XM_004286868.2	XM_004286872.2	
Ornithorhynchus anatinus	Platypus	XM_007666896.1	XM_007666895.1	
Orycteropus afer afer	Aardvark	XM_007938249.1	XM_007938250.1	
Oryctolagus cuniculus	Rabbit	XM_008250523.1	XM_002722739.2	
Otolemur garnettii	Garnett's greater galago	XM_003796014.2	XM_003804027.2	
Ovis aries	Sheep	XM_012097734.1	XM_004023416.2	
Pan paniscus	Bonobo	KU285435	KU285443	KU285449
Pan troglodytes	Chimpanzee	KU285436	KU285444	KU285450
Panthera tigris altaica	Amur tiger	XM_007079644.1	XM_007099226.1	

Pantholops hodgsonii	Chiru	XM_005985762.1	XM_005955269.1	
Papio anubis	Olive baboon	XM_003907319.2	XM_003907318.2	
Peromyscus maniculatus bairdii	Prairie deer mouse	XM_006999075.1	XM_006970707.1	
Physeter catodon	Sperm whale	XM_007131038.1	XM_007117167.1	
Pongo pygmaeus abelii	Sumatran orangutan	KU285437	KU285445	KU285451
Propithecus coquereli	Coquerel's sifaka	XM_012663442.1	XM_012663575.1	
Pteropus alecto	Black flying fox	XM_006927015.1	XM_006927014.1	
Pteropus vampyrus	Large flying fox	XM_011355737.1	XM_011355738.1	
Rattus norvegicus	Norway rat	NM_001145334.1	NM_181476.1	
Rhinopithecus roxellana	Golden snub-nosed monkey	XM_010368366.1	XM_010368365.1	
Saimiri boliviensis	Black-capped squirrel monkey	XM_003932150.2	XM_003932151.2	
Sarcophilus harrisii	Tasmanian devil	XM_003761068.1	XM_003761067.1	
Sorex araneus	Common shrew	XM_012933294.1	XM_004611156.1	
Spermophilus tridecemlineatus	thirteen-lined ground squirrel	XM_005343026.1	XM_005343027.1	
Sus scrofa	Wild boar	NM_001145381.1	AK397813.1	
Symphalangus syndactylus	Siamang	KU285438<br>SRX590195, SRX590189	KU285446<br>SRX590195, SRX590189	KU285452<br>SRX590195, SRX590189
Tarsius syrichta	Philippine tarsier	XM_008050396.1	XM_008050397.1	
Trichechus manatus latirostris	Florida manatee	XM_004391326.2	XM_012558036.1	
Tupaia chinensis	Chinese treeshrew	XM_006149277.1	XM_006149278.1	
Tursiops truncatus	Common bottlenose dolphin	XM_004332398.1	XM_004332393.1	
Ursus maritimus	polar bear	XM_008688842.1	XM_008688841.1	
Vicugna pacos	Alpaca	XM_006220341.1	XM_006220342.1	

Zebra fish HCA_1 sequence (NM_001163292.1) was used as query sequence in discontinuous megablast in a standard nucleotide BLAST for mining orthologous sequences. HCAR sequences of various mammalian species were obtained using the respective mouse ortholog nucleotide sequences (NM_175520.4, NM_030701.3) or human HCAR3 (NM_006018.2) as query sequence in discontinuous megablast in a standard nucleotide BLAST, a Trace Archive Nucleotide BLAST of mammalian trace archives or a Sequence Read Archive Nucleotide BLAST. Trace files of sequences producing significant alignments were downloaded followed by assembly, analysis (using SeqManPro of the DNAStar Lasergene Software Suite for Sequence Analysis 7.1.) and manual proof-reading. Trace identifier, NCBI accession or SRA accession numbers are listed
