# Supplementary material for: Metabolites of lactic acid bacteria present in fermented foods are highly potent agonists of human hydroxycarboxylic acid receptor 3
Source: PLoS Genet. 2019 May 23;15(5):e1008145. doi: 10.1371/journal.pgen.1008145 (PMC6532841; doi:10.1371/journal.pgen.1008145)
Supplement: S3 Table — (PDF) [file pgen.1008145.s010.pdf]

Supplementary Table S3

Maximum likelihood estimates of d_N/ds ratios (ω) for 88 mammalian HCA_1 and HCA_2 orthologs, for 5 ape HCA_1, HCA_2 and HCA_3 orthologs and for 5 Hylobatidae HCA_1 and HCA_2 orthologs under different branch-specific models using PAML.

**branch models**	*p*	l	κ	Estimates of Parameters	hypothesis	χ^2	*P*-Value
**HCA_1**							
88 mammalian species							
One ratio: ω_0	142	-18285.29	4.08	$\omega_0 = 0.106$			
Two ratios: ω_0, $\omega_{Hominidae}$	143	-18284.32	4.08	$\omega_0 = 0.105$, $\omega_{Hominidae} = 0.169$	$\omega_0 \neq \omega_{Hominidae}$	1.94	0.1637
Two ratios: ω_0, $\omega_{Hylobatidae}$	143	-18284.04	4.08	$\omega_0 = 0.105$, $\omega_{Hylobatidae} = 0.233$	$\omega_0 \neq \omega_{Hylobatidae}$	2.51	0.1131
Two ratios: ω_0, $\omega_{Hominoidea}$	143	-18282.81	4.09	$\omega_0 = 0.105$, $\omega_{Hominoidea} = 0.189$	$\omega_0 \neq \omega_{Hominoidea}$	4.97	0.0258
**HCA_2**							
88 mammalian species							
One ratio: ω_0	142	-18378.75	3.43	$\omega_0 = 0.091$			
Two ratios: ω_0, $\omega_{Hominidae}$	143	-18378.74	3.43	$\omega_0 = 0.091$, $\omega_{Hominidae} = 0.087$	$\omega_0 \neq \omega_{Hominidae}$	0.02	0.8875
Two ratios: ω_0, $\omega_{Hylobatidae}$	143	-18378.73	3.43	$\omega_0 = 0.091$, $\omega_{Hylobatidae} = 0.099$	$\omega_0 \neq \omega_{Hylobatidae}$	0.05	0.8231
Two ratios: ω_0, $\omega_{Hominoidea}$	143	-18378.69	3.43	$\omega_0 = 0.091$, $\omega_{Hominoidea} = 0.099$	$\omega_0 \neq \omega_{Hominoidea}$	0.13	0.7184
**HCA_1-HCA_2-HCA_3**							
**5 apes**							
**branch models**							
One ratio: ω_0	29	-3047.70	2.32	$\omega_0 = 0.135$			
Two ratios: ω_{HCA1}, $\omega_{HCA2/HCA3}$	30	-3045.26	2.36	$\omega_{HCA1} = 0.095$, $\omega_{HCA2/HCA3} = 0.184$	$\omega_{HCA1} \neq \omega_{HCA2/HCA3}$	4.88	0.0272
Two ratios: ω_{HCA2}, $\omega_{HCA1/HCA3}$	30	-3047.70	2.32	$\omega_{HCA2} = 0.131$, $\omega_{HCA1/HCA3} = 0.137$	$\omega_{HCA2} \neq \omega_{HCA1/HCA3}$	0.01	0.9203
Two ratios: ω_{HCA3}, $\omega_{HCA1/HCA2}$	30	-3043.99	2.36	$\omega_{HCA3} = 0.257$, $\omega_{HCA1/HCA2} = 0.103$	$\omega_{HCA3} \neq \omega_{HCA1/HCA2}$	7.43	0.0064
Two ratios: $\omega_{HCA3} = 1$, $\omega_{HCA1/HCA2}$	29	-3056.45	2.49	$\omega_{HCA3} = 1$, $\omega_{HCA1/HCA2} = 0.104$	$\omega_{HCA3} \neq 1$	24.92	0.0001
Two ratios:	30	-3045.02	2.33	$\omega_0 = 0.124$, $\omega_{siamangHCA2/HCA3} = 0.775$	$\omega_0 \neq \omega_{siamangHCA2/HCA3}$	5.36	0.0206

ω_0, $\omega_{siamangHCA2/HCA3}$ Two ratios: ω_0, $\omega_{siamangHCA2/HCA3} = 1$	29	-3045.07	2.33	$\omega_0 = 0.124$, $\omega_{siamangHCA2/HCA3} = 1$	$\omega_{siamangHCA2/HCA3} \neq 1$	0.09	0.7642
**HCA$_1$**							
5 gibbons							
One ratio: ω_0	10	-1467.86	3.03	$\omega_0 = 0.207$			
Two ratios: ω_0, $\omega_{siamang}$	11	-1467.40	3.03	$\omega_0 = 0.138$, $\omega_{siamang} = 0.415$	$\omega_0 \neq \omega_{siamang}$	0.93	0.3349
Two ratios: ω_0, $\omega_{siamang} = 1$	10	-1467.82	3.17	$\omega_0 = 0.105$, $\omega_{siamang} = 1$	$\omega_{siamang} \neq 1$	0.84	0.3594
**HCA$_2$**							
5 gibbons							
One ratio: ω_0	8	-1597.26	2.42	$\omega_0 = 0.114$			
Two ratios: ω_0, $\omega_{siamang}$	9	-1593.68	2.42	$\omega_0 = 0.048$, $\omega_{siamang} = 0.643$	$\omega_0 \neq \omega_{siamang}$	7.16	0.0075
Two ratios: ω_0, $\omega_{siamang} = 1$	8	-1593.81	2.46	$\omega_0 = 0.048$, $\omega_{siamang} = 1$	$\omega_{siamang} \neq 1$	0.26	0.6101

[a] LRT tests were performed between nested models. ω, d_N/d_S ratio; ω_0, indicates ω of all other branches (the ones that are not specifically labeled in a model). *p*, number of free parameters; ℓ, Log likelihood; κ, transition to transversion rate ratio; * indicates *P*>0.95; ** indicates *P*>0.99); corresponding tree in NEWICK notation: ((((((Hsapi3,Ptrog3),Ggori3),Ppygm3),Ssynd3),((((Hsapi2,Ptrog2),Ggori2),Ppygm2),Ssynd2)),((((Hsapi1,Ptrog1),Ggori1),Ppygm1),Ssynd1)); Hsapi: Homo sapiens, Ptrog: Pan troglodytes, Ggori: Gorilla gorilla, Ppygm: Pongo pygmaeus, Ssynd: Symphalangus syndactylus; corresponding tree in NEWICK notation: ((hoolock-gibbon1,silvery-gibbon1,(pileated-gibbon1)),siamang1,white-cheeked-gibbon1);
((hoolock-gibbon2,silvery-gibbon2,(pileated-gibbon2)),siamang2,white-cheeked-gibbon2);
