# Supplementary material for: Metabolites of lactic acid bacteria present in fermented foods are highly potent agonists of human hydroxycarboxylic acid receptor 3
Source: PLoS Genet. 2019 May 23;15(5):e1008145. doi: 10.1371/journal.pgen.1008145 (PMC6532841; doi:10.1371/journal.pgen.1008145)
Supplement: S4 Table — (PDF) [file pgen.1008145.s011.pdf]

Supplementary Table S4

Bacterial species carrying genes for EC 1.1.1.28 that were detected in human gut microbiota of the curated Metagenomic Dataset

**UniRefID**	**taxonomy**		**taxID**	**RepID**	**annotated Function**	**members**
A4SHJ2	*Aeromonas salmonicida (strain A449)*	ldhA	645	A4SHJ2_AERS4	D-lactate dehydrogenase	4
D4IK03	*Alistipes shahii WAL 8301*	AL1_06570	717959	D4IK03_9BACT	Lactate dehydrogenase and related dehydrogenases	1
Q5HLA0	*Staphylococcus epidermidis (strain ATCC 35984 / RP62A)*	ldhD	1385	LDHD_STAEQ	D-lactate dehydrogenase	8
A0A0U0PXB9	*Streptococcus pneumoniae*	ldhA_4	91061	A0A0U0PXB9_STREE	D-lactate dehydrogenase	3
R7NW57	*Bacteroides vulgatus CAG:6*	BN728_00129	816	R7NW57_9BACE	Dehydrogenase	3
A0A173R861	*Blautia hydrogenotrophica*	ldhA	572511	A0A173R861_9FIRM	D-lactate dehydrogenase	4
D4LSS4	*Blautia obeum A2-162*	CK5_25280	657314	D4LSS4_9FIRM	Lactate dehydrogenase and related dehydrogenases	1
A0A0R2J7L9	*Carnobacterium maltaromaticum*	IV76_GL002156	2751	A0A0R2J7L9_CARML	D-isomer specific 2-hydroxyacid dehydrogenase, catalytic domain protein	3
D4LHM9	*Ruminococcus sp. SR1/5*	CK1_11640	186802	D4LHM9_9FIRM	Lactate dehydrogenase and related dehydrogenases	2
A0A1E7S4G9	*Butyribacterium methylotrophicum*	ldhA	186802	A0A1E7S4G9_BUTME	D-lactate dehydrogenase	3
A0A174AVH0	*Blautia obeum*	ldhA	186802	A0A174AVH0_9FIRM	D-lactate dehydrogenase	4
A5ZQ76	*Blautia obeum ATCC 29174*	pdxB	186802	A5ZQ76_9FIRM	4-phosphoerythronate dehydrogenase	4
D5HDL3	*Coprococcus sp. ART55/1*	CCU_13850	33042	D5HDL3_9FIRM	Lactate dehydrogenase and related dehydrogenases	3
C9Y542	*Cronobacter turicensis (strain DSM 18703 / LMG 23827 / z3032)*	ldhA	413502	C9Y542_CROTZ	D-lactate dehydrogenase	2
G9Y1R0	*Hafnia alvei ATCC 51873*	HMPREF0454_00477	91347	G9Y1R0_HAFAL	D-lactate dehydrogenase	4
R6H4R1	*Blautia sp. CAG:52*	BN690_00348	765820	R6H4R1_9FIRM	Uncharacterized protein	2

R6TCX2	*Ruminococcus sp. CAG:57*	BN714_00041	129701	R6TCX2_9FIRM	D-lactate dehydrogenase	2
A0A175AAG2	*Clostridium innocuum*	ldhA	128827	A0A175AAG2_CLOIN	D-isomer specific 2-hydroxyacid dehydrogenase NAD-binding subunit	3
A0A174Z3P7	*[Eubacterium] eligens*	ldhA	1730	A0A174Z3P7_9FIRM	D-lactate dehydrogenase	3
A0A1S8HW56	*Enterococcus faecium (Streptococcus faecium)*	B1P95_07035	1239	A0A1S8HW56_ENTFC	Lactate dehydrogenase	10
R6BRE7	*Firmicutes bacterium CAG:56*	BN708_00872	1239	R6BRE7_9FIRM	D-lactate dehydrogenase	2
Q7P6Z0	*Fusobacterium nucleatum subsp. vincentii ATCC 49256*	FNV1748	848	Q7P6Z0_FUSNV	D-lactate dehydrogenase	7
E5BHL4	*Fusobacterium gonidiaformans 3-1-5R*	ldhA	848	E5BHL4_9FUSO	D-lactate dehydrogenase	2
A5TXC5	*Fusobacterium nucleatum subsp. polymorphum ATCC 10953*	FNP_1778	851	A5TXC5_FUSNP	D-lactate dehydrogenase	2
A0A0A3B643	*Gallibacterium anatis*	CF557_11150	155493	A0A0A3B643_9PAST	2-hydroxyacid dehydrogenase	7
P52643	*Escherichia coli (strain K12)*	ldhA	1236	LDHD_ECOLI	D-lactate dehydrogenase	28
C5NVX3	*Gemella haemolysans ATCC 10379*	ldhA	546270	C5NVX3_9BACL	D-lactate dehydrogenase	1
I2NIP6	*Haemophilus paraphrohaemolyticus HK411*	ldhA	1095743	I2NIP6_9PAST	D-lactate dehydrogenase, fermentative	1
U4RME6	*Haemophilus parasuis MN-H*	ldhA	1275967	U4RME6_HAEPR	D-lactate dehydrogenase	1
D0Z839	*Edwardsiella tarda (strain EIB202)*	ldhA	1903412	D0Z839_EDWTE	D-lactate dehydrogenase	4
A0A087FL13	*Klebsiella variicola*	ldhA	570	A0A087FL13_KLEVA	2-hydroxyacid dehydrogenase	4
A0A2D2GQF3	*Klebsiella pneumoniae*	CTI55_14035	573	A0A2D2GQF3_KLEPN	2-hydroxyacid dehydrogenase	3
A0A0R2FDE7	*Weissella confusa (Lactobacillus confusus)*	BKY29_07830	186826	A0A0R2FDE7_WEICO	D-lactate dehydrogenase	3
C2EUK0	*Lactobacillus vaginalis DSM 5837 = ATCC 49540*	ldhA	1578	C2EUK0_9LACO	D-lactate dehydrogenase	2

A0A0R2E8D7	*Lactobacillus fermentum*	ldhA	1578	A0A0R2E8D7_LACFE	D-lactate dehydrogenase	3
A4GXJ0	*Lactobacillus reuteri*	ldhD3	1578	A4GXJ0_LACRE	D-lactate dehydrogenase	4
P17584	*Lactobacillus paracasei*		1578	DHD2_LACPA	D-2-hydroxyisocaproate dehydrogenase	8
Q88VJ2	*Lactobacillus plantarum (strain ATCC BAA-793 / NCIMB 8826 / WCFS1)*	ldhD	1578	LDHD_LACPL	D-lactate dehydrogenase	9
M5AXQ3	*Lactobacillus brevis KB290*	LVISKB_0201	1580	M5AXQ3_LACBR	D-lactate dehydrogenase	5
J9W550	*Lactobacillus buchneri CD034*	ldhD2	1581	J9W550_LACBU	D-lactate dehydrogenase	2
P26297	*Lactobacillus delbrueckii subsp. bulgaricus*	ldhA	1585	LDHD_LACDA	D-lactate dehydrogenase	2
A0A1D7ZUQ4	*Lactobacillus fermentum*	LACFE_CDS0107	1613	A0A1D7ZUQ4_LACFE	D-2-hydroxyisocaproate dehydrogenase	6
P30901	*Lactobacillus helveticus (Lactobacillus suntoryeus)*		1587	LDHD_LACHE	D-lactate dehydrogenase	6
D6S2A4	*Lactobacillus jensenii JV-V16*	ldhA	525329	D6S2A4_9LACO	D-lactate dehydrogenase	1
E3C8P4	*Lactobacillus oris PB013-T2-3*	ldhA	908339	E3C8P4_9LACO	D-lactate dehydrogenase	1
A0A143PXG9	*Lactobacillus reuteri*	ADV92_02125	1598	A0A143PXG9_LACRE	Lactate dehydrogenase	4
F5VBS9	*Lactobacillus salivarius NIAS840*	NIAS840_00208	1624	F5VBS9_9LACO	D-lactate dehydrogenase	2
A0A1V9SKC4	*Lactobacillus salivarius*	B6U37_09610	1624	A0A1V9SKC4_9LACO	Lactate dehydrogenase	2
G2KTX9	*Lactobacillus sanfranciscensis (strain TMW 1.1304)*	ldhD	1625	G2KTX9_LACSM	D-lactate dehydrogenase	2
G2KUM4	*Lactobacillus sanfranciscensis (strain TMW 1.1304)*	LSA_12510	714313	G2KUM4_LACSM	D-lactate dehydrogenase	1
H1WTC8	*Leuconostoc citreum LBAE C10*	ldhA	33964	H1WTC8_LEUCI	D-lactate dehydrogenase	2
B1MW55	*Leuconostoc citreum (strain KM20)*	ddh	349519	B1MW55_LEUCK	Lactate dehydrogenase	1
B1MXH0	*Leuconostoc citreum (strain KM20)*	ldhD	349519	B1MXH0_LEUCK	D-lactate dehydrogenase	1

P51011	*Leuconostoc mesenteroides subsp. cremoris*		33965	LDHD_LEUMC	D-lactate dehydrogenase	1
M9SCZ3	*Methanomethylophilus alvus (strain Mx1201)*	MMALV_05640	1236689	M9SCZ3_METAX	D-lactate dehydrogenase	1
J7SK16	*Morganella morganii subsp. morganii KT*	MU9_2111	582	J7SK16_MORMO	D-lactate dehydrogenase	4
B4RN51	*Neisseria gonorrhoeae (strain NCCP11945)*	NGK_1561	485	B4RN51_NEIG2	D-lactate dehydrogenase	4
F9Z6L8	*Odoribacter splanchnicus (strain ATCC 29572 / DSM 20712 / CIP 104287 / JCM 15291 / NCTC 10825 / 1651/6) (Bacteroides splanchnicus)*	Odosp_0571	283168	F9Z6L8_ODOSD	D-lactate dehydrogenase	2
A0A0A1SAC7	*Paeniclostridium sordellii (Clostridium sordellii)*	ldhA	1505	A0A0A1SAC7_PAESO	D-lactate dehydrogenase	3
T4VW16	*Paraclostridium bifermentans ATCC 19299*	ldhD	1233170	T4VW16_PARBF	D-lactate dehydrogenase	1
Q59642	*Pediococcus acidilactici*	ldhD	1254	LDHD_PEDAC	D-lactate/D-glycerate dehydrogenase	1
A0A1A9AXK2	*Plesiomonas shigelloides (Aeromonas shigelloides)*	ldhA	703	A0A1A9AXK2_PLESH	D-lactate dehydrogenase	2
Z4WUP3	*Porphyromonas catoniae ATCC 51270*	ldhA	887901	Z4WUP3_9PORP	D-lactate dehydrogenase LdhA	1
A0A1H9E2A1	*Prevotella bryantii B14*	SAMN05444375_10698	752555	A0A1H9E2A1_PREBR	D-lactate dehydrogenase	2
F0H7X4	*Prevotella denticola CRIS 18C-A*	ldhA	28129	F0H7X4_9BACT	D-lactate dehydrogenase	2
F0H9I0	*Prevotella denticola CRIS 18C-A*	ldhA	28129	F0H9I0_9BACT	D-lactate dehydrogenase	3
F8N944	*Prevotella multisaccharivorax DSM 17128*	Premu_1191	688246	F8N944_9BACT	D-lactate dehydrogenase	1
D3IMR2	*Prevotella sp. oral taxon 317 str. F0108*	ldhA	575615	D3IMR2_9BACT	D-lactate dehydrogenase	1
A0A1Z1SX41	*Proteus mirabilis*	AM402_12865	583	A0A1Z1SX41_PROMI	2-hydroxyacid dehydrogenase	3

K8WA49	*Providencia sneebia DSM 19967*	OO7_09120	1141660	K8WA49_9GAMM	D-lactate dehydrogenase	1
V7D6I6	*Pseudomonas taiwanensis SJ9*	O164_20940	286	V7D6I6_9PSED	D-lactate dehydrogenase	3
A0A0S4V152	*Ralstonia solanacearum (Pseudomonas solanacearum)*	ldhA	48736	A0A0S4V152_RALSL	Fermentative D-lactate dehydrogenase, NAD-dependent	4
I6H5A7	*Shigella flexneri 1235-66*	SF123566_2205	766154	I6H5A7_SHIFL	D-lactate dehydrogenase (combined with I6H5A8)	1
Q6G6F1	*Staphylococcus aureus (strain MSSA476)*	ldhD	1280	LDHD_STAAS	D-lactate dehydrogenase	4
Q8E6A9	*Streptococcus agalactiae serotype III (strain NEM316)*	ldhD	1311	LDHD_STRA3	D-lactate dehydrogenase	7
A0A1U0IBM9	*Mycobacterium abscessus subsp. abscessus*	ldhD	1783272	A0A1U0IBM9_9MYCO	D-3-phosphoglycerate dehydrogenase (SerA)	5
F2NY74	*Treponema succinifaciens (strain ATCC 33096 / DSM 2489 / 6091)*	Tresu_1183	869209	F2NY74_TRES6	D-lactate dehydrogenase	1
A0A173TYG0	*Turicibacter sanguinis*	ldhA	154288	A0A173TYG0_9FIRM	D-lactate dehydrogenase	2
A0A1C5X6H7	*uncultured Clostridium sp.*	ldhA_2		A0A1C5X6H7_9CLOT	D-lactate dehydrogenase	2
F4N5A3	*Yersinia enterocolitica W22703*	ldhA	913028	F4N5A3_YEREN	D-lactate dehydrogenase	1
sequences which were not included in tree but nevertheless detected in human microbiome data set						
A0A015PCZ5	*Bacteroides*	M080_5071	816	A0A015PCZ5_BACFG	D-isomer specific 2-hydroxyacid dehydrogenase, catalytic domain protein	3
D1ND28	*Haemophilus influenzae HK1212*	dld	456482	D1ND28_HAEIF	D-lactate dehydrogenase	1
W1H0L2	*Klebsiella pneumoniae*		573	W1H0L2_KLEPN	D-lactate dehydrogenase	3
W1DSP9	*Klebsiella pneumoniae IS43*		1432552	W1DSP9_KLEPN	D-lactate dehydrogenase	1
W1EEI5	*Klebsiella pneumoniae IS53*		1432554	W1EEI5_KLEPN	D-lactate dehydrogenase	1
W1H2U2	*Klebsiella pneumoniae ISC21*		1432558	W1H2U2_KLEPN	D-lactate dehydrogenase	1

A0A0U3DDR3	*Lactobacillus delbrueckii subsp. bulgaricus*	AT236_00768	1585	A0A0U3DDR3_LACDE	D-hydroxyisocaproate dehydrogenase	3
T0D2K8	*Paeniclostridium sordellii ATCC 9714*	ldhD	1292036	T0D2K8_PAESO	D-lactate dehydrogenase	1
P06149	*Proteobacteria*	dld	1224	DLD_ECOLI	Quinone-dependent D-lactate dehydrogenase	21
C0B123	*Proteus penneri ATCC 35198*	dld	471881	C0B123_9GAMM	D-lactate dehydrogenase	1
I6H5A8	*Shigella flexneri 1235-66*	SF123566_2206	766154	I6H5A8_SHIFL	D-lactate dehydrogenase domain protein	1
K4Q9L2	*Streptococcus dysgalactiae subsp. equisimilis AC-2713*	ddh	759913	K4Q9L2_STREQ	D-lactate dehydrogenase	1

EC 1.1.1.28 is D-lactate dehydrogenase, the enzyme catalyzing the conversion between D-lactate and pyruvate. For *Lactobacillus plantarum* SK002, it has been shown that this enzyme also can also convert phenylpyruvate to D-phenyllactate. The BRENDA database [46] also lists this activity for *L. pentosus* and *L. jensenii*, in addition to *Pediococcus acidilactici* and *P. pentosaceus*. From the current literature it is uncertain whether all D-lactate dehydrogenases have a similar potential to also produce phenyllactate or whether this is a specific trait of certain Lactobacillaceae enzymes. UniProtKB, on the other hand, lists 1,809 entries with the EC 1.1.1.28 annotation. These comprise 1,443 unique sequences (UniRef100). In addition to the annotated genes, UniProtKB lists 481 proteins with identical sequences, which are not annotated as such. These total of 2,290 proteins are found in 1,643 different organisms, including mostly bacteria, 13 archaea and 15 eukaryotic organisms, such as fungi, amoebozoa, and diatoms. Metagenomic data from 1,885 human stool samples from the curated Metagenomic Dataset (https://github.com/waldronlab/curatedMetagenomicData [47] were searched for the 1,443 (EC 1.1.1.28)-related UniRef100 entries. 92 of the UniProt entries for EC 1.1.1.28 were found in at least one human microbiome data set. These 92 unique sequences represent 271 different UniProtKB proteins that are found in different strains of 107 bacterial species and insufficiently described bacteria.
