# Supplementary material for: Metabolites of lactic acid bacteria present in fermented foods are highly potent agonists of human hydroxycarboxylic acid receptor 3
Source: PLoS Genet. 2019 May 23;15(5):e1008145. doi: 10.1371/journal.pgen.1008145 (PMC6532841; doi:10.1371/journal.pgen.1008145)
Supplement: S5 Table — (PDF) [file pgen.1008145.s012.pdf]

Supplementary Table S5

Testing of PLA-containing human urine and plasma samples on transiently with human HCA_2 or HCA_3 or empty vector transfected CHO-K1 cells

		**HCA_2**	**HCA_3**	**empty vector**	**HCA_2 (PTX)**	**HCA_3 (PTX)**	**empty vector (PTX)**
dilution	PLA	% of forskolin stimulated w/o	% of forskolin stimulated w/o	% of forskolin stimulated w/o	% of forskolin stimulated w/o	% of forskolin stimulated w/o	% of forskolin stimulated w/o
	[nM]						
**urine (80 min containing 285 µM PLA)**							
1:20,480	13.9	82.3 ± 3.3	91.8 ± 3.6	96.4 ± 5.3	92.1 ± 6.3	97.7 ± 4.2	100.6 ± 2.7
1:10,240	27.8	99.3 ± 5.5	84.8 ± 4.1	96.9 ± 5.0	92.4 ± 8.9	95.3 ± 2.4	95.4 ± 0.5
1:5,120	55.7	95.6 ± 8.0	81.1 ± 3.5	95.1 ± 4.4	93.5 ± 4.6	92.3 ± 2.8	97.9 ± 3.6
1:2,560	111.3	104.9 ± 1.7	68.8 ± 1.8	98.4 ± 4.0	91.1 ± 8.6	87.9 ± 0.7	96.7 ± 3.5
1:1,280	222.7	93.3 ± 1.4	65.2 ± 0.6	91.7 ± 1.5	88.7 ± 4.8	86.4 ± 0.5	96.8 ± 6.5
1:640	445.3	92.2 ± 5.9	56.0 ± 2.4	86.8 ± 3.3	82.1 ± 2.0	85.4 ± 3.8	99.7 ± 6.3
1:320	890.6	85.5 ± 2.7	48.4 ± 3.6	80.5 ± 2.3	87.7 ± 5.2	86.4 ± 2.9	93.4 ± 5.4
1:160	1781.3	72.1 ± 4.5	43.3 ± 4.0	74.1 ± 2.9	82.3 ± 5.9	76.5 ± 5.9	89.0 ± 9.6
1:80	3562.5	54.8 ± 2.3	37.4 ± 2.8	66.6 ± 2.7	83.3 ± 9.53	75.7 ± 7.0	84.3 ± 9.5
**urine (340 min containing 33 µM PLA)**							
1:1,280	25.8	91.2 ± 6.8	87.4 ± 8.8	81.7 ± 2.5	n.d.	n.d.	n.d.
1:640	51.6	87.1 ± 0.8	74.5 ± 2.0	80.1 ± 4.7	n.d.	n.d.	n.d.
1:320	103.1	75.5 ± 2.4	58.7 ± 1.9	71.2 ± 2.0	n.d.	n.d.	n.d.
1:160	206.3	62.2 ± 4.0	52.8 ± 0.1	60.5 ± 4.5	n.d.	n.d.	n.d.
**urine (510 min containing 3.4 µM PLA)**							
1:1,280	2.7	94.6 ± 5.0	85.9 ± 4.2	82.4 ± 9.3	n.d.	n.d.	n.d.
1:640	5.4	87.4 ± 0.5	77.3 ± 1.1	70.6 ± 2.1	n.d.	n.d.	n.d.
1:320	10.8	85.0 ± 5.70	75.9 ± 7.0	76.1 ± 5.2	n.d.	n.d.	n.d.
1:160	21.5	76.9 ± 3.0	67.5 ± 7.5	72.0 ± 0.7	n.d.	n.d.	n.d.
**plasma (30 min containing 23 µM PLA)**							
1:20,480	1.1	96.2 ± 5.9	96.0 ± 0.3	99.3 ± 0.9	106.3 ± 3.0	102.5 ± 2.4	101.4 ± 0.1
1:10,240	2.2	102.9 ± 2.8	90.2 ± 2.5	101.1 ± 2.7	101.5 ± 2.5	99.5 ± 4.1	95.7 ± 0.5
1:5,120	4.5	97.8 ± 0.1	84.4 ± 3.9	101.0 ± 0.5	97.6 ± 3.1	94.9 ± 0.1	91.0 ± 4.8
1:2,560	9.0	104.4 ± 5.1	88.0 ± 1.9	109.1 ± 5.6	102.0 ± 3.2	96.5 ± 4.0	98.6± 4.5
1:1,280	18.0	102.0 ± 9.3	72.8 ± 2.5	102.5 ± 1.3	99.4 ± 4.5	94.5 ± 2.3	95.7 ± 0.2
1:640	35.9	92.0 ± 3.1	67.1 ± 1.8	93.2 ± 1.6	100.2 ± 1.7	90.9 ± 0.5	97.5 ± 2.3
1:320	71.9	79.5 ± 1.2	57.6 ± 6.8	85.7 ± 3.5	98.9 ± 3.1	95.7 ± 1.3	101.2 ± 2.8
1:160	143.8	67.4 ± 4.7	47.1 ± 3.5	73.0 ± 0.6	95.5 ± 4.4	91.7 ± 6.0	98.0 ± 4.3
1:80	287.5	47.8 ± 4.1	33.3 ± 0.9	58.9 ± 3.6	88.0 ± 5.8	87.1 ± 4.3	95.8 ± 0.3

**plasma (0 min containing 0.42 µM PLA)**							
1:320	1.3	75.1 ± 4.7	90.4 ± 1.0	91.0 ± 0.1	n.d.	n.d.	n.d.
1:160	2.6	60.4 ± 6.8	70.1 ± 0.6	69.8 ± 1.8	n.d.	n.d.	n.d.
**plasma (60 min containing 8.9 µM PLA)**							
1:320	27.8	80.1 ± 1.2	73.5 ± 5.4	83.9 ± 6.2	n.d.	n.d.	n.d.
1:160	55.6	75.4 ± 0.7	59.3 ± 3.2	67.4 ± 3.7	n.d.	n.d.	n.d.
**plasma (120 min containing 2.9 µM PLA)**							
1:320	9.1	76.8 ± 0.8	77.6 ± 6.3	82.4 ± 4.7	n.d.	n.d.	n.d.
1:160	18.1	67.7 ± 4.2	61.5 ± 5.9	66.4 ± 4.7	n.d.	n.d.	n.d.
**plasma (180 min containing 0.8 µM PLA)**							
1:320	2.5	78.1 ± 5.1	80.4 ± 2.3	88.6 ± 7.9	n.d.	n.d.	n.d.
1:160	5.0	57.0 ± 2.7	70.6 ± 5.5	70.6 ± 2.5	n.d.	n.d.	n.d.
**plasma (240 min containing 0.6 µM PLA)**							
1:320	1.9	66.2 ± 5.9	75.4 ± 3.5	77.7 ± 4.4	n.d.	n.d.	n.d.
1:160	3.8	48.7 ± 1.3	57.1± 2.1	51.6 ± 1.1	n.d.	n.d.	n.d.
**plasma (300 min containing 0.35 µM PLA)**							
1:320	1.1	70.8 ± 8.6	79.8 ± 5.5	85.7 ± 1.3	n.d.	n.d.	n.d.
1:160	2.2	55.7 ± 0.8	63.8 ± 2.7	67.1 ± 4.5	n.d.	n.d.	n.d.
**plasma (360 min containing 0.29 µM PLA)**							
1:320	0.9	77.8 ± 4.7	79.5 ± 6.7	85.7 ± 6.7	n.d.	n.d.	n.d.
1:160	1.8	62.9 ± 3.9	65.2 ± 3.3	67.4 ± 1.1	n.d.	n.d.	n.d.
**plasma (420 min containing 0.31 µM PLA)**							
1:320	1.0	83.8 ± 3.2	89.2 ± 7.2	95.1 ± 3.7	n.d.	n.d.	n.d.
1:160	1.9	60.1 ± 0.8	72.4 ± 3.0	69.6 ± 1.3	n.d.	n.d.	n.d.
**Sauerkraut experiment**							
**plasma (before Sauerkraut containing 0.3 µM PLA)**							
1:160	2.1	64.7 ± 6.5	58.9 ± 2.5	64.1 ± 5.9	110.2 ± 0.7	111.3 ± 4.3	104.4 ± 4.1
1:320	1.1	86.2 ± 2.6	73.5 ± 1.1	87.7 ± 4.2	113.0 ± 3.7	104.7 ± 3.2	103.0 ± 3.6
**plasma (2h postprandial containing 1.4 µM PLA)**							
1:160	8.8	67.8 ± 4.4	59.2 ± 3.6	86.6 ± 3.2	100.2 ± 2.8	107.1 ± 0.4	103.7 ± 4.9
1:320	4.4	90.8 ± 6.1	74.7 ± 3.8	98.5 ± 3.7	103.1 ± 5.9	103.5 ± 2.2	105.1 ± 5.2
**urine (before Sauerkraut containing 1.8 µM PLA)**							
1:160	11.5	56.4 ± 3.3	61.4 ± 3.7	79.0 ± 5.5	91.7 ± 3.5	91.9± 2.9	98.0 ± 2.8
1:320	5.8	78.3 ± 1.7	69.3 ± 4.6	88.1 ± 5.0	95.4 ± 2.0	93.3 ± 1.5	93.0± 2.3
**urine (230 min postprandial containing 14.3 µM PLA)**							
1:160	89.2	71.1 ± 5.3	50.5 ± 2.9	74.5 ± 0.7	95.3 ± 4.6	93.4± 4.9	101.8 ± 3.9
1:320	44.6	89.0 ± 4.9	62.2 ± 3.3	83.5 ± 4.2	95.4 ± 0.3	92.0 ± 3.5	94.9 ± 1.2
**urine (540 min postprandial containing 6.3 µM PLA)**							
1:160	39.2	77.8 ± 0.4	53.9 ± 1.7	80.8 ± 4.9	90.8 ± 2.2	95.0 ± 2.8	88.6 ± 2.6
1:320	19.6	89.5 ± 2.1	62.8 ± 5.9	82.1 ± 6.4	98.7 ± 0.7	92.6 ± 3.1	88.5 ± 3.4
**urine (1080 min postprandial containing 3.1 µM PLA)**							
1:160	19.2	73.8 ± 4.6	63.0 ± 3.2	85.0 ± 1.6	101.6 ± 4.7	108.4 ± 2.0	105.1 ± 2.3
1:320	9.6	83.4 ± 2.1	73.8 ± 2.0	88.7 ± 1.6	102.1 ± 1.5	101.6 ± 3.9	100.9 ± 3.7

**urine (1230 min postprandial containing 1.8 µM PLA)**							
1:160	11.6	63.0 ± 4.5	63.6 ± 3.1	84.2 ± 0.6	n.d.	n.d.	n.d.
1:320	5.8	79.4 ± 3.3	73.5 ± 2.3	87.3 ± 0.3	n.d.	n.d.	n.d.
**urine (180 min postprandial containing 5.0 µM PLA)**							
1:160	31.4	81.1 ± 5.6	60.1 ± 2.4	90.4 ± 5.7	n.d.	n.d.	n.d.
1:320	15.7	87.0 ± 4.6	69.4 ± 2.2	92.8 ± 5.3	n.d.	n.d.	n.d.
**urine (390 min postprandial containing 1.9 µM PLA)**							
1:160	11.8	73.1 ± 4.9	59.2 ± 0.6	80.1 ± 6.0	n.d.	n.d.	n.d.
1:320	5.9	78.1 ± 3.4	70.7 ± 1.6	86.3 ± 0.3	n.d.	n.d.	n.d.
**urine (540 min postprandial containing 1.4 µM PLA)**							
1:160	8.6	69.6 ± 3.3	64.2 ± 2.0	79.0± 3.9	n.d.	n.d.	n.d.
1:320	4.3	85.6 ± 1.6	71.1 ± 2.8	84.1 ± 4.4	n.d.	n.d.	n.d.
**urine (1425 min postprandial containing 3.1 µM PLA)**							
1:160	19.6	77.1 ± 5.0	70.6 ± 2.5	87.6 ± 4.1	n.d.	n.d.	n.d.
1:320	9.8	82.5 ± 2.8	75.6 ± 1.9	93.7 ± 0.9	n.d.	n.d.	n.d.
**urine (360 min postprandial containing 10.3 µM PLA)**							
1:160	64.3	76.7 ± 2.0	53.0 ± 1.8	82.6 ± 3.4	88.6 ± 0.6	93.1 ± 2.4	93.7 ± 2.9
1:320	32.2	82.6 ± 5.2	64.1 ± 1.9	91.7 ± 4.0	91.1 ± 2.7	96.4 ± 1.2	98.4 ± 2.5
**urine (after sauerkraut containing 1.5 µM PLA)**							
1:160	9	68.7 ± 2.2	72.5 ± 4.5	90.8 ± 4.2	n.d.	n.d.	n.d.
1:320	4.5	84.7 ± 1.1	77.8 ± 4.4	93.4 ± 5.9	n.d.	n.d.	n.d.

CHO-K1 cells were transfected with receptor constructs and cAMP accumulation was determined with the ALPHAScreen™ technology (see Material and Methods). Data is given as mean ± SEM of three independent experiments each performed in triplicates.
