# Supplementary material for: Metabolites of lactic acid bacteria present in fermented foods are highly potent agonists of human hydroxycarboxylic acid receptor 3
Source: PLoS Genet. 2019 May 23;15(5):e1008145. doi: 10.1371/journal.pgen.1008145 (PMC6532841; doi:10.1371/journal.pgen.1008145)
Supplement: S6 Table — (PDF) [file pgen.1008145.s013.pdf]

Supplementary Table S6

Comparison of manually curated RNA-Sequencing data versus reported FPKM for HCAR2 and HCAR3

Experiment Accession	Experiment Title	Sample Accession	HCAR2 (FPKM)	HCAR3 (FPKM)	HCAR2 (P)	HCAR3 (P)	HCAR2 (NP)	HCAR3 (NP)	HCAR2 (T)	HCAR3 (T)	HCAR2 (P) HCAR3 (NP)	HCAR2 (NP) HCAR3 (P)	HCAR2 (P) HCAR3 (P)	HCAR2 (NP) HCAR3 (NP)
SRX1874029	GSM2212622: 10E smallbowel female control S1	SRS1523875	72.2	123.3	63	76	123	114	186	190	45	57	18	66
SRX1874030	GSM2212623: 10F rectum male Ulcerative Colitis S2	SRS1523876	48.6	82.3	35	40	63	59	98	99	21	26	14	37
SRX1874031	GSM2212624: 10G rightcolon female control S3	SRS1523877	94.5	39.1	54	32	34	58	88	90	42	20	12	14
SRX1874032	GSM2212625: 10H leftcolon male Ulcerative Colitis S4	SRS1523878	55.5	13.1	36	9	17	45	53	54	33	5	3	12
SRX1874033	GSM2212626: 10J ileumcecum female Crohn's disease S5	SRS1523879	13.0	26.9	26	38	30	18	56	56	13	25	13	5
SRX1874034	GSM2212627: 10K smallbowel female Crohn's disease S6	SRS1523880	39.5	21.8	27	7	31	56	58	63	27	7	0	24
SRX1874035	GSM2212628: 10L leftcolon male Ulcerative Colitis S7	SRS1523881	187.0	145.3	157	102	192	254	349	356	134	79	23	113
SRX1874036	GSM2212629: 10M rightcolon_noninflamed female Ulcerative Colitis S8	SRS1523882	24.8	33.6	31	39	52	44	83	83	25	33	6	19
SRX1874037	GSM2212630: 10O smallbowel male Crohn's disease S9	SRS1523883	23.5	12.4	36	22	20	37	56	59	31	14	5	6
SRX1874038	GSM2212631: 10P rightcolon male control S10	SRS1523884	8.6	7.7	7	6	11	13	18	19	6	5	1	6
SRX1874039	GSM2212632: 10Q leftcolon male Crohn's disease S11	SRS1523885	11.4	28.4	19	37	42	25	61	62	13	30	6	12
SRX1874040	GSM2212633: 10R sigmoid male control S12	SRS1523886	8.9	5.5	14	8	13	19	27	27	11	5	3	8
SRX1874041	GSM2212634: 10S ileum male Crohn's disease S13	SRS1523887	143.0	189.3	191	197	280	283	471	480	142	148	49	132
SRX1874042	GSM2212635: 10U sigmoid male Crohn's disease S14	SRS1523888	68.9	96.5	117	121	135	138	252	259	82	83	35	52
SRX1874043	GSM2212636: 10V rightcolon female control S15	SRS1523889	27.5	48.4	26	31	45	40	71	71	17	22	9	23
SRX1874044	GSM2212637: 10W rightcolon female control S16	SRS1523890	12.0	5.1	7	3	20	24	27	27	6	2	1	18
SRX1874045	GSM2212638: 11B ileocolic male control S17	SRS1523891	17.2	24.9	23	27	32	28	55	55	18	22	5	10

SRX1874046	GSM2212639: 11E smallbowel female control S18	SRS1523892	58.0	71.1	100	108	122	117	222	225	71	78	29	44
SRX1874047	GSM2212640: 11I leftcolon female Ulcerative Colitis S19	SRS1523893	44.1	58.4	39	33	77	86	116	119	30	24	9	53
SRX1874048	GSM2212641: 11L leftcolon male Ulcerative Colitis S20	SRS1523894	1328.3	1924.4	927	1017	1725	1697	2652	2714	680	770	247	955
SRX1874049	GSM2212642: 11P rightcolon female Ulcerative Colitis S21	SRS1523895	46.1	55.7	66	50	84	107	150	157	48	32	18	52
SRX1874050	GSM2212643: 11R rightcolon male control S22	SRS1523896	22.7	14.1	23	10	14	29	37	39	18	5	5	9
SRX1874051	GSM2212644: 11S rightcolon female control S23	SRS1523897	1.7	2.6	3	5	5	3	8	8	2	4	1	1
SRX1874052	GSM2212645: 11T rightcolon female Crohn's disease S24	SRS1523898	94.4	106.2	141	117	148	183	289	300	92	64	49	84
SRX1874053	GSM2212646: 11U smallbowel female control S25	SRS1523899	18.8	9.4	78	33	45	97	123	130	64	19	14	26
SRX1874054	GSM2212647: 11W rightcolon female control S26	SRS1523900	16.2	4.2	26	8	15	34	41	42	22	4	4	11
SRX1874055	GSM2212648: 12F_6M leftcolon male control S27	SRS1523901	10.1	12.3	19	20	23	22	42	42	13	14	6	9
SRX1874056	GSM2212649: 12H ileum female Crohn's disease S28	SRS1523902	84.7	109.0	150	158	250	249	400	407	112	118	38	132
SRX1874057	GSM2212650: 12L rightcolon male Ulcerative Colitis S29	SRS1523903	7.7	11.1	10	10	18	20	28	30	6	6	4	12
SRX1874058	GSM2212651: 12M leftcolon male Ulcerative Colitis S30	SRS1523904	127.7	91.7	175	121	156	211	331	332	130	76	45	80
SRX1874059	GSM2212652: 12O smallbowel female Crohn's disease S31	SRS1523905	58.9	70.7	22	17	28	33	50	50	21	16	1	12
SRX1874060	GSM2212653: 12P rightcolon male Ulcerative Colitis S32	SRS1523906	4.5	4.5	6	4	7	9	13	13	4	2	2	5
SRX1874061	GSM2212654: 12R rightcolon female control S33	SRS1523908	4.8	4.8	3	3	12	12	15	15	3	3	0	9
SRX1874062	GSM2212655: 12S rightcolon male control S34	SRS1523907	12.9	27.0	12	24	32	20	44	44	8	20	4	12
SRX1874063	GSM2212656: 12T sigmoid female Ulcerative Colitis S35	SRS1523909	147.5	172.0	560	448	829	970	1389	1418	421	309	139	519
SRX1874064	GSM2212657: 12U leftcolon female control S36	SRS1523910	28.0	14.5	29	14	23	40	52	54	23	8	6	15
SRX1874065	GSM2212658: 12W smallbowel male control S37	SRS1523911	34.1	75.3	44	74	111	85	155	159	31	60	13	51
SRX1874066	GSM2212659: 1C sigmoid female control S38	SRS1523912	8.4	19.3	16	20	20	16	36	36	5	9	11	11
SRX1874067	GSM2212660: 1D_7D sigmoid female control S39	SRS1523913	98.1	50.9	99	51	94	142	193	193	86	38	13	56
SRX1874068	GSM2212661: 1F ileum female control S40	SRS1523915	6.6	3.9	6	3	7	10	13	13	4	1	2	6

SRX1874069	GSM2212662: 1I sigmoid male control S41	SRS1523914	2.6	2.1	5	4	7	8	12	12	3	2	2	5
SRX1874070	GSM2212663: 1M rectum male Ulcerative Colitis S42	SRS1523916	118.0	111.3	130	111	153	179	283	290	105	84	25	69
SRX1874071	GSM2212664: 1O smallbowel female Crohn's disease S43	SRS1523917	82.7	114.6	101	99	141	147	242	246	82	80	19	61
SRX1874072	GSM2212665: 1P leftcolon male Ulcerative Colitis S44	SRS1523918	117.0	143.1	140	146	218	219	358	365	114	119	26	99
SRX1874073	GSM2212666: 1R colon male control S45	SRS1523919	11.4	3.4	9	2	6	13	15	15	9	2	0	4
SRX1874074	GSM2212667: 1T colon female control S46	SRS1523920	2.3	1.2	4	1	3	7	7	8	4	0	0	3
SRX1874075	GSM2212668: 1Y colon male control S47	SRS1523921	6.5	5.4	7	6	4	6	11	12	5	3	2	1
SRX1874076	GSM2212669: 2D rightcolon male control S48	SRS1523922	25.6	26.9	21	20	40	42	61	62	11	10	10	30
SRX1874077	GSM2212670: 2E smallbowel male control S49	SRS1523923	21.5	32.9	22	25	39	40	61	65	12	15	10	24
SRX1874078	GSM2212671: 2F sigmoid male control S50	SRS1523924	5.2	0.0	4	0	1	5	5	5	4	0	0	1
SRX1874079	GSM2212672: 2I sigmoid male control S51	SRS1523925	1.8	3.6	4	2	6	8	10	10	2	0	2	6
SRX1874080	GSM2212673: 2J ileum male Crohn's disease S52	SRS1523926	46.1	58.2	104	101	131	140	235	241	75	70	29	61
SRX1874081	GSM2212674: 2K ileum male Crohn's disease S53	SRS1523927	89.0	61.5	108	76	57	94	165	170	75	38	33	19
SRX1874082	GSM2212675: 2L smallbowel male Crohn's disease S54	SRS1523928	59.5	121.5	60	95	215	184	275	279	38	71	22	144
SRX1874083	GSM2212676: 2M rectosigmoid female control S55	SRS1523929	11.2	14.2	10	9	20	23	30	32	7	6	3	14
SRX1874084	GSM2212677: 2N leftcolon female control S56	SRS1523930	2.4	2.4	1	1	1	1	2	2	1	1	0	0
SRX1874085	GSM2212678: 2P ileum male Crohn's disease S57	SRS1523931	10.3	7.3	15	13	18	20	33	33	10	8	5	10
SRX1874086	GSM2212679: 2Q rightcolon_inflamed female Crohn's disease S58	SRS1523932	16.4	24.5	22	20	37	39	59	59	17	15	5	22
SRX1874087	GSM2212680: 2R rightcolon female control S59	SRS1523933	4.3	3.4	5	3	7	9	12	12	5	3	0	4
SRX1874088	GSM2212681: 2S leftcolon female Crohn's disease S60	SRS1523934	65.7	117.7	102	115	209	208	311	323	75	88	27	121
SRX1874089	GSM2212682: 2T rightcolon female control S61	SRS1523935	0.8	0.8	2	1	2	3	4	4	1	0	1	2
SRX1874090	GSM2212683: 2Y sigmoid male control S62	SRS1523936	5.3	3.3	9	3	6	12	15	15	8	2	1	4
SRX1874091	GSM2212684: 3B sigmoid male control S63	SRS1523937	3.6	2.2	7	4	4	7	11	11	5	2	2	2
SRX1874092	GSM2212685: 3G transversecolon female control S64	SRS1523938	42.3	78.0	56	61	107	109	163	170	43	46	13	61

SRX1874093	GSM2212686: 3I leftcolon female Ulcerative Colitis S65	SRS1523939	37.9	53.6	32	37	35	32	67	69	21	26	11	9
SRX1874094	GSM2212687: 3K leftcolon female Ulcerative Colitis S66	SRS1523940	1090.9	1379.9	378	451	771	723	1149	1174	274	335	104	436
SRX1874095	GSM2212688: 3M smallbowel male control S67	SRS1523941	296.0	396.8	326	420	420	336	746	756	243	329	83	91
SRX1874096	GSM2212689: 3N colon male Crohn's disease S68	SRS1523942	66.7	53.5	69	59	62	74	131	133	54	42	15	20
SRX1874097	GSM2212690: 3R sigmoid male control S69	SRS1523943	3.7	6.7	8	10	9	7	17	17	5	7	3	2
SRX1874098	GSM2212691: 3S ileum male Crohn's disease S70	SRS1523944	28.3	34.4	40	47	41	36	81	83	31	36	9	5
SRX1874099	GSM2212692: 3W rectum male control S71	SRS1523945	17.0	21.9	13	14	33	34	46	48	13	14	0	19
SRX1874100	GSM2212693: 4D rightcolon female control S72	SRS1523946	17.0	25.8	22	29	43	38	65	67	16	23	6	20
SRX1874101	GSM2212694: 4E_9H ileum female Crohn's disease S73	SRS1523948	7.2	9.4	10	15	16	11	26	26	7	12	3	4
SRX1874102	GSM2212695: 4F rectum female control S74	SRS1523947	12.0	43.9	2	8	17	11	19	19	0	6	2	11
SRX1874103	GSM2212696: 4J smallbowel female Crohn's disease S75	SRS1523949	109.3	279.1	140	286	310	172	450	458	89	230	51	80
SRX1874104	GSM2212697: 4K leftcolon female Ulcerative Colitis S76	SRS1523950	130.7	157.7	241	184	325	391	566	575	189	130	52	195
SRX1874105	GSM2212698: 4L ileum female Crohn's disease S77	SRS1523951	13.2	16.8	18	13	36	41	54	54	14	9	4	27
SRX1874106	GSM2212699: 4M ileum male control S78	SRS1523952	25.2	10.9	33	11	18	40	51	51	24	2	9	16
SRX1874107	GSM2212700: 4P leftcolon male Ulcerative Colitis S79	SRS1523954	2.1	3.2	2	3	3	2	5	5	2	3	0	0
SRX1874108	GSM2212701: 4S smallbowel male Crohn's disease S80	SRS1523953	10.2	6.8	10	7	7	10	17	17	8	5	2	2
SRX1874109	GSM2212702: 4W leftcolon male Crohn's disease S81	SRS1523955	246.2	249.6	234	200	307	349	541	549	181	146	53	161
SRX1874110	GSM2212703: 4Y ileum female Crohn's disease S82	SRS1523957	555.8	812.1	509	522	686	704	1195	1226	359	359	150	327
SRX1874111	GSM2212704: 5D ileum male Crohn's disease S83	SRS1523956	24.4	26.2	29	34	40	35	69	69	19	24	10	16
SRX1874112	GSM2212705: 5J leftcolon male Ulcerative Colitis S84	SRS1523958	78.8	39.9	76	30	78	124	154	154	64	18	12	60
SRX1874113	GSM2212706: 5M leftcolon male Ulcerative Colitis S85	SRS1523959	167.1	163.5	230	212	198	222	428	434	168	145	62	53
SRX1874114	GSM2212707: 5N ileum female Crohn's disease S86	SRS1523960	7.3	16.0	13	22	30	21	43	43	5	14	8	16
SRX1874115	GSM2212708: 5P smallbowel male Ulcerative Colitis S87	SRS1523961	22.9	41.2	35	40	54	51	89	91	24	28	11	26

SRX1874116	GSM2212709: 5S smallbowel male Crohn's disease S88	SRS1523962	104.9	111.4	160	144	214	234	374	378	113	97	47	117
SRX1874117	GSM2212710: 6E rectosigmoid female control S89	SRS1523963	8.6	7.8	13	11	14	16	27	27	7	5	6	9
SRX1874118	GSM2212711: 6H smallbowel male Crohn's disease S90	SRS1523964	16.3	33.3	36	56	62	44	98	100	22	41	14	21
SRX1874119	GSM2212712: 6J leftcolon male Ulcerative Colitis S91	SRS1523965	272.4	394.4	409	487	501	430	910	917	286	358	123	143
SRX1874120	GSM2212713: 6N rectum male Ulcerative Colitis S92	SRS1523966	460.9	491.7	318	281	366	411	684	692	248	205	70	161
SRX1874121	GSM2212714: 6O rightcolon_noninflamed male Ulcerative Colitis S93	SRS1523967	13.7	16.1	27	24	38	43	65	67	18	15	9	23
SRX1874122	GSM2212715: 6P leftcolon female Ulcerative Colitis S94	SRS1523968	25.9	49.7	31	52	46	27	77	79	20	39	11	7
SRX1874123	GSM2212716: 6R colon male control S95	SRS1523969	6.0	4.5	4	2	3	5	7	7	4	2	0	1
SRX1874124	GSM2212717: 6S leftcolon male Crohn's disease S96	SRS1523970	73.1	34.6	28	15	10	24	38	39	18	5	10	5
SRX1874125	GSM2212718: 6T sigmoid female control S97	SRS1523971	7.1	27.8	16	35	43	25	59	60	10	28	6	15
SRX1874126	GSM2212719: 6U ileum male Crohn's disease S98	SRS1523972	229.7	270.3	363	323	411	458	774	781	245	205	118	206
SRX1874127	GSM2212720: 6W leftcolon female Crohn's disease S99	SRS1523973	223.3	297.4	458	518	725	688	1183	1206	339	389	119	336
SRX1874128	GSM2212721: 7C rectosigmoid female control S100	SRS1523974	19.3	12.9	46	27	37	56	83	83	34	15	12	22
SRX1874129	GSM2212722: 7E ileum female Crohn's disease S101	SRS1523975	20.4	13.9	34	14	47	67	81	81	30	10	4	37
SRX1874130	GSM2212723: 7H leftcolon male Ulcerative Colitis S102	SRS1523976	66.3	80.9	111	128	180	165	291	293	86	103	25	77
SRX1874131	GSM2212724: 7L leftcolon male Crohn's disease S103	SRS1523977	6.2	8.9	9	10	14	14	23	24	6	7	3	7
SRX1874132	GSM2212725: 7M leftcolon male Ulcerative Colitis S104	SRS1523978	84.0	67.0	83	45	90	131	173	176	70	32	13	58
SRX1874133	GSM2212726: 7R rightcolon female control S105	SRS1523980	49.5	42.7	81	75	54	61	135	136	52	46	29	8
SRX1874134	GSM2212727: 7S leftcolon female control S106	SRS1523979	21.0	20.2	31	29	34	36	65	65	24	22	7	12
SRX1874135	GSM2212728: 7T leftcolon female Ulcerative Colitis S107	SRS1523981	17.5	5.1	23	4	11	32	34	36	22	3	1	8
SRX1874136	GSM2212729: 7 transversecolon male control S108	SRS1523982	13.5	10.2	15	6	16	25	31	31	14	5	1	11
SRX1874137	GSM2212730: 7U rectosigmoid female control S109	SRS1523983	110.9	63.6	186	89	152	258	338	347	146	49	40	103
SRX1874138	GSM2212731: 7V rectosigmoid female control S110	SRS1523984	42.5	32.4	51	41	68	78	119	119	41	31	10	37

SRX1874139	GSM2212732: 7W ileum male Crohn's disease S111	SRS1523985	117.2	56.9	138	48	100	193	238	241	122	32	16	68
SRX1874140	GSM2212733: 8C rightcolon female control S112	SRS1523986	29.5	59.1	7	17	14	4	21	21	1	11	6	3
SRX1874141	GSM2212734: 8E smallbowel male Crohn's disease S113	SRS1523987	74.6	90.0	29	27	53	57	82	84	23	21	6	32
SRX1874142	GSM2212735: 8F ileum male Crohn's disease S114	SRS1523988	32.8	55.8	62	86	114	97	176	183	41	65	21	49
SRX1874143	GSM2212736: 8I leftcolon female Ulcerative Colitis S115	SRS1523989	169.3	249.4	245	332	410	333	655	665	183	265	62	145
SRX1874144	GSM2212737: 8J ileum male Crohn's disease S116	SRS1523990	314.7	324.5	392	386	781	815	1173	1201	287	281	105	500
SRX1874145	GSM2212738: 8K ileum female Crohn's disease S117	SRS1523991	57.3	77.6	37	41	88	89	125	130	25	29	12	59
SRX1874146	GSM2212739: 8L smallbowel female control S118	SRS1523992	24.3	20.2	21	14	30	37	51	51	11	4	10	26
SRX1874147	GSM2212740: 8M leftcolon male Ulcerative Colitis S119	SRS1523993	170.1	183.5	166	152	191	207	357	359	121	107	45	84
SRX1874148	GSM2212741: 8Q rightcolon female control S120	SRS1523994	2.4	0.0	5	3	2	4	7	7	2	0	3	2
SRX1874149	GSM2212742: 8S smallbowel male Crohn's disease S121	SRS1523995	64.0	113.5	79	100	140	125	219	225	57	75	22	65
SRX1874150	GSM2212743: 8 ileum male Crohn's disease S122	SRS1523996	332.1	401.7	94	120	195	173	289	293	70	95	24	100
SRX1874151	GSM2212744: 8U leftcolon female Ulcerative Colitis S123	SRS1523997	325.5	267.2	462	246	539	775	1001	1021	394	178	68	361
SRX1874152	GSM2212745: 8V leftcolon male control S124	SRS1523998	17.3	20.4	24	20	34	38	58	58	19	15	5	19
SRX1874153	GSM2212746: 8W transversecolon female control S125	SRS1523999	36.2	13.4	85	34	79	131	164	165	71	20	14	59
SRX1874154	GSM2212747: 9F transversecolon female control S126	SRS1524000	109.3	56.6	79	50	68	98	147	148	62	32	17	36
SRX1874155	GSM2212748: 9I_6I leftcolon male Ulcerative Colitis S127	SRS1524001	67.5	53.6	72	64	65	74	137	138	49	41	23	24
SRX1874156	GSM2212749: 9L sigmoid male control S128	SRS1524003	10.8	20.5	7	10	25	24	32	34	7	10	0	15
SRX1874157	GSM2212750: 9N ileum_slightlyinflamed female Ulcerative Colitis S129	SRS1524002	64.0	31.2	69	39	90	121	159	160	56	26	13	64
SRX1874158	GSM2212751: 9O leftcolon male Crohn's disease S130	SRS1524004	244.6	362.6	210	265	367	318	577	583	158	212	52	155
SRX1874159	GSM2212752: 9Q sigmoid male Crohn's disease S131	SRS1524005	1.9	6.6	4	10	7	2	11	12	1	7	3	0
SRX1874160	GSM2212753: 9R rightcolon female control S132	SRS1524006	26.4	23.4	32	27	32	39	64	66	23	17	9	15
SRX1874161	GSM2212754: 9S smallbowel male control S133	SRS1524007	79.5	89.7	111	96	170	193	281	289	82	67	29	103

SRX1874162	GSM2212755: 9V colon female control S134	SRS1524008	29.6	24.5	47	33	38	52	85	85	35	21	12	17
	total (all samples)				11851	11351	16724	17761	28575	29112	8836	8214	3015	8509

Using HCAR2 (NCBI Reference Sequence: NM_177551.3) and HCAR3 (NCBI Reference Sequence: NM_006018.2) as query sequences we performed an Sequence Read Archive Nucleotide BLAST (https://blast.ncbi.nlm.nih.gov/Blast.cgi) for all RNA-Sequencing samples (accesssion numbers SRX1874029 – SRX1874162, 134 samples total) belonging to Bioproject: PRJNA326727, (GEO Accession: GSE83687) selecting Megablast due to its fast and optimized algorithm for highly similar sequences.

Match-mismatch scores and gap opening-gap extending penalties are tuned to avoid low similarity alignments and high number of mismatches, insertions/deletions (match = 4, mismatch = -5, gap open = 6, gap extend = 5). After the alignment by Megablast the number of aligned reads to HCAR2 and HCAR3 are counted. In the case of multiple mappings of the same read to several positions, the alignment with highest %identity and longest alignment length is taken into account. Then collected number of reads from alignment results were classified according to their edit distance to HCAR2 and HCAR3 genes, i.e. reads that are mapped to HCAR3 perfectly but mapped to HCAR2 with mismatches/insertions/deletions and vice versa. (total numbers for all samples are given in the table). We are aware that by using Megablast querying the SRA database the common first steps of RNA-Seq analysis (like removing adapter sequences/over-represented sequences/low quality sequences/experimental artifacts from reads) are not employed which affects the mapping quality and explains the observed number of reads in our results compared to the calculated FPKM values from the study (Bioproject: PRJNA326727, GEO Accession: GSE83687) which were retrieved from the NCBI database [48]. HCAR2 (P): number of reads that are matched perfectly to HCAR2; HCAR3 (P): number of reads that are matched perfectly to HCAR3; HCAR2 (NP): number of reads that are non-perfectly matched to HCAR2; HCAR3 (NP): number of reads that are non-perfectly matched to HCAR3; HCAR2 (P) HCAR3 (NP): number of reads that are matched perfectly to HCAR2 but non-perfectly matched to HCAR3; HCAR2 (NP) HCAR3 (P): number of reads that are matched perfectly to HCAR3 but non-perfectly matched to HCAR2; HCAR2 (P) HCAR3 (P): number of reads that are perfectly matched to both genes; HCAR2 (NP) HCAR3 (NP) number of reads that are non-perfectly matched (containing mismatches/gaps) to both genes
