# Supplementary material for: Metabolites of lactic acid bacteria present in fermented foods are highly potent agonists of human hydroxycarboxylic acid receptor 3
Source: PLoS Genet. 2019 May 23;15(5):e1008145. doi: 10.1371/journal.pgen.1008145 (PMC6532841; doi:10.1371/journal.pgen.1008145)
Supplement: S7 Table — (PDF) [file pgen.1008145.s014.pdf]

Supplementary Table S7

TPM values as downloaded from https://www.ebi.ac.uk/gxa/home [8]

organism part/tissue/cell type	Expression Atlas	**HCAR1**	**HCAR2**	**HCAR3**
blood	E-MTAB-5214	0.1	97	42
neutrophil, mature	E-MTAB-3827	0.9	85	103
monocyte, CD14-positive, CD16-negative classical	E-MTAB-3827	0.4	72	82
neutrophil, segmented of bone marrow	E-MTAB-3827	0.6	53	58
dendritic cell, conventional	E-MTAB-3827	0.3	5	6
macrophage, alternatively activated	E-MTAB-3827	1	2	6
macrophage, inflammatory	E-MTAB-3827	0.1	3	5
macrophage	E-MTAB-3827	0.3	1	3
thymocyte, CD4-positive, alpha-beta	E-MTAB-3827	9	2	2
eosinophil, mature	E-MTAB-3827	0.2	1	2
granulocyte monocyte progenitor cell	E-MTAB-3819	0.8	0.1	2
hematopoietic stem cell	E-MTAB-3819	1	3	1
megakaryocyte cell, CD34-, CD41+, CD42+	E-MTAB-3819	0.7	1	1
T cell, CD8-positive, alpha-beta	E-MTAB-3827	0.2	0.7	0.7
metamyelocyte, neutrophilic	E-MTAB-3827	0.3	0.7	0.5
erythroblast	E-MTAB-3827	0.2	0.4	0.2
thymocyte, CD3-positive, CD4-positive, CD8-positive, double positive	E-MTAB-3827	1	0.1	0.1
B cell, CD38-negative naive	E-MTAB-3827	0.4	-	0.1
erythroblast	E-MTAB-3819	0.2	-	0.1
leukocyte	E-MTAB-513	0.5	36	11
lymphocyte, EBV-transformed	E-MTAB-5214	0.3	0.8	2
vermiform appendix	E-MTAB-2836	0.2	43	24
bone marrow	E-MTAB-2836	-	36	20
spleen	E-MTAB-2836	6	58	23
spleen	E-MTAB-4344	5	12	15
spleen	E-MTAB-5214	5	22	7
tonsil	E-MTAB-2836	0.2	25	14
lymph node	E-MTAB-513	1	6	2
lymph node	E-MTAB-2836	0.3	1	0.9
thymus	E-MTAB-3871	0.4	1	0.2
skin	E-MTAB-2836	0.5	48	21
skin	E-MTAB-5214	0.4	34	5
skin	E-MTAB-5214	0.4	31	4
urinary bladder	E-MTAB-2836	5	15	13
urinary bladder	E-MTAB-5214	1	3	0.6
lung	E-MTAB-4344	1	19	9
lung	E-MTAB-513	1	32	7
lung	E-MTAB-2836	1	35	6
lung	E-MTAB-5214	0.5	9	2
adipose tissue	E-MTAB-4344	7	17	5
adipose tissue	E-MTAB-2836	17	39	3

adipose tissue, subcutaneous	E-MTAB-5214	3	11	0.9
adipose tissue	E-MTAB-513	6	9	0.8
breast	E-MTAB-513	26	39	3
breast	E-MTAB-5214	11	8	1
vagina	E-MTAB-5214	0.8	21	1
ectocervix	E-MTAB-5214	2	0.7	0.1
endocervix	E-MTAB-5214	3	0.3	0.1
endometrium	E-MTAB-2836	1	0.8	-
ovary	E-MTAB-5214	0.6	0.2	-
ovary	E-MTAB-4344	2	0.3	0.3
ovary	E-MTAB-513	4	0.1	0.1
ovary	E-MTAB-2836	1	-	-
fallopian tube	E-MTAB-5214	2	0.5	0.2
fallopian tube	E-MTAB-2836	8	2	0.8
placenta	E-MTAB-3871	0.6	0.3	0.1
placenta	E-MTAB-2836	3	10	2
prostate gland	E-MTAB-5214	1	3	0.3
prostate gland	E-MTAB-513	7	9	3
prostate gland	E-MTAB-2836	5	3	0.7
testis	E-MTAB-5214	2	4	0.1
testis	E-MTAB-4344	2	4	0.3
testis	E-MTAB-3716	2	4	0.4
testis	E-MTAB-513	3	2	0.1
testis	E-MTAB-2836	2	5	0.3
salivary gland	E-MTAB-5214	3	13	0.5
salivary gland	E-MTAB-2836	7	3	0.6
esophagus	E-MTAB-2836	0.2	61	18
esophagus mucosa	E-MTAB-5214	0.3	45	5
stomach	E-MTAB-5214	2	0.7	0.1
stomach	E-MTAB-3871	4	-	-
stomach	E-MTAB-2836	3	1	0.3
small intestine	E-MTAB-3871	3	1	0.3
small intestine	E-MTAB-2836	0.1	2	0.4
duodenum	E-MTAB-2836	0.2	2	0.3
large intestine	E-MTAB-3871	3	0.9	0.1
colon	E-MTAB-513	2	0.8	-
colon, transverse	E-MTAB-5214	0.2	0.4	-
gall bladder	E-MTAB-2836	0.3	3	1
greater omentum	E-MTAB-5214	6	13	1
kidney, cortex	E-MTAB-5214	1	2	0.4
kidney	E-MTAB-4344	1	-	-
kidney	E-MTAB-3716	1	4	0.3
kidney	E-MTAB-513	3	1	0.4
kidney	E-MTAB-2836	2	1	0.3
kidney, left	E-MTAB-3871	8	0.4	0.2

kidney, right	E-MTAB-3871	8	0.6	0.3
kidney, renal cortex	E-MTAB-3871	7	0.3	0.2
kidney, renal cortex left	E-MTAB-3871	7	0.5	0.3
kidney, renal cortex, right	E-MTAB-3871	5	0.2	0.2
kidney, renal pelvis	E-MTAB-3871	7	0.3	0.1
kidney, renal pelvis left	E-MTAB-3871	7	0.6	0.2
kidney, renal pelvis, right	E-MTAB-3871	6	0.3	0.3
thyroid gland	E-MTAB-5214	3	0.6	0.2
thyroid gland	E-MTAB-513	3	0.4	0.2
thyroid gland	E-MTAB-2836	5	0.3	0.2
brain	E-MTAB-513	0.9	0.1	-
brain	E-MTAB-4344	2	-	0.1
temporal lobe	E-MTAB-3716	0.3	0.6	1
pituitary gland	E-MTAB-5214	0.8	0.4	0.1

Experiments included: E-MTAB-2836 (Raw Data Provider: The Human Protein Atlas), E-MTAB-5214 (The Genotype-Tissue Expression (GTEx) pilot analysis) [49] , E-MTAB-513 (RNA-Seq of human individual tissues and mixture of 16 tissues), E-MTAB-4344 (The ENCODE (Raw Data Provider: Encyclopedia of DNA Elements) Consortium), E-MTAB-3827 and E-MTAB-3819 (These studies makes use of data generated by the Blueprint Consortium. A full list of the investigators who contributed to the generation of the data is available fromwww.blueprint-epigenome.eu. Funding for the project was provided by the European Union's Seventh Framework Programme (FP7/2007-2013) under grant agreement no 282510 – BLUEPRINT.), E-MTAB-3871 (Integrative analysis of 111 reference human epigenomes.) [50], E-MTAB-3716 (The evolution of gene expression levels in mammalian organs.) [51]
