# Supplementary material for: Metabolites of lactic acid bacteria present in fermented foods are highly potent agonists of human hydroxycarboxylic acid receptor 3
Source: PLoS Genet. 2019 May 23;15(5):e1008145. doi: 10.1371/journal.pgen.1008145 (PMC6532841; doi:10.1371/journal.pgen.1008145)
Supplement: S8 Table — (PDF) [file pgen.1008145.s015.pdf]

Supplementary Table S8

Sources of genomic DNA used for HCAR amplification

**species**		**source**
Gorilla gorilla	Western Gorilla	Dr T. Haaf, MPI Molecular Genetics Berlin, Germany;<br>Dr L. Vigilant, MPI, Leipzig, Germany
Homo sapiens	human	Dr M. Stoneking, MPI, Leipzig, Germany;<br>D. Sere, MPI, Leipzig, Germany
Mus musculus	house mouse	Dr A. Orth, University of Montpellier, France
Nomascus leucogenys	white-cheeked Gibbon	Dipl. Biol. C. Roos, Primate Center Göttingen, Germany
Pan paniscus	bonobo	Dr W. Enard, MPI, Leipzig, Germany;<br>Dr L. Vigilant, MPI, Leipzig, Germany
Pan troglodytes	chimpanzee	Dr T. Haaf, MPI Molecular Genetics Berlin, Germany;<br>Dr W. Enard, MPI, Leipzig, Germany
Pongo pygmaeus	Orangutan	Dr M. Rocchi, University of Bari, Italy;<br>Dr W. Enard, MPI, Leipzig, Germany
Symphalangus syndactylus	siamang	Dipl. Biol. C. Roos, Primate Center Göttingen, Germany;<br>Dr T. Haaf, MPI Molecular Genetics Berlin, Germany
