# Supplementary material for: Metabolites of lactic acid bacteria present in fermented foods are highly potent agonists of human hydroxycarboxylic acid receptor 3
Source: PLoS Genet. 2019 May 23;15(5):e1008145. doi: 10.1371/journal.pgen.1008145 (PMC6532841; doi:10.1371/journal.pgen.1008145)
Supplement: S9 Table — (PDF) [file pgen.1008145.s016.pdf]

Supplementary Table S9

Primers used for HCA_1, HCA_2 and HCA_3 ortholog amplification, sequencing and introduction of epitope tags

ID	sequence (5´- 3´)	purpose
789	gtgcaaatcaaagaactgctcctc	pcDps forward (amplification/sequencing)
790	cctggttctttccgcctcagaag	pcDps reverse (amplification/sequencing)
2291	cgccgcactagttcacttatcgtcatcgtccttatagtc	FLAG-uni-*Spe I*_AS
2292	cgccgggtacctcactcacttatcgtcatcgtccttatagtc	FLAg-uni-*KpnI*_AS
2285	cgcgaattccccaccatgtacccctacgacgtccccgactacgcc	HA-uni-Kozak-*Eco RI*_S
2289	cgccccgggcccaccatgtacccctacgacgtccccgactacgcc	HA-uni-Kozak-*Xma I*_S
250	CTGTTCATGTAGGTGAAGCTGAG	GPR81-AS-apes-830
251	TTGGCCGACTCCATGAT	GPR81-AS-apes-540
252	CCGACAGAATGAGAAGGATGC	GPR81-AS-apes-3UTR
253	ACTCACTTCAATCAACTGGAACCT	GPR81-AS-apes-3UTR2
254	GCGACCCGGTTCATCAT	GPR81-S-apes-660
255	CACCACGCGGTGAACACTAT	GPR81-S-apes-370
256	GGTGAGTGCTAACGCTCAGAT	GPR81-S-apes-5UTR
257	TTTCCTGGCTGAAGTTTCTCTTC	GPR81-S-apes-5UTR2
258	TCCCTGAGTCCATTTCTGCTAA	GPR109-AS-apes-3UTR2
259	CTTGCAACCAGTCTCCCACT	GPR109-AS-apes-3UTR
260	AAGAGATGATGGCTGCTGTCC	GPR109-AS-apes-440
261	ATGTAGGTGAAGCTGAGAGTGATAAAG	GPR109-AS-apes-820
262	GACTGGAAGTTTGGGGACATC	GPR109-S-apes-280
263	CGCTYCATCGGACYMMCT	GPR109-S-apes-5UTR
264	AGCCTGCGGCAGAGACA	GPR109-S-apes-640
265	CACTCATGAATCSGCACCA	GPR109-S-apes-ATG
266	GCTGATGCTCTTCATGTTGG	GPR109a-S-apes-310
267	TTCTGGATCGGCATCTTCTTCT	GPR109a-AS-apes-500
268	GGCTGGTGCTCTTCATGTTT	GPR109b-S-apes-310
269	CAGTGCCATTCTGGATCAGC	GPR109b-AS-apes-500
270	CAACACCCTGACATGACATAAAG	GPR109-S-apes-5UTR2
271	ACACACTTGGAGATCCCACTG	mouse-GPR81-AS-wo-TGA
272	GACAACGGGTCGTGCTGT	mouse-GPR81-S-wo-ATG
273	ACGAGATGTGGAAGCCAGATAAG	mouse-GPR109-AS-wo-TGA
274	AGCAAGTCAGACCATTTTCTAGTGATA	mouse-GPR109-S-wo-ATG
275	ccccgactacgccGACAACGGGTCGTGCTGT	mouse-GPR81-S-HA-adaptor
276	cgtcatcgtccttatagtcACACACTTGGAGATCCC	mouse-GPR81-AS-FLAG-adaptor
277	ccccgactacgccAGCAAGTCAGACCATTTTC	mouse-GPR109-S-HA-adaptor
278	cgtcatcgtccttatagtcACGAGATGTGGAAGCCA	mouse-GPR109-AS-FLAG-adaptor
279	ccccgactacgccTACAACGGrTCGTGCTG	GPR81S-apes-HA-adaptor
280	cgtcatcgtccttatagtcGTGCCACTCAACAATGT	GPR81AS-apes-wo-gibbon-orang-FLAG-adaptor
281	cgtcatcgtccttatagtcTTTGTCACACTGATGCC	GPR81AS-orang-FLAG-adaptor
282	cgtcatcgtccttatagtcCTCAACAATGTGGGGAT	GPR81AS-gibbon-FLAG-adaptor
283	ccccgactacgccAATCGGCACCATCTGCAG	GPR109-S-apes-wo-orang109b-HA-adaptor
284	ccccgactacgccAATCCGCACCATCCG	GPR109b-S-orang-HA-adaptor
285	cgtcatcgtccttatagtcAGGAGAGGTTGGGCC	GPR109-AS-apes-wo-orang109ab-FLAG-adaptor
286	cgtcatcgtccttatagtcAGGGGArGTTGGGC	GPR109-AS-orang109ab-FLAG-adaptor
287	tggcgaggcatatctgtgta	mouseGPR109-S-504
288	ccctcttgatcttggcatgt	mouseGPR109-AS-667
