# Supplementary material for: Metabolites of lactic acid bacteria present in fermented foods are highly potent agonists of human hydroxycarboxylic acid receptor 3
Source: PLoS Genet. 2019 May 23;15(5):e1008145. doi: 10.1371/journal.pgen.1008145 (PMC6532841; doi:10.1371/journal.pgen.1008145)
Supplement: S1 References — (PDF) [file pgen.1008145.s017.pdf]

Supporting References S1

1. Nei M, Kumar S. Molecular Evolution and Phylogenetics: Oxford University Press; 2000.
2. Tamura K, Stecher G, Peterson D, Filipski A, Kumar S. MEGA6: Molecular Evolutionary Genetics Analysis version 6.0. Mol Biol Evol. 2013;30(12):2725-9. Epub 2013/10/18. doi: 10.1093/molbev/mst197. PubMed PMID: 24132122; PubMed Central PMCID: PMC3840312.
3. Thompson JD, Higgins DG, Gibson TJ. CLUSTAL W: improving the sensitivity of progressive multiple sequence alignment through sequence weighting, position-specific gap penalties and weight matrix choice. Nucleic Acids Res. 1994;22(22):4673-80. PubMed PMID: 7984417.
4. Xu GC, Zhang LL, Ni Y. Enzymatic preparation of D-phenyllactic acid at high space-time yield with a novel phenylpyruvate reductase identified from Lactobacillus sp CGMCC 9967. Journal of biotechnology. 2016;222:29-37. doi: 10.1016/j.jbiotec.2015.12.011. PubMed PMID: ISI:000371459600011.
5. Mu W, Yu S, Zhu L, Zhang T, Jiang B. Recent research on 3-phenyllactic acid, a broad-spectrum antimicrobial compound. Appl Microbiol Biotechnol. 2012;95(5):1155-63. Epub 2012/07/12. doi: 10.1007/s00253-012-4269-8. PubMed PMID: 22782253.
6. Li L, Shin SY, Lee KW, Han NS. Production of natural antimicrobial compound D-phenyllactic acid using Leuconostoc mesenteroides ATCC 8293 whole cells involving highly active D-lactate dehydrogenase. Letters in Applied Microbiology. 2014;59(4):404-11. doi: 10.1111/lam.12293. PubMed PMID: ISI:000342574700008.
7. Hummel W, Schutte H, Kula MR. Large-Scale Production of D-Lactate Dehydrogenase for the Stereospecific Reduction of Pyruvate and Phenylpyruvate. Eur J Appl Microbiol. 1983;18(2):75-85. doi: Doi 10.1007/Bf00500828. PubMed PMID: ISI:A1983RK96200002.
8. Petryszak R, Keays M, Tang YA, Fonseca NA, Barrera E, Burdett T, et al. Expression Atlas update--an integrated database of gene and protein expression in humans, animals and plants. Nucleic Acids Res. 2016;44(D1):D746-52. Epub 2015/10/21. doi: 10.1093/nar/gkv1045. PubMed PMID: 26481351; PubMed Central PMCID: PMC4702781.
9. van Baarlen P, Troost F, van der Meer C, Hooiveld G, Boekschoten M, Brummer RJ, et al. Human mucosal in vivo transcriptome responses to three lactobacilli indicate how probiotics may modulate human cellular pathways. Proc Natl Acad Sci U S A. 2011;108 Suppl 1:4562-9. Epub 2010/09/09. doi: 10.1073/pnas.1000079107. PubMed PMID: 20823239; PubMed Central PMCID: PMC3063594.
10. Paturi G, Phillips M, Jones M, Kailasapathy K. Immune enhancing effects of Lactobacillus acidophilus LAFTI L10 and Lactobacillus paracasei LAFTI L26 in mice. International journal of food microbiology. 2007;115(1):115-8. Epub 2006/12/01. doi: 10.1016/j.ijfoodmicro.2006.10.007. PubMed PMID: 17134782.
11. Valerio F, Lavermicocca P, Pascale M, Visconti A. Production of phenyllactic acid by lactic acid bacteria: an approach to the selection of strains contributing to food quality and preservation. FEMS microbiology letters. 2004;233(2):289-95. Epub 2004/04/06. doi: 10.1016/j.femsle.2004.02.020. PubMed PMID: 15063498.
12. Crowley S, Mahony J, van Sinderen D. Broad-spectrum antifungal-producing lactic acid bacteria and their application in fruit models. Folia microbiologica. 2013;58(4):291-9. Epub 2012/11/20. doi: 10.1007/s12223-012-0209-3. PubMed PMID: 23160868.
13. Murakami K, Habukawa C, Nobuta Y, Moriguchi N, Takemura T. The effect of Lactobacillus brevis KB290 against irritable bowel syndrome: a placebo-controlled double-blind crossover trial. BioPsychoSocial medicine. 2012;6(1):16. Epub 2012/08/07. doi: 10.1186/1751-0759-6-16. PubMed PMID: 22863114; PubMed Central PMCID: PMC3489517.
14. Riccia DN, Bizzini F, Perilli MG, Polimeni A, Trinchieri V, Amicosante G, et al. Anti-inflammatory effects of Lactobacillus brevis (CD2) on periodontal disease. Oral diseases. 2007;13(4):376-85. Epub 2007/06/20. doi: 10.1111/j.1601-0825.2006.01291.x. PubMed PMID: 17577323.
15. Maekawa T, Hajishengallis G. Topical treatment with probiotic Lactobacillus brevis CD2 inhibits experimental periodontal inflammation and bone loss. Journal of periodontal research. 2014;49(6):785-91. Epub 2014/02/04. doi: 10.1111/jre.12164. PubMed PMID: 24483135; PubMed Central PMCID: PMC4119090.
16. Zabat MA, Sano WH, Wurster JI, Cabral DJ, Belenky P. Microbial Community Analysis of Sauerkraut Fermentation Reveals a Stable and Rapidly Established Community. Foods. 2018;7(5). Epub 2018/05/15. doi: 10.3390/foods7050077. PubMed PMID: 29757214; PubMed Central PMCID: PMC5977097.
17. Lihua F, Lisbeth Truelstrup H. Fermentation and Biopreservation of Plant-Based Foods with Lactic Acid Bacteria. Handbook of Plant-Based Fermented Food and Beverage Technology: CRC Press; 2012.
18. Magnusson J. Antifungal activity of lactic acid bacteria. Acta Universitatis agriculturae Sueciae Agraria. 2003;397.

19. Mikelsaar M, Zilmer M. Lactobacillus fermentum ME-3 - an antimicrobial and antioxidative probiotic. Microbial ecology in health and disease. 2009;21(1):1-27. Epub 2009/04/22. doi: 10.1080/08910600902815561. PubMed PMID: 19381356; PubMed Central PMCID: PMC2670518.
20. Frick JS, Schenk K, Quitadamo M, Kahl F, Koberle M, Bohn E, et al. Lactobacillus fermentum attenuates the proinflammatory effect of Yersinia enterocolitica on human epithelial cells. Inflammatory bowel diseases. 2007;13(1):83-90. Epub 2007/01/09. doi: 10.1002/ibd.20009. PubMed PMID: 17206643.
21. Beloborodova N, Bairamov I, Olenin A, Shubina V, Teplova V, Fedotcheva N. Effect of phenolic acids of microbial origin on production of reactive oxygen species in mitochondria and neutrophils. Journal of biomedical science. 2012;19:89. Epub 2012/10/16. doi: 10.1186/1423-0127-19-89. PubMed PMID: 23061754; PubMed Central PMCID: PMC3503878.
22. Chen L, Bai Y, Fan TP, Zheng X, Cai Y. Characterization of a d-Lactate Dehydrogenase from Lactobacillus fermentum JN248 with High Phenylpyruvate Reductive Activity. J Food Sci. 2017;82(10):2269-75. Epub 2017/09/08. doi: 10.1111/1750-3841.13863. PubMed PMID: 28881036.
23. Schwenninger SM, Lacroix C, Truttmann S, Jans C, Sporndli C, Bigler L, et al. Characterization of low-molecular-weight antiyeast metabolites produced by a food-protective Lactobacillus-Propionibacterium coculture. Journal of food protection. 2008;71(12):2481-7. Epub 2009/02/28. PubMed PMID: 19244902.
24. Jia J, Mu W, Zhang T, Jiang B. Bioconversion of phenylpyruvate to phenyllactate: gene cloning, expression, and enzymatic characterization of D- and L1-lactate dehydrogenases from Lactobacillus plantarum SK002. Appl Biochem Biotechnol. 2010;162(1):242-51. Epub 2009/09/24. doi: 10.1007/s12010-009-8767-9. PubMed PMID: 19774350.
25. Okkers DJ, Dicks LM, Silvester M, Joubert JJ, Odendaal HJ. Characterization of pentocin TV35b, a bacteriocin-like peptide isolated from Lactobacillus pentosus with a fungistatic effect on Candida albicans. J Appl Microbiol. 1999;87(5):726-34. Epub 1999/12/14. PubMed PMID: 10594714.
26. van Baarlen P, Troost FJ, van Hemert S, van der Meer C, de Vos WM, de Groot PJ, et al. Differential NF-kappaB pathways induction by Lactobacillus plantarum in the duodenum of healthy humans correlating with immune tolerance. Proc Natl Acad Sci U S A. 2009;106(7):2371-6. Epub 2009/02/05. doi: 10.1073/pnas.0809919106. PubMed PMID: 19190178; PubMed Central PMCID: PMC2650163.
27. Walsh CJ, Guinane CM, O'Toole PW, Cotter PD. Beneficial modulation of the gut microbiota. FEBS letters. 2014;588(22):4120-30. Epub 2014/04/01. doi: 10.1016/j.febslet.2014.03.035. PubMed PMID: 24681100.
28. Herias MV, Hessle C, Telemo E, Midtvedt T, Hanson LA, Wold AE. Immunomodulatory effects of Lactobacillus plantarum colonizing the intestine of gnotobiotic rats. Clinical and experimental immunology. 1999;116(2):283-90. Epub 1999/05/26. PubMed PMID: 10337020; PubMed Central PMCID: PMC1905288.
29. Ahrne S, Hagslatt ML. Effect of lactobacilli on paracellular permeability in the gut. Nutrients. 2011;3(1):104-17. Epub 2012/01/19. doi: 10.3390/nu3010104. PubMed PMID: 22254077; PubMed Central PMCID: PMC3257727.
30. Strom K, Sjogren J, Broberg A, Schnurer J. Lactobacillus plantarum MiLAB 393 produces the antifungal cyclic dipeptides cyclo(L-Phe-L-Pro) and cyclo(L-Phe-trans-4-OH-L-Pro) and 3-phenyllactic acid. Applied and environmental microbiology. 2002;68(9):4322-7. Epub 2002/08/30. PubMed PMID: 12200282; PubMed Central PMCID: PMC124062.
31. Lavermicocca P, Valerio F, Evidente A, Lazzaroni S, Corsetti A, Gobbetti M. Purification and characterization of novel antifungal compounds from the sourdough Lactobacillus plantarum strain 21B. Applied and environmental microbiology. 2000;66(9):4084-90. Epub 2000/08/31. PubMed PMID: 10966432; PubMed Central PMCID: PMC92262.
32. Behera SS, Ray RC, Zdolec N. Lactobacillus plantarum with Functional Properties: An Approach to Increase Safety and Shelf-Life of Fermented Foods. BioMed Research International. 2018;2018:18. doi: 10.1155/2018/9361614.
33. Ilavenil S, Kim DH, Valan Arasu M, Srigopalram S, Sivanesan R, Choi KC. Phenyllactic Acid from Lactobacillus plantarum PromotesAdipogenic Activity in 3T3-L1 Adipocyte via Up-Regulationof PPAR-gamma2. Molecules. 2015;20(8):15359-73. Epub 2015/08/26. doi: 10.3390/molecules200815359. PubMed PMID: 26305241.
34. Smetankova J, Hladikova Z, Zimanova M, Greif G, Greifová M. Lactobacilli Isolated from Lump Sheep's Cheeses and their Antimicrobial Properties2014. 152-7 p.
35. Beganović J, Pavunc AL, Gjuračić K, Špoljarec M, Šušković J, Kos B. Improved Sauerkraut Production with Probiotic Strain Lactobacillus plantarum L4 and Leuconostoc mesenteroides LMG 7954. Journal of Food Science. 2011;76(2):M124-M9. doi: 10.1111/j.1750-3841.2010.02030.x.

36. Dinleyici EC, Dalgic N, Guven S, Metin O, Yasa O, Kurugol Z, et al. Lactobacillus reuteri DSM 17938 shortens acute infectious diarrhea in a pediatric outpatient setting. Jornal de pediatria. 2015;91(4):392-6. Epub 2015/05/20. doi: 10.1016/j.jped.2014.10.009. PubMed PMID: 25986615.
37. Jones SE, Versalovic J. Probiotic Lactobacillus reuteri biofilms produce antimicrobial and anti-inflammatory factors. BMC microbiology. 2009;9:35. Epub 2009/02/13. doi: 10.1186/1471-2180-9-35. PubMed PMID: 19210794; PubMed Central PMCID: PMC2653509.
38. Torres-Maravilla E, Lenoir M, Mayorga-Reyes L, Allain T, Sokol H, Langella P, et al. Identification of novel anti-inflammatory probiotic strains isolated from pulque. Appl Microbiol Biotechnol. 2016;100(1):385-96. Epub 2015/10/20. doi: 10.1007/s00253-015-7049-4. PubMed PMID: 26476654.
39. Zununi Vahed S, Barzegari A, Rahbar Saadat Y, Goreyshi A, Omidi Y. Leuconostoc mesenteroides-derived anticancer pharmaceuticals hinder inflammation and cell survival in colon cancer cells by modulating NF-kappaB/AKT/PTEN/MAPK pathways. Biomedicine & pharmacotherapy = Biomedecine & pharmacotherapie. 2017;94:1094-100. Epub 2017/08/20. doi: 10.1016/j.biopha.2017.08.033. PubMed PMID: 28821160.
40. Kuda T, Tomomi K, Kawahara M, Takahashi H, Kimura B. Inhibitory effects of Leuconostoc mesenteroides 1RM3 isolated from narezushi on lipopolysaccharide-induced inflammation in RAW264.7 mouse macrophage cells and dextran sodium sulphate-induced inflammatory bowel disease in mice. Journal of Functional Foods. 2014;6:631-6. doi: https://doi.org/10.1016/j.jff.2013.11.017.
41. Chaudhari SS, Gokhale DV. Phenyllactic Acid: A Potential Antimicrobial Compound in Lactic acid Bacteria. . J Bacteriol Mycol Open Access. 2016;2((5)).
42. Divyashri G, Krishna G, Muralidhara, Prapulla SG. Probiotic attributes, antioxidant, anti-inflammatory and neuromodulatory effects of Enterococcus faecium CFR 3003: in vitro and in vivo evidence. Journal of medical microbiology. 2015;64(12):1527-40. Epub 2015/10/10. doi: 10.1099/jmm.0.000184. PubMed PMID: 26450608.
43. Klingspor S, Bondzio A, Martens H, Aschenbach JR, Bratz K, Tedin K, et al. Enterococcus faecium NCIMB 10415 modulates epithelial integrity, heat shock protein, and proinflammatory cytokine response in intestinal cells. Mediators of inflammation. 2015;2015:304149. Epub 2015/05/08. doi: 10.1155/2015/304149. PubMed PMID: 25948884; PubMed Central PMCID: PMC4408629.
44. Menard S, Candalh C, Bambou JC, Terpend K, Cerf-Bensussan N, Heyman M. Lactic acid bacteria secrete metabolites retaining anti-inflammatory properties after intestinal transport. Gut. 2004;53(6):821-8. Epub 2004/05/13. PubMed PMID: 15138208; PubMed Central PMCID: PMC1774064.
45. Di Cagno R, Coda R, De Angelis M, Gobbetti M. Exploitation of vegetables and fruits through lactic acid fermentation. Food Microbiol. 2013;33(1):1-10. doi: 10.1016/j.fm.2012.09.003. PubMed PMID: ISI:000331130400001.
46. Placzek S, Schomburg I, Chang A, Jeske L, Ulbrich M, Tillack J, et al. BRENDA in 2017: new perspectives and new tools in BRENDA. Nucleic Acids Res. 2017;45(D1):D380-D8. Epub 2016/12/08. doi: 10.1093/nar/gkw952. PubMed PMID: 27924025; PubMed Central PMCID: PMC5210646.
47. Pasolli E, Schiffer L, Manghi P, Renson A, Obenchain V, Truong DT, et al. Accessible, curated metagenomic data through ExperimentHub. Nature methods. 2017;14(11):1023-4. Epub 2017/11/01. doi: 10.1038/nmeth.4468. PubMed PMID: 29088129; PubMed Central PMCID: PMC5862039.
48. Peters LA, Perrigoue J, Mortha A, Iuga A, Song WM, Neiman EM, et al. A functional genomics predictive network model identifies regulators of inflammatory bowel disease. Nature genetics. 2017;49(10):1437-49. Epub 2017/09/12. doi: 10.1038/ng.3947. PubMed PMID: 28892060; PubMed Central PMCID: PMC5660607.
49. Carithers LJ, Moore HM. The Genotype-Tissue Expression (GTEx) Project. Biopreservation and biobanking. 2015;13(5):307-8. Epub 2015/10/21. doi: 10.1089/bio.2015.29031.hmm. PubMed PMID: 26484569; PubMed Central PMCID: PMC4692118.
50. Roadmap Epigenomics C, Kundaje A, Meuleman W, Ernst J, Bilenky M, Yen A, et al. Integrative analysis of 111 reference human epigenomes. Nature. 2015;518(7539):317-30. Epub 2015/02/20. doi: 10.1038/nature14248. PubMed PMID: 25693563; PubMed Central PMCID: PMC4530010.
51. Brawand D, Soumillon M, Necsulea A, Julien P, Csardi G, Harrigan P, et al. The evolution of gene expression levels in mammalian organs. Nature. 2011;478(7369):343-8. Epub 2011/10/21. doi: 10.1038/nature10532. PubMed PMID: 22012392.

Supporting references S1

Fig S1 References [1-2], Fig S2 References [1-3], Fig S5 References [3-7], Fig S7 Reference [8], Table S1 References [6-45], Table S4 References [46-47], Table S6 Reference [48], Table S7 References [8, 49-51]
